# Dynamic Properties in a Collisional Model for Confined Granular Fluids: A Review

**DOI:** 10.3390/e28040454

**Published:** 2026-04-15

**Authors:** Ricardo Brito, Rodrigo Soto, Vicente Garzó

**Affiliations:** 1 Departamento de Estructura de la Materia, Física Térmica y Electrónica and GISC, Universidad Complutense de Madrid, E-28040 Madrid, Spain; brito@ucm.es; 2 Departamento de Física, Facultad de Ciencias Físicas y Matemáticas, Universidad de Chile, Santiago 8370448, Chile; rsoto@uchile.cl; 3 Departamento de Física and Instituto de Computación Científica Avanzada (ICCAEx), Universidad de Extremadura, Avda. de Elvas s/n, E-06006 Badajoz, Spain

**Keywords:** granular fluids, granular mixtures, Enskog/Boltzmann kinetic equation, confined systems, Navier–Stokes transport coefficients

## Abstract

Granular systems confined in a shallow box and subjected to vertical vibration provide an attractive geometry for studying fluidized granular media. In this configuration, grains acquire kinetic energy in the vertical direction through collisions with the confining walls, and this energy is subsequently transferred to the horizontal degrees of freedom via interparticle collisions. In recent years, the so-called Δ-model has been introduced as a simplified yet effective description of the dynamics of granular systems in such geometries. This review presents the results obtained from kinetic theory for the granular Δ-model. To model the energy transfer mechanism, a fixed velocity increment Δ is added to the normal component of the relative velocity during collisions. In this way, the vertical motion is effectively integrated out while retaining the collisional energy injection characteristic of the confined setup. This mechanism compensates for the energy loss due to inelastic collisions and leads to stable homogeneous steady states that can be analyzed within the framework of kinetic theory. The Enskog kinetic equation is formulated for this model and first analyzed in homogeneous steady states, yielding the stationary temperature and the equation of state. The dynamics of inhomogeneous states is then investigated using the Chapman–Enskog method, from which the Navier–Stokes transport coefficients are derived. The theory is further extended to granular mixtures, in which particles may differ in mass, size, restitution coefficient, or in the value of Δ. In this case, the phenomenology becomes richer; for example, energy equipartition is violated even in homogeneous steady states. The mixture dynamics is studied through the corresponding Navier–Stokes equations, and the associated transport coefficients are obtained in the low-density regime. The analysis of the hydrodynamic equations shows that, in agreement with simulations, the homogeneous state is linearly stable. Moreover, the intrinsically nonequilibrium nature of the model leads to the violation of Onsager reciprocity relations in granular mixtures. The theoretical predictions exhibit in general good agreement with both molecular dynamics simulations and direct simulation Monte Carlo results.

## 1. Introduction

Granular materials constitute a broad class of many-body systems whose macroscopic behavior emerges from dissipative interactions of the particles that are their constituents, called *grains* [1,2,3,4,5,6]. Unlike molecular systems, however, collisions between grains are intrinsically inelastic, leading to a continuous loss of kinetic energy [7,8]. This feature is responsible for the unusual behavior of granular systems and the main source of its strong phenomenology as opposed to their conservative counterparts [9,10]. As a consequence, granular fluids are inherently nonequilibrium systems: in the absence of external energy input, they cool down monotonically and eventually come to rest in the form of sand piles [10] or, in the absence of boundaries in microgravity experiments [11] or in simulations with periodic boundary conditions, via a nontrivial, nonhomogeneous state [12,13] that generates long-range correlations [14]. Sustained dynamical states therefore require some form of driving, which compensates for collisional dissipation and maintains a continuous motion and kinetic activity. The intrinsic nonequilibrium nature of granular matter, together with the energy input, that drives the system out of equilibrium even further, leads to a really powerful behavior [15]. In practice, different experimental realizations of driven granular systems correspond to different modes of energy input. Some examples of driving, such as avalanche flows on inclined plates [16,17], chute flows [18], rotating drums [19,20], vibrating boundaries [21], air-fluidization beds [22], sheared systems [23], horizontal shaking [24,25] or bulk forcing [26], result in different dynamical states, which are typically spatially inhomogeneous, with regions of high density, eventually in solid-like configurations. The choice of driving is therefore not just technical: it strongly influences the stationary states, transport properties, and stability of the system [6,27]. One class of driving is obtained by forcing via the boundaries, for instance, systems where energy is supplied through collisions with vibrating or moving walls [20,28,29,30,31,32] or computer simulation equivalent [33]. This type of driving is particularly relevant experimentally, but it introduces shock waves or boundary layers that complicate the theoretical description [34,35,36,37,38].

**Figure 1:**
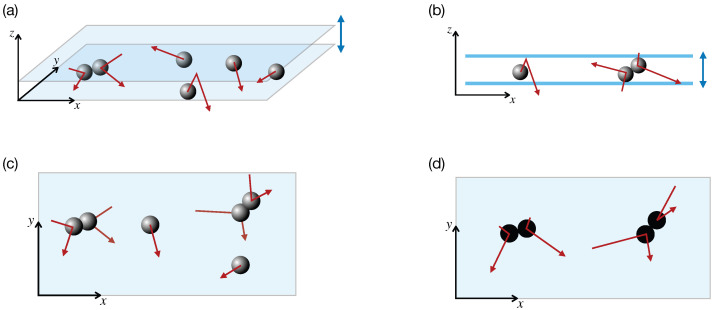
Conceptual motivation of the Δ-model. (**a**) Quasi-two-dimensional setup, where spherical grains are placed in a vertically vibrating shallow box. Grains can collide with the vibrating walls and among themselves. (**b**) Lateral view of the system. Grain collisions with the top and bottom walls inject energy into the vertical degrees of freedom, which is later transferred to the horizontal ones via grain–grain oblique collisions. (**c**) Top view of the quasi-two-dimensional system. As the height of the box is larger than the particle diameters, they can partially overlap at collisions when seeing from above. (**d**) In the Δ-model, the vertical motion is abstracted out [particles moving in the (x,y) plane, and are drawn in black], keeping its effect on injecting energy into the horizontal degrees of freedom. If particles reach the collision with a small relative velocity, the net effect is to gain energy but, if their normal relative velocity is large, inelasticity overcomes the injection and the collision is dissipative. Note that, in the Δ-model, particles move only in *x* and *y*, implying that there is no overlap and collisions take place when the distance is exactly equal to the particle diameter.

An important class of driven granular systems corresponds to the vertical vibration of quasi-two-dimensional (Q2D) systems [28,32,39,40,41,42,43,44,45]. In these systems, energy is injected through collisions with a vertically vibrating plate or shaker, especially in monolayers. A common way to make monolayers is to cover the experiment with a glass lid at a height slightly larger than a diameter grain. This configuration forces the particles to remain in the *quasi*-two-dimensional plane. Grain collisions with the lower and upper plate energize the *z*-component of the velocity [46]. While the vertical motion is directly excited by the driving, horizontal motion emerges indirectly through grain–grain collisions, which transfer energy from vertical to horizontal degrees of freedom (see Figure 1). Usually, the vertical dynamics is fast compared to the horizontal one, and therefore it is natural to seek an effective two-dimensional description in which the net effect of confinement and vibration will be encoded in modified collision rules for the horizontal velocities. The advantage of these systems is twofold. On the experimental side, particles are easy to track in a monolayer, just by placing a camera on top of the experimental setting. On the theoretical side, this system can be treated as purely two-dimensional, eliminating configurations where particles stack on top of each other. This Q2D setup is particularly relevant for the development of theories of granular matter as it directly controls the particle density, from very low gas-like regimes to dense solid-like states. Also, in a wide region of parameter space, the system reaches steady states that are statistically homogeneous in the planar directions, as observed both experimentally and in computer simulations [28,41,47,48]. They constitute, therefore, an excellent playground for studying granular hydrodynamic theories, which are normally built making gradient expansions around homogeneous states, contrary to the case of undriven granular gases, which can become uncontrollably inhomogeneous [13,49]. The Q2D experimental setup is the inspiration for the theoretical collisional model, the Δ-model [48], which we analyze in this review.

When the particles are sufficiently dilute and interact primarily through instantaneous binary collisions, granular matter can be described as a granular gas or fluid if the density is increased. In that spirit, and over the past few decades, kinetic theory has played a central role in the theoretical understanding of granular gases. By extending the tools originally developed for molecular fluids to dissipative dynamics, kinetic theory provides a mesoscopic description that connects microscopic collision rules with macroscopic transport and collective phenomena. Starting from the inelastic generalizations of the Boltzmann and Enskog equations, it has been possible to derive hydrodynamic equations, compute transport coefficients, analyze linear and nonlinear instabilities, and compare theoretical predictions with numerical simulations and experiments. Comprehensive accounts of these developments can be found in standard monographs [50,51,52] and reviews [53] of granular kinetic theory.

However, when writing a kinetic equation for a granular fluid, one faces the problem of how to model the driving. While the dissipative nature of collisions is well captured by a coefficient of normal restitution for the inelastic hard sphere (IHS) model, the mechanism by which energy is injected into the system is model-dependent. Modelization of transferal from vertical to horizontal degrees of freedom that takes place in the Q2D geometry is not an easy task. The parametrization of collisions in these confined conditions is cumbersome and hinders a straightforward form for the kinetic equation [46,54]. An alternative approach consists of building models that consider, in an effective way, the energy gain on the horizontal degrees of freedom in the Q2D geometry. Among these driving mechanisms are the so-called *thermostats*. These models are advantageous from the viewpoint of formulating kinetic equations [55,56]. One widely used approach consists of adding external forces acting on individual particles [57,58,59,60], such as stochastic (white-noise) forcing. Such models are analytically convenient when writing a kinetic equation, as energy injection acts on the particles, so they preserve homogeneity. Some of these drivings appear as additional Fokker–Planck terms in the kinetic equation and have been studied in great detail, including the derivation of steady state solutions [55], velocity distributions [56], or hydrodynamic descriptions [61,62,63,64,65], validated with computer simulations.

From a conceptual point of view, both thermostats and boundary driving introduce energy into the system through mechanisms that are external to the collisional dynamics between grains. Another option, inspired by the vertical-to-horizontal energy injection in the Q2D geometry (Figure 1b,c), is to develop models in which energy injection is incorporated more directly into the collision process itself with particles moving purely in two dimensions (Figure 1d). The first of these models considers random restitution coefficients with values smaller (dissipative) or larger (energy injection) than one [66]. However, the system lacks an intrinsic energy scale, and the total energy of the system behaves like a random walk, and therefore no stationary state is reached. Moreover, it does not reproduce the power law decay of the velocity distribution [67]. Such collisional models modify the binary collision rules so that collisions can either dissipate or inject energy, depending on the velocities of the colliding pair of particles. The advantage of these models is that they preserve the structure of the Boltzmann or Enskog equation, as they include the driving mechanism into the collision operator. The hope is that this modification still makes it possible to use standard techniques of kinetic theory to study such systems (driven steady states) without introducing external forces or boundary terms, and to analyze their properties within a unified kinetic theory framework. An alternative approach to modeling driven dissipative systems [68,69] is based on a hybrid framework, in which energy is injected during collisions while dissipation occurs during the free flight between them, instead via a normal restitution coefficient. More specifically, at each collision an amount ΔE>0 is added to the post-collisional kinetic energy. In contrast, viscous damping acts during the free-flight stage according to ${\dot{v}}_{i}=-\gamma v_{i}$, where γ is the friction coefficient. These articles derive hydrodynamic equations for the system. A key result is the emergence of hyperuniform states, which are locally disordered (fluid-like) yet exhibit long-range order akin to crystalline structures.

The so-called Δ-model [48] has emerged as a suitable description for the kinetic treatment of these confined Q2D systems. In this model, inelastic hard-sphere (or hard-disk) collisions, characterized by a normal restitution coefficient, are supplemented by an additional velocity increment of fixed magnitude Δ along the normal collision direction. Physically, this increment represents the effective transfer of kinetic energy from vertical to horizontal motion during interparticle collisions in the confined geometry. The Δ-model can be viewed as a minimal extension of the standard IHS model. It retains a binary collision structure of the collision term while maintaining momentum conservation. The inclusion of the Δ term that adds that amount of velocity serves as a thermostat that balances the dissipation of the normal restitution coefficient. As a result, the energy change per collision can be either negative or positive, depending on the pre-collisional state, and leads to a stationary, nonequilibrium, steady state. As a matter of fact, some collisions can dissipate energy (dissipation dominates) while some others gain energy (due to the Δ injection mechanism). The steady state is reached when these two contributions balance on average, so that the global rate of energy change vanishes and, consequently, the granular temperature attains a stationary asymptotic value. In this aspect, the Δ-model differs from the random restitution model in that, in the latter, the energy change is uncorrelated with the pre-collisional state, resulting in the absence of a well-defined steady state.

The steady state of the Δ-model for a single component is stable even for long wavelength perturbations, as opposite to freely cooling granular fluids, where long enough wavelength perturbations lead to vortex formation and clustering [5,13,70,71,72,73]. The stability manifests in the equation of state of the fluid, where the dependence on density and temperature on pressure factorizes [48]. It has then the inconvenience that the Δ-model cannot reproduce the clustering effects observed in some experiments. However, this stability allows one to control spatial gradients and to apply systematic hydrodynamic expansions in a manner closer to that of molecular fluids, in particular Champan–Enskog–like expansions [74], as will be shown in the present review. An extension of the Δ-model considers that each particle carries an internal variable which models the energy gained in the vertical direction since the last collision and the value of Δ depends on this variable. This results in an equation of state that presents a van der Waals loop, leading to a clustering instability [75].

Prior to a formal kinetic study of the Δ-model, its basic physical mechanisms and macroscopic equations were derived in Ref. [48]. There it was demonstrated that the system reaches a nonequilibrium steady state. The study of fluctuations around that state, via Landau–Placzeck theory, was carried out for the density and velocity fluctuations, putting emphasis on the relevance of the energy, strictly nonconserved, but that can be considered a quasi-conserved quantity. In a second study [76], the shear viscosity was derived by a simple linear response theory. Parallel to these developments, Brey and coworkers analyzed several aspects of the model, like the velocity distribution function [77], the hydrodynamic behavior [77,78], with special emphasis on the structure and stability of homogeneous steady states [79] and the existence of a normal or hydrodynamic solution [77]. The evolution equations for both the in-plane temperature and the *z*-component of the temperature were derived in Refs. [47,54,80], yielding explicit expressions that depend on the vibration frequency and the separation between the plates. Remarkably, the stationary temperature of the vibrated system qualitatively resembles that of the Δ-model (see, e.g., Figure 4 of Ref. [54]). References [81,82] compare the predictions of the Δ-model with computer simulations of the vertically driven system, and excellent agreement between simulations and the results of Δ-model is found. In contrast, alternative models such as the stochastic thermostat model [57] show a significantly worse agreement. These results support the conclusion that the Δ-model provides an accurate description of the vibrated monolayer outside the clustering regime. As a side remark, Ref. [80] also reports the appearance of the Mpemba effect in thin vibrated granular gases, in agreement with observation in other dissipative systems [83].

The Δ-model was then extended to mixtures of granular particles, where two or more species coexist [84]. The species may be distinguished by material properties, such as mass or diameter, or by dynamical ones, such as restitution coefficient or different values of the Δ parameter. The study of mixtures of granular materials was first addressed by Jenkins and Mancini in Ref. [85] by assuming the equipartition of energy (i.e., $T_{i}=T$, where $T_{i}$ is the partial temperature of species *i* and *T* is the global granular temperature). However, later studies [86] clearly show that granular mixtures present a phenomenology much stronger than that of (equilibrium) molecular mixtures since, under several types of forcing, energy equipartition is broken and each species reaches a different granular temperature [87,88,89]. Such lack of equipartition is quite general and is observed even in a single component between the translational and rotational degrees of freedom [90,91,92]. As expected, the Δ-model also displays this remarkable phenomenon [93]. Granular mixtures also exhibit various segregation phenomena, in which particles with similar properties may cluster [30] or preferentially migrate to different regions of the container [31], giving rise to effects such as the Brazil nut and reverse Brazil nut effects [94,95,96,97,98]. The Δ-model for mixtures shows analogous Brazil and reverse Brazil nut behavior [99,100].

More recently, the Δ-model has attracted renewed attention through a series of studies due to Foffi and coworkers. In a recent publication [101] they showed that a mixture of vibrated grains can form quasicrystals, and the Δ-model is a *bona fide* model to describe them. Moreover, the model presents long-range order [102,103], as evidenced by hyperuniformity [104]. Finally, the model has been used to study certain absorbing phases in granular systems [104,105] (introducing a friction term as in Refs. [68,69]), the coexistence between a fluid and a crystalline phase in granular fluids [106], and the dynamics of nonequilibrium interfaces [69]. Variations of the Δ-model include models where, instead of adding a fixed velocity, a fixed amount of energy is given at the collision [68]. The collision rules change but the main phenomenology of the Δ-model is preserved. An important variation is when Δ is made to depend on the time since last collision to induce phase separation [69,75,105]. The Δ-model can also be used as an appropriate description for a class of active matter where activity is not in the form of self-propulsion, but in the capacity to inject energy into the system. For example, when active spinners collide, the translational degrees of freedom effectively gain energy at the collision [68,107]. This energy injection has been cast into a variation of the Δ-model, where the additional velocity is in the tangential rather than in the normal direction, generating a chiral fluid with odd rheological properties [108]. These results demonstrate the versatility of the Δ-model in linking microscopic driving mechanisms with the emergence of complex macroscopic behavior.

The present review is organized as follows. In Section 2 we introduce the Δ-collisional model and formulate the corresponding Enskog kinetic equation, from where the balance equations are obtained. Section 3 is devoted to the analysis of homogeneous states, including the properties of the rate of energy and the existence of steady solutions. In Section 4 we apply the Chapman–Enskog method to derive the Navier–Stokes hydrodynamic equations and obtain explicit expressions for the transport coefficients. The extension of the kinetic equation to granular mixtures is presented in Section 5, where the analysis of time-dependent homogeneous states is performed. The derivation of the Navier–Stokes equations for mixtures and the calculation of the transport coefficients is performed in Section 6. Issues such as the breakdown of Onsager relations on granular mixtures and the stability of homogeneous states are addressed in Section 7. Finally, we summarize the main results and discuss open problems and possible directions for future research in Section 8.

## 2. Enskog Kinetic Equation for Collisional Model of Confined Granular Fluids

### 2.1. Collisional Model

We consider a granular fluid modeled as a gas of inelastic hard spheres of mass *m* and diameter σ. For the sake of simplicity, henceforth we will assume that the spheres are completely smooth and, so, the inelasticity of binary collisions is only characterized by a constant positive coefficient of normal restitution α≤1. The case α=1 corresponds to elastic collisions. In the case of smooth particles, the inelastic character of collisions only affects the translational degrees of freedom of grains. As mentioned in Section 1, we are interested here in analyzing the dynamic properties in *confined* granular fluids. However, due to the technical difficulties associated with the restrictions imposed by the confinement in the Boltzmann or Enskog collision operators [46,54], is it quite usual in the granular literature to adopt a coarse-grained approach in which the effect of confinement on grain dynamics is accounted for in an effective way. In this context, we consider in this paper a collisional model (the Δ-collisional model) proposed years ago by Brito et al. [48]. In this model, the factor Δ>0 is introduced in the scattering rules to mimic the transfer of kinetic energy from the vertical degrees of freedom of grains (which has been gained by the collisions of particles with the vibrating walls) to the horizontal ones. The relationship between the pre-collisional $\left(v_{1},v_{2}\right)$ and post-collisional $\left(v_{1}^{\prime },v_{2}^{\prime }\right)$ velocities in the Δ-model is [48]

(1) $$v_{1}^{\prime }=v_{1}-\frac{1}{2}\left(1+\alpha \right)\left(\hat{\sigma }\cdot g_{12}\right)\hat{\sigma }-\Delta \hat{\sigma },\;v_{2}^{\prime }=v_{2}+\frac{1}{2}\left(1+\alpha \right)\left(\hat{\sigma }\cdot g_{12}\right)\hat{\sigma }+\Delta \hat{\sigma }.$$

In Equation (1), $g_{12}=v_{1}-v_{2}$ is the relative velocity of the two colliding spheres, $\hat{\sigma }$ is a unit vector pointing from the center of particle 1 to the center of particle 2, and particles are approaching if $\hat{\sigma }\cdot g_{12}>0$. In addition, the parameter Δ is an extra velocity added to the relative motion. This extra velocity points outward in the normal direction $\hat{\sigma }$, as required by the conservation of angular momentum [109]. The relative velocity after collision is

(2) $$g_{12}^{\prime }=v_{1}^{\prime }-v_{2}^{\prime }=g_{12}-\left(1+\alpha \right)\left(\hat{\sigma }\cdot g_{12}\right)\hat{\sigma }-2\Delta \hat{\sigma },$$

so that it is quite simple to get the relation

(3) $$\left(\hat{\sigma }\cdot g_{12}^{\prime }\right)=-\alpha \left(\hat{\sigma }\cdot g_{12}\right)-2\Delta .$$

According to the collision rules (1), the total momentum is conserved in a binary collision ($v_{1}+v_{2}=v_{1}^{\prime }+v_{2}^{\prime }$) but the total kinetic energy is not conserved as expected. The change in kinetic energy upon collision is

(4) $$\Delta E\equiv \frac{m}{2}\left({v_{1}^{\prime }}^{2}+{v_{2}^{\prime }}^{2}-v_{1}^{2}-v_{2}^{2}\right)=m\left[\Delta^{2}+\alpha \Delta \left(\hat{\sigma }\cdot g_{12}\right)-\frac{1-\alpha^{2}}{4}{\left(\hat{\sigma }\cdot g_{12}\right)}^{2}\right].$$

It is quite apparent that (i) the right-hand side of Equation (4) vanishes for elastic collisions (α=1) and Δ=0 and that (ii) ΔE>0 (energy can be gained in collisions) or ΔE<0 (energy can be lost in collisions) depending on whether $\hat{\sigma }\cdot g_{12}$ is smaller than or larger than 2Δ/(1−α). Moreover, as we will show later, the average value of change in kinetic energy vanishes ($\langle \Delta E\rangle =0$) in the steady state. Thus, the injection of energy due to the parameter Δ and collision dissipation cancels out on average in the asymptotic steady state.

It is also convenient to consider the *inverse* or restituting collision where $\left(v_{1}^{\prime \prime },v_{2}^{\prime \prime }\right)$ are the pre-collisional velocities while $\left(v_{1},v_{2}\right)$ are the post-collisional velocities with the same collision vector $\hat{\sigma }$:

(5) $$v_{1}^{\prime \prime }=v_{1}-\frac{1}{2}\left(1+\alpha^{-1}\right)\left(\hat{\sigma }\cdot g_{12}\right)\hat{\sigma }-\alpha^{-1}\Delta \hat{\sigma },\;v_{2}^{\prime \prime }=v_{2}+\frac{1}{2}\left(1+\alpha^{-1}\right)\left(\hat{\sigma }\cdot g_{12}\right)\hat{\sigma }+\alpha^{-1}\Delta \hat{\sigma }.$$

According to Equation (5), the relationship between the relative velocities $g_{12}^{\prime \prime }=v_{1}^{\prime \prime }-v_{2}^{\prime \prime }$ and $g_{12}=v_{1}-v_{2}$ is

(6) $$g_{12}^{\prime \prime }=g_{12}-\left(1+\alpha^{-1}\right)\left(\hat{\sigma }\cdot g_{12}\right)\hat{\sigma }-2\alpha^{-1}\Delta \hat{\sigma }.$$

From Equation (6), one gets

(7) $$\left(\hat{\sigma }\cdot g_{12}^{\prime \prime }\right)=-\alpha^{-1}\left(\hat{\sigma }\cdot g_{12}\right)-2\alpha^{-1}\Delta .$$

Additionally, the volume transformation in velocity space for the direct collision $\left(v_{1},v_{2}\right)\to \left(v_{1}^{\prime },v_{2}^{\prime }\right)$ is

(8) $$dv_{1}^{\prime }dv_{2}^{\prime }=\alpha dv_{1}dv_{2},$$

while for the inverse collision $\left(v_{1}^{\prime \prime },v_{2}^{\prime \prime }\right)\to \left(v_{1},v_{2}\right)$ it is

(9) $$dv_{1}^{\prime \prime }dv_{2}^{\prime \prime }=\alpha^{-1}dv_{1}dv_{2}.$$

### 2.2. Enskog Kinetic Equation

It is well known that granular materials under rapid flow conditions admit a hydrodynamic-like description. The corresponding granular hydrodynamic equations can be obtained from a more fundamental point of view by using the tools of the classical kinetic theory of gases [52,74,110] conveniently adapted to dissipative dynamics [50,51]. Kinetic theory provides a *mesoscopic* description of matter, midway between a formal treatment based on Newton’s equations and a more phenomenological approach based on continuum mechanics. It has been widely employed by the engineering and physics community in the past decades to attempt to understand the behavior of granular matter. At a kinetic level, it is assumed that all the relevant information on the state of the granular fluid system is provided by the knowledge of the one-particle velocity distribution function f(r,v,t). This quantity is defined in such a way that f(r,v,t)drdv gives the average number of particles which at time *t* are located in dr around the point r and with velocities in the range dv around v.

For moderate densities, the Enskog kinetic equation is the natural extension of the usual Boltzmann equation for dilute gases. The former equation accounts for the effects of finite density in the dynamic properties of the gas. In the Δ-model and in the presence of the gravity acceleration g, the inelastic version of the Enskog equation is [111]

(10) $$\frac{\partial f}{\partial t}+v\cdot \nabla f+g\cdot \frac{\partial f}{\partial v}=J_{E}\left[r,v|f,f\right],$$

where the Enskog collision operator $J_{E}$ of the model reads

(11) $$\begin{array}{ccc} & & J_{E}\left[r,v_{1}|f,f\right]\equiv \sigma^{d-1}\int dv_{2}\int d\hat{\sigma }\Theta \left(-\hat{\sigma }\cdot g_{12}-2\Delta \right)\left(-\hat{\sigma }\cdot g_{12}-2\Delta \right)\\ & & \times \alpha^{-2}f_{2}\left(r,r+\sigma ,v_{1}^{\prime \prime },v_{2}^{\prime \prime };t\right)-\sigma^{d-1}\int \;dv_{2}\int d\hat{\sigma }\Theta \left(\hat{\sigma }\cdot g_{12}\right)\left(\hat{\sigma }\cdot g_{12}\right)\\ & & \times f_{2}\left(r,r+\sigma ,v_{1},v_{2};t\right). \end{array}$$

In Equation (11),

(12) $$f_{2}\left(r_{1},r_{2},v_{1},v_{2};t\right)\equiv \chi \left(r_{1},r_{2}\right)f\left(r_{1},v_{1};t\right)f\left(r_{2},v_{2};t\right),$$

$\chi (r_{1},r_{2})$ denotes the pair distribution function, Θ(x) is the Heaviside step function and *d* is the dimensionality of the system (d=2 for hard disks and d=3 for hard spheres). Note that, although the Δ-model attempts to describe confined quasi-two-dimensional systems (d=2), the kinetic theory exposed in this review is performed for an arbitrary number of dimensions *d*.

Similar to the Boltzmann equation, the Enskog equation assumes the molecular chaos hypothesis, which means it neglects velocity correlations among particles about to collide. One consequence of this hypothesis is that the two-body distribution function $f_{2}$ factorizes into the product of one-particle velocity distribution functions. However, unlike the Boltzmann equation, the Enskog equation accounts for (i) the spatial correlations between colliding pairs via the pair distribution function at contact χ(r,r+σ), and (ii) the variation of distribution functions over a distance equal to the diameter of grains (excluding volume effects). These two factors yield corrections to the Boltzmann results. In particular, there are non-vanishing collisional transfer contributions to the fluxes due to the spatial difference in the colliding spheres.

Given that here our main objective is to determine the dynamic properties of the granular fluid, we are interested in evaluating the collisional moments of the Enskog collision operator. In other words, we want to get an expression for I(ψ) where ψ is an arbitrary function of velocity and I(ψ) is defined as

(13) $$I\left(\psi \right)=\int \;dv_{1}\;\psi \left(v_{1}\right)J_{E}\left[r,v_{1}|f,f\right].$$

By following similar mathematical steps to those made for the conventional IHS model [50,51], I(ψ) can be rewritten in a more convenient way as [76,111]

(14) $$I_{\psi }=\sigma^{d-1}\int d\;v_{1}\int \;dv_{2}\int d\hat{\sigma }\;\Theta \left(\hat{\sigma }\cdot g_{12}\right)\left(\hat{\sigma }\cdot g_{12}\right)f_{2}\left(r,r+\sigma ,v_{1},v_{2};t\right)\left[\psi \left(v_{1}^{\prime }\right)-\psi \left(v_{1}\right)\right],$$

where $v_{1}^{\prime }$ is defined by Equation (1). Equation (14) gives the same result as for the IHS model [50].

### 2.3. Hydrodynamic Balance Equations

The relevant hydrodynamic fields of the granular gas can be defined as the first few velocity moments of the velocity distribution function f(r,v,t). The number density of particles n(r,t), the mean flow velocity U(r,t), and the granular temperature T(r,t) are given, respectively, by

(15) n(r,t)=∫dvf(r,v,t),

(16) $$U\left(r,t\right)=\frac{1}{n(r,t)}\int dvvf\left(r,v,t\right),$$

(17) $$T\left(r,t\right)=\frac{1}{dn(r,t)}\int dvmV^{2}f\left(r,v,t\right),$$

where V=v−U is the peculiar velocity.

The corresponding balance equations for the densities of mass, momentum and energy can be derived by using the relation (14). Their derivation follows similar mathematical steps to those made for the IHS model and adopts the standard form for rapid granular flows [112,113]. They are given by

(18) $$D_{t}n+n\nabla \cdot U=0,$$

(19) $$\rho D_{t}U+\nabla \cdot P=\rho g,$$

(20) $$D_{t}T+\frac{2}{dn}\left(\nabla \cdot q+P:\nabla U\right)=-\zeta T.$$

In Equations (18)–(20), $D_{t}\equiv \partial_{t}+U\cdot \nabla$ is the material derivative, ρ=mn is the mass density, and $\nabla_{i}\equiv \partial /\partial r_{i}$. As with molecular (elastic) fluids [74,110], the pressure tensor P(r,t) and the heat flux q(r,t) have both *kinetic* and *collisional transfer* contributions. Thus, $P=P_{k}+P_{c}$ and $q=q_{k}+q_{c}$. The kinetic contributions are given as usual by

(21) $$P_{k}\left(r,t\right)=\int \;dvmVVf\left(r,v,t\right),$$

(22) $$q_{k}\left(r,t\right)=\int \;dv\frac{m}{2}V^{2}Vf\left(r,v,t\right).$$

The collisional transfer contributions are [84]

(23) $$\begin{array}{ccc} P_{c} & = & \frac{1+\alpha }{4}m\sigma^{d}\int dv_{1}\int dv_{2}\int d\hat{\sigma }\Theta \left(\hat{\sigma }\cdot g_{12}\right)\left(\hat{\sigma }\cdot g_{12}\right)\hat{\sigma }\hat{\sigma }\left[\left(\hat{\sigma }\cdot g_{12}\right)+\frac{2\Delta }{1+\alpha }\right]\\ & & \times \int_{0}^{1}d\lambda f_{2}\left(r-\lambda \sigma ,r+\left(1-\lambda \right)\sigma ,v_{1},v_{2},t\right), \end{array}$$

(24) $$\begin{array}{ccc} q_{c} & = & \frac{1+\alpha }{4}m\sigma^{d}\int dv_{1}\int dv_{2}\int d\hat{\sigma }\Theta \left(\hat{\sigma }\cdot g_{12}\right){\left(\hat{\sigma }\cdot g_{12}\right)}^{2}\left(\hat{\sigma }\cdot G\right)\hat{\sigma }\\ & & \times \int_{0}^{1}d\lambda f_{2}\left[r-\lambda \sigma ,r+\left(1-\lambda \right)\sigma ,v_{1},v_{2},t\right]-\Delta \frac{m\sigma^{d}}{4}\\ & & \times \int dv_{1}\int dv_{2}\int d\hat{\sigma }\Theta \left(\hat{\sigma }\cdot g_{12}\right)\left(\hat{\sigma }\cdot g_{12}\right)\hat{\sigma }\left[\Delta +\alpha \left(\hat{\sigma }\cdot g_{12}\right)-2\left(\hat{\sigma }\cdot G\right)\right]\\ & & \times \int_{0}^{1}d\lambda f_{2}\left(r-\lambda \sigma ,r+\left(1-\lambda \right)\sigma ,v_{1},v_{2},t\right). \end{array}$$

Here, $G=\frac{1}{2}\left(V_{1}+V_{2}\right)$ is the velocity of the center of mass. Finally, the rate of energy ζ is given by

(25) $$\begin{array}{ccc} \zeta & = & -\frac{m}{dnT}\sigma^{d-1}\int dv_{1}\int dv_{2}\int d\hat{\sigma }\Theta \left(\hat{\sigma }\cdot g_{12}\right)\left(\hat{\sigma }\cdot g_{12}\right)\\ & & \times \left[\Delta^{2}+\alpha \Delta \left(\hat{\sigma }\cdot g_{12}\right)-\frac{1-\alpha^{2}}{4}{\left(\hat{\sigma }\cdot g_{12}\right)}^{2}\right]f_{2}\left(r,r+\sigma ,v_{1},v_{2},t\right). \end{array}$$

The rate of energy is due to competing effects of the energy injected by Δ and the energy lost by dissipative collisions. Thus, in contrast to the conventional IHS model where ζ it is always positive, in the Δ-model ζ can take negative values for small temperatures [see Equation (41) below]. This property allows the system to reach stable steady states. When Δ=0, Equations (23)–(25) reduce to those obtained in the IHS model [51].

It must be noted that in this paper we will assume the Einstein summation convention over repeated Greek indices. Additionally, when studying multicomponent granular systems, Latin indices will be used to label the particle species (running from 1 to *s*) and Greek indices will be used to label the spatial dimensions (d=2 for disks and d=3 for spheres). Also, Greek indices will be used to label the hydrodynamic modes when studying the linear stability of the homogeneous states.

As is well known, the macroscopic balance Equations (18)–(20) provide the basis for developing a hydrodynamic description of confined, dense granular fluids. However, as with elastic collisions [74,110], these equations are not a closed set of equations for the hydrodynamic fields *n*, U and *T*. To become a closed set, one has to express the momentum P and heat q fluxes as well as the rate of energy ζ in terms of the hydrodynamic fields and their spatial gradients. These types of equations are referred to as the constitutive equations for the fluxes and the rate of energy. To first order in spatial gradients, these equations are the Navier–Stokes–Fourier equations, and the corresponding expressions of the transport coefficients are obtained by solving the Enskog kinetic Equation (10) by means of the Chapman–Enskog method [74] conveniently adapted to account for inelastic collisions.

## 3. Homogeneous States

### 3.1. General Results

Before considering inhomogeneous states, it is convenient to analyze first homogeneous situations (∇→0). In this state and in the absence of a gravity field (g=0), the Enskog Equation (1) simply reduces to

(26) $$\frac{\partial f}{\partial t}=J_{E}\left[v|f,f\right]$$

where here

(27) $$\begin{array}{ccc} & & J_{E}\left[v_{1}|f,f\right]\equiv \sigma^{d-1}\chi \int dv_{2}\int d\hat{\sigma }\Theta \left(-\hat{\sigma }\cdot g_{12}-2\Delta \right)\left(-\hat{\sigma }\cdot g_{12}-2\Delta \right)\alpha^{-2}f\left(v_{1}^{\prime \prime },t\right)\\ & & \times f\left(v_{2}^{\prime \prime },t\right)-\sigma^{d-1}\chi \int \;dv_{2}\int d\hat{\sigma }\Theta \left(\hat{\sigma }\cdot g_{12}\right)\left(\hat{\sigma }\cdot g_{12}\right)f\left(v_{1},t\right)f\left(v_{2},t\right) \end{array}$$

is the Enskog collision operator for homogeneous states. According to Equation (27), since the pair correlation χ is constant, the Enskog collision operator (27) can be recognized as the Boltzmann collision operator for the Δ-model multiplied by χ. For homogeneous isolated systems, the mass and momentum balance Equations (18) and (19) are trivially satisfied and the energy balance Equation (20) becomes

(28) $$\frac{\partial T}{\partial t}=-T\zeta .$$

The rate of energy ζ for homogeneous states is given by

(29) $$\zeta =-\frac{m}{dnT}\sigma^{d-1}\chi \int dv_{1}\int dv_{2}\left[B_{1}g_{12}\Delta^{2}+B_{2}g_{12}^{2}\alpha \Delta -B_{3}g_{12}^{3}\frac{1-\alpha^{2}}{4}\right]f\left(v_{1},t\right)f\left(v_{2},t\right),$$

where for the angular integrations use has been made of the relation [55]

(30) $$B_{k}\equiv \int \;d\hat{\sigma }\;\Theta \left(\hat{\sigma }\cdot g\right){\left(\hat{\sigma }\cdot \hat{g}\right)}^{k}=\pi^{(d-1)/2}\frac{\Gamma \left(\frac{k+1}{2}\right)}{\Gamma \left(\frac{k+d}{2}\right)}$$

for positive integers *k*. In the IHS model (Δ=0), $\zeta \left(t\right)\propto \sqrt{T(t)}$ and the integration of Equation (28) leads to the well-known Haff’s cooling law [7]: $T\left(t\right)=T\left(0\right)/{\left(1+\frac{1}{2}\zeta \left(0\right)t\right)}^{2}$, T(0) being the initial temperature and ζ(0) is the energy rate at t=0. However, when Δ≠0, the time-dependence of ζ is more complex and, so, the time dependence of the granular temperature cannot analytically be obtained.

As in the homogeneous cooling state (HCS) for the IHS model, although the solution to the Enskog Equation (26) is not known to date, dimensional analysis and symmetry considerations suggest the existence of an isotropic in velocity space scaling solution where f(v,t) depends on time through the granular temperature T(t). This scaling solution is [77,111]

(31) $$f\left(v,t\right)=nv_{\mathrm{th}}{\left(t\right)}^{-d}\varphi \left(c,\Delta^{*}\right),$$

where $v_{\mathrm{th}}\left(t\right)=\sqrt{2T(t)/m}$ is the thermal velocity and φ is a reduced distribution whose dependence on *T* is encoded through the dimensionless velocity $c\equiv v/v_{\mathrm{th}}$ and the dimensionless parameter $\Delta^{*}\equiv \Delta /v_{\mathrm{th}}\propto T{\left(t\right)}^{-1/2}$. Thus, in contrast to the HCS, the unknown scaled distribution φ depends on the granular temperature *T* not only through the scaled velocity c but also through $\Delta^{*}\left(t\right)$. This is an additional intricacy of the Δ-model in comparison with the IHS model.

According to the solution (31), since the time-dependence of the distribution *f* is through *T*, then

(32) $$\frac{\partial f}{\partial t}=\frac{\partial f}{\partial T}\frac{\partial T}{\partial t}=-\zeta T\frac{\partial f}{\partial T}.$$

Additionally, *f* depends explicitly on *T* through the thermal velocity and implicitly through c and $\Delta^{*}$. As a consequence,

(33) $$T\frac{\partial f}{\partial T}=-\frac{1}{2}\frac{\partial }{\partial v}\cdot \left(vf\right)-\frac{1}{2}\Delta^{*}\frac{\partial f}{\partial \Delta^{*}},$$

and the Enskog Equation (26) reads

(34) $$\frac{1}{2}\zeta \frac{\partial }{\partial v}\cdot \left(vf\right)+\frac{1}{2}\zeta \Delta^{*}\frac{\partial f}{\partial \Delta^{*}}=J_{E}\left[v|f,f\right].$$

As said before, an exact solution to Equation (34) has not been found so far. However, a very good approximation can be obtained from an expansion in Sonine polynomials. In particular, the time-dependence of the kurtosis

(35) $$a_{2}=\frac{4}{d(d+2)}\int \;dc\;c^{4}\varphi \left(c\right)-1$$

of the scaled distribution φ has been widely studied in Refs. [77,111]. The analytical results derived in those works (which are based on the scaling solution (31)) exhibit good agreement with the numerical results obtained from the direct simulation Monte Carlo (DSMC) method [114].

Since the distribution function *f* is isotropic in velocity space, according to Equations (21)–(24), the pressure tensor is diagonal and the heat flux vanishes:

(36) $$P_{ij}=p\delta_{ij},\;q=0.$$

The hydrostatic pressure *p* can be written as $p=nTp^{*}$ where

(37) $$p^{*}=1+2^{d-2}\chi \phi \left(1+\alpha \right)+\frac{2^{d-1}\Gamma \left(\frac{d}{2}\right)}{\sqrt{\pi }\Gamma \left(\frac{d+1}{2}\right)}\chi \phi \Delta^{*}\int dc_{1}\int dc_{2}g_{12}^{*}\varphi \left(c_{1},t\right)\varphi \left(c_{2},t\right),$$

where $g_{12}^{*}\equiv g_{12}/v_{\mathrm{th}}$ and

(38) $$\phi =\frac{\pi^{d/2}}{2^{d-1}d\Gamma \left(d/2\right)}n\sigma^{d}$$

is the solid volume fraction. Note that, besides the standard ideal gas and excluded volume contributions to the pressure, there is a new term proportional to Δ in Equation (37). This term is due to the additional momentum transfer at collisions. Moreover, the expression (29) of ζ can be rewritten as

(39) $$\zeta =-\frac{2}{d}n\sigma^{d-1}v_{\mathrm{th}}\chi \int dc_{1}\int dc_{2}\varphi \left(c_{1}\right)\varphi \left(c_{2}\right)\left(B_{1}g^{*}\Delta^{*2}+B_{2}\alpha g^{*2}\Delta^{*}-\frac{1-\alpha^{2}}{4}B_{3}g^{*3}\right).$$

### 3.2. Homogeneous Steady States

As has been clearly demonstrated, numerical computations are generally required to solve the Δ-model in a homogeneous time-dependent state, essentially due to the intricate dependence of the scaling distribution φ on $\Delta^{*}\left(t\right)$. A detailed study of the $\Delta^{*}$-dependence of φ and $a_{2}$ for different initial conditions has been carried out in Refs. [77,111].

To obtain analytical results, one typically considers the long-time limit where $\Delta^{*}\left(t\right)$ achieves a constant value $\Delta^{*}$ independent of time. In this limiting case (homogeneous steady state, HSS), an explicit expression of the kurtosis in the vicinity of the steady state can be derived. For a two-dimensional granular gas, the dependence of $a_{2}$ on α was studied in Ref. [84] showing that the magnitude of $a_{2}$ never exceeds 0.103. Thus, in the steady state, contributions to ζ and $p^{*}$ coming from terms proportional to $a_{2}$ are generally negligible compared to the remaining contributions. As a consequence, for practical purposes, the integrals (37) and (39) involving the distribution φ can be computed by replacing it by its Maxwellian form $\varphi_{M}$:

(40) $$\varphi \left(c,\Delta^{*}\right)\to \varphi_{M}\left(c\right)=\pi^{-d/2}e^{-c^{2}}.$$

Within the Maxwellian approximation, the rate of energy ζ is given by

(41) $$\zeta_{M}=\frac{\sqrt{2}\pi^{\frac{d-1}{2}}}{d\Gamma \left(\frac{d}{2}\right)}n\sigma^{d-1}v_{\mathrm{th}}\chi \left(1-\alpha^{2}-2\Delta^{*2}-\sqrt{2\pi }\alpha \Delta^{*}\right),$$

while the (reduced) hydrostatic pressure $p^{*}$ is

(42) $$p_{M}^{*}=1+2^{d-2}\chi \phi \left(1+\alpha \right)+\frac{2^{d}}{\sqrt{2\pi }}\chi \phi \Delta^{*}.$$

In the steady state, $\partial_{t}T=0$, and so Equation (28) implies that ζ=0. According to Equation (41), the condition $\zeta_{M}=0$ yields a quadratic equation in $\Delta^{*}$ whose physical solution (i.e., $\Delta^{*}=0$ if α=1) provides the α-dependence of $\Delta^{*}$ in the Maxwellian approximation. This solution is

(43) $$\Delta_{M}^{*}\left(\alpha \right)=\frac{1}{2}\sqrt{\frac{\pi }{2}}\alpha \left[\sqrt{1+\frac{4(1-\alpha^{2})}{\pi \alpha^{2}}}-1\right].$$

Since $\Delta^{*}=\Delta /\sqrt{2T/m}$, at given values of α and Δ, Equation (43) gives the value of the stationary temperature. As expected, according to Equation (43), for elastic collisions (α=1) the steady state is only achieved for $\Delta_{M}^{*}=0$. The relationship (43) has been tested against molecular dynamics (MD) simulations showing excellent agreement with deviations smaller than 2%, except for small values of the coefficient of restitution and/or high densities [48]. At a fixed value of Δ note that Equation (43) predicts that the granular temperature diverges when α→1. This result has been verified in MD simulations of the Δ-model (see Figure 2 of Ref. [48]). It must be remarked that this sort of divergence has also been observed in MD simulations carried out in three-dimensional systems with vibrating walls (see Figure 4 of Ref. [54]), with the stationary temperature scaling as the wall velocity squared with a prefactor that depends on the height of the box. In addition, there is a qualitative agreement between the stationary temperature *T* obtained from MD simulations and its theoretical prediction derived from the Δ-model [48].

Since the dependence of $\zeta_{M}$ on the volume fraction ϕ is only through χ(ϕ) (see Equation (41)), $\Delta_{M}^{*}\left(\alpha \right)$ is independent of ϕ. Beyond the Maxwellian approximation to ζ, one expects that the energy rate can be also written as $\zeta =\chi \left(\phi \right)\bar{\zeta }\left(\Delta^{*},\alpha \right)$. Hence, the steady condition (ζ=0) provides an expression of $\Delta^{*}$ independent of density.

**Figure 2:**
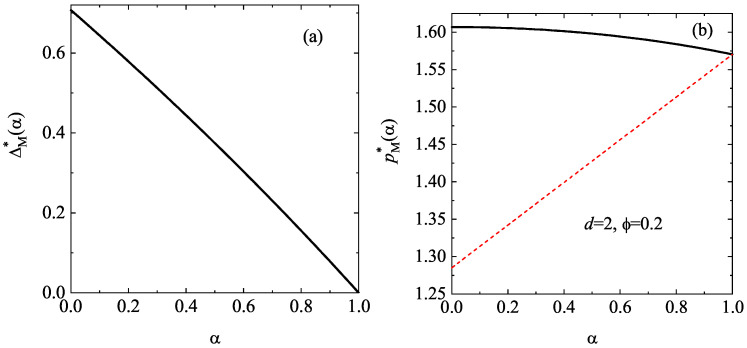
Panel (**a**): Plot of $\Delta_{M}^{*}$ versus the coefficient of restitution α in the steady state. Panel (**b**): Plot of the (reduced) pressure $p_{M}^{*}$ versus the coefficient of restitution α for a two-dimensional (d=2) system with a solid volume fraction ϕ=0.2. The solid line corresponds to the result obtained in the Δ-model while the dashed line refers to the result obtained in the IHS model ($\Delta^{*}=0$).

Panel (a) of Figure 2 shows the α-dependence of the (dimensionless) extra velocity $\Delta_{M}^{*}$. As expected, $\Delta_{M}^{*}$ increases with decreasing α. To complement panel (a) of Figure 2, the dependence of the (reduced) pressure $p_{M}^{*}$ on the coefficient of restitution α is illustrated by panel (b) of Figure 2 for d=2 and ϕ=0.2. A good approximation to the pair correlation χ for a two-dimensional gas is [85]

(44) $$\chi \left(\phi \right)=\frac{1-\frac{7}{16}\phi }{{\left(1-\phi \right)}^{2}}.$$

We have also included the prediction of the (reduced) pressure $p^{*}$ given by the IHS model [112,113]. We observe that the effect of inelasticity on the pressure is much more significant in the freely cooling gas of IHS than in the Δ-model.

## 4. Chapman–Enskog Method Applied to the Δ-Model

Once the homogeneous time-dependent state is well characterized, the next step is to obtain the Navier–Stokes hydrodynamic equations of the confined granular gas with explicit forms for the transport coefficients. To achieve this goal we solve the Enskog Equation (10) to first order in spatial gradients by means of a generalization of the conventional Chapman–Enskog method [74] to dissipative dynamics.

As widely discussed in many textbooks (see for instance, Refs. [74,110,115,116]), there are two separate stages in the relaxation of a *molecular* (elastic) fluid toward equilibrium. For times of the order of the mean free time, a first stage (*kinetic* regime) is identified where the effect of collisions is to quickly relax the fluid toward a local equilibrium state. This stage depends on the initial preparation of the system. A second stage is then identified in which the gas slowly evolves toward the total equilibrium. In this stage (referred to as the *hydrodynamic* regime), the gas has forgotten the microscopic details of its initial condition and its state is governed solely by the hydrodynamic fields. This special solution is referred to as a *normal* or hydrodynamic solution. These two stages are also expected in granular gases, except that in the kinetic regime relaxation occurs toward a time-dependent nonequilibrium distribution rather than a local equilibrium distribution. It is worth noting that, although the granular temperature *T* is not a conserved field due to the inelastic collisions, it is still assumed to be a slow hydrodynamic field (i.e., its time evolution is much slower than the remaining kinetic excitations). This assumption has been clearly confirmed by the good agreement found between theoretical predictions based on this hypothesis and computer simulations in different nonequilibrium problems [72,73,117,118,119,120,121,122,123,124,125,126,127,128,129].

According to the above scenario, in the hydrodynamic regime it is expected that the distribution function f(r,v,t) qualifies as a normal solution and, hence, it depends on space and time through a functional dependence on the hydrodynamic fields *n*, U, and *T*:

(45) f(r,v,t)=f[v|n(r,t),U(r,t),T(r,t)].

As discussed previously, although the temperature is not strictly a slow field, it has been shown in Ref. [78] in the context of the Δ-model for dilute granular gases that after a short transient period the distribution function does adopt a normal solution. A similar behavior is expected for dense granular fluids. As usual, the functional dependence (45) can be made local in space by means of an expansion in spatial gradients of the hydrodynamic fields. To generate it, *f* is written as a series expansion in a formal parameter ϵ measuring the nonuniformity of the system:

(46) $$f=f^{\left(0\right)}+\epsilon f^{\left(1\right)}+\epsilon^{2}f^{\left(2\right)}+\ldots ,$$

where each factor of ϵ means an implicit gradient of a hydrodynamic field. The fact that in the Δ-model the homogeneous steady state is stable for any inelasticity (see Refs. [48,79,130]) makes it possible to control the strength of spatial gradients through initial or boundary conditions, as occurs with molecular fluids. Thus, although the results obtained in the Navier–Stokes domain apply to sufficiently small gradients (low Knudsen number), they are not restricted *a priori* to small degree of dissipation.

Furthermore, in the presence of the gravity field g, it is also necessary to characterize the magnitude of gravity relative to spatial gradients. As for elastic collisions [74,110], the magnitude of g is assumed to be at least to first order in the perturbation expansion.

According to the expansion (46) for the distribution function, the Enskog collision operator and time derivative must also be expanded in powers of ϵ:

(47) $$J_{E}=J_{E}^{\left(0\right)}+\epsilon J_{E}^{\left(1\right)}+\ldots ,\;\partial_{t}=\partial_{t}^{\left(0\right)}+\epsilon \partial_{t}^{\left(1\right)}+\ldots .$$

The coefficients in the time derivative expansion are identified by a representation of the fluxes and the rate of energy in the macroscopic balance equations as a similar series through their definitions as functionals of *f*. The expansion (46) yields similar expansions for the momentum and heat fluxes, and the rate of energy when substituted into their definitions (21)–(25), respectively:

(48) $$P_{\lambda \beta }=P_{\lambda \beta }^{\left(0\right)}+\epsilon P_{\lambda \beta }^{\left(1\right)}+\ldots ,\;q=q^{\left(0\right)}+\epsilon q^{\left(1\right)}+\ldots ,$$

(49) $$\zeta =\zeta^{\left(0\right)}+\epsilon \zeta^{\left(1\right)}+\ldots .$$

In the zeroth-order approximation, $\partial_{t}^{\left(0\right)}n=\partial_{t}^{\left(0\right)}U_{\lambda }=0$ and $\partial_{t}^{\left(0\right)}T=-T\zeta^{\left(0\right)}$. Here, $\zeta^{\left(0\right)}$ is the zeroth-order contribution to the rate of energy. An approximate form of this quantity is given by Equation (41) in the HSS. Since the distribution $f^{\left(0\right)}\left(r,v,t\right)$ formally verifies the same Equation (34) for a strictly homogeneous state, $f^{\left(0\right)}$ is nothing more than the *local* version of the scaling solution (31), namely, it is given by Equation (31) except by the replacements n→n(r,t), v→v−U(r,t), and T→T(r,t). As a consequence, in the steady state, the local versions of the (approximate) expressions (41) and (42) provide the forms of $\zeta^{\left(0\right)}$ and $p^{*}$, respectively.

### 4.1. First-Order Approximation

The determination of the first-order distribution $f^{\left(1\right)}$ follows similar steps to those made in the IHS model (see for instance, chapter 3 of the textbook [51]), except that in the Δ-model there are new terms coming from the additional temperature dependence of $f^{\left(0\right)}$ through $\Delta^{*}$. The first-order velocity distribution function $f^{\left(1\right)}\left(r,v,t\right)$ is given by

(50) $$f^{\left(1\right)}=A\cdot \nabla \ln T+B\cdot \nabla \ln n+C_{\lambda \beta }\frac{1}{2}\left(\nabla_{\lambda }U_{\beta }+\nabla_{\beta }U_{\lambda }-\frac{2}{d}\delta_{\lambda \beta }\nabla \cdot U\right)+D\nabla \cdot U.$$

The quantities A(V), B(V), $C_{\lambda \beta }\left(V\right)$ and D(V) are the solutions of the following linear integral equations [84]:

(51) $$-\zeta^{\left(0\right)}T\frac{\partial A}{\partial T}-AT\frac{\partial \zeta^{\left(0\right)}}{\partial T}+LA=A,$$

(52) $$-\zeta^{\left(0\right)}T\frac{\partial B}{\partial T}+LB=B+\zeta^{\left(0\right)}\left(1+\phi \frac{\partial }{\partial \phi }\ln\chi \right)A,$$

(53) $$-\zeta^{\left(0\right)}T\frac{\partial C_{\lambda \beta }}{\partial T}+LC_{\lambda \beta }=C_{\lambda \beta },$$

(54) $$-\zeta^{\left(0\right)}T\frac{\partial D}{\partial T}+LD=D.$$

In Equations (51)–(54), we have introduced the linear operator L given by

(55) $$LX=-\left(J_{E}^{\left(0\right)}\left[f^{\left(0\right)},X\right]+J_{E}^{\left(0\right)}\left[X,f^{\left(0\right)}\right]\right),$$

where the operator $J_{E}^{\left(0\right)}$ is defined in Equation (27) with the replacements χ→χ(r,t) and $f\left(v;t\right)\to f^{\left(0\right)}\left(r,v;t\right)$. The inhomogeneous terms (which depend on $f^{\left(0\right)}$) in Equations (51)–(54) are

(56) $$A\left(V\right)=-VT\frac{\partial f^{\left(0\right)}}{\partial T}-\frac{p}{\rho }\left(1+T\frac{\partial }{\partial T}\ln p^{*}\right)\frac{\partial f^{\left(0\right)}}{\partial V}-K\left[T\frac{\partial f^{\left(0\right)}}{\partial T}\right],$$

(57) $$B\left(V\right)=-Vf^{\left(0\right)}-\frac{p}{\rho }\left(1+\phi \frac{\partial }{\partial \phi }\ln p^{*}\right)\frac{\partial f^{\left(0\right)}}{\partial V}-\left(1+\frac{1}{2}\phi \frac{\partial }{\partial \phi }\ln\chi \right)K\left[f^{\left(0\right)}\right],$$

(58) $$C_{\lambda \beta }\left(V\right)=V_{\lambda }\frac{\partial f^{\left(0\right)}}{\partial V_{\beta }}+K_{\lambda }\left[\frac{\partial f^{\left(0\right)}}{\partial V_{\beta }}\right],$$

(59) $$D\left(V\right)=\frac{1}{d}\frac{\partial }{\partial V}\cdot \left(Vf^{\left(0\right)}\right)+\left(\zeta_{U}+\frac{2}{d}p^{*}\right)T\frac{\partial f^{\left(0\right)}}{\partial T}+\frac{1}{d}K_{\lambda }\left[\frac{\partial f^{\left(0\right)}}{\partial V_{\lambda }}\right].$$

The operator K is given by [84]

(60) $$\begin{array}{ccc} K[X] & = & -\sigma^{d}\chi \int dv_{2}\int d\hat{\sigma }\Theta \left(-\hat{\sigma }\cdot g_{12}-2\Delta \right)\left(-\hat{\sigma }\cdot g_{12}-2\Delta \right)\hat{\sigma }\alpha^{-2}f^{\left(0\right)}\left(v_{1}^{\prime \prime }\right)X\left(v_{2}^{\prime \prime }\right)\\ & & +\sigma^{d}\chi \int dv_{2}\int d\hat{\sigma }\Theta \left(\hat{\sigma }\cdot g_{12}\right)\left(\hat{\sigma }\cdot g_{12}\right)\hat{\sigma }f^{\left(0\right)}\left(v_{1}\right)X\left(v_{2}\right). \end{array}$$

In Equation (59), $\zeta_{U}$ is defined through the expression

(61) $$\zeta^{\left(1\right)}=\zeta_{U}\nabla \cdot U.$$

In the low-density limit (ϕ=0), $p^{*}=1$, K[X]→0, and the integral Equations (51)–(59) reduce to those obtained in Ref. [78] for dilute granular gases. With respect to the rate of energy, in the limit ϕ→0, the quantity *D* becomes

(62) $$D=\zeta_{U}T\frac{\partial f^{\left(0\right)}}{\partial T}-\frac{1}{d}\Delta^{*}\frac{\partial f^{\left(0\right)}}{\partial \Delta^{*}},$$

and, hence, $\zeta_{U}\neq 0$ even for dilute granular gases. This contrasts with the results obtained in the IHS model [71]. However, for dense gases, $\zeta_{U}\neq 0$ for the IHS model [112,113].

### 4.2. Navier–Stokes Transport Coefficients

Based on symmetry considerations, the first-order contributions to the pressure tensor $P_{ij}^{\left(1\right)}$ and the heat flux $q^{\left(1\right)}$ are given, respectively, by

(63) $$P_{\lambda \beta }^{\left(1\right)}=-\eta \left(\nabla_{\lambda }U_{\beta }+\nabla_{\beta }U_{\lambda }-\frac{2}{d}\delta_{\lambda \beta }\nabla \cdot U\right)-\eta_{b}\nabla \cdot U\;\delta_{\lambda \beta },$$

(64) $$q^{\left(1\right)}=-\kappa \nabla T-\mu \nabla n.$$

In Equations (63)–(64), η is the shear viscosity, $\eta_{b}$ is the bulk viscosity, κ is the thermal conductivity, and μ is the diffusive heat conductivity coefficient. The coefficient μ is an additional transport coefficient not present in the elastic case. The contribution to the heat flux coming from the density gradient is also present in relativistic gases [131,132] as well as in ordinary (elastic) gases subjected to a drag force proportional to the particle velocity [133].

While the coefficients η, κ, and μ have kinetic and collisional contributions, the bulk viscosity has only collisional contributions and hence it vanishes in the low-density limit (ϕ→0). The kinetic contributions to the transport coefficients η, κ, and μ can be expressed in terms of the solutions of the set of linear integral Equations (51)–(53).

Given that the calculations to determine the Navier–Stokes transport coefficients are very long, here only some partial steps are offered in the calculation of the shear and bulk viscosities. Technical details to evaluate the remaining transport coefficients and the rate of energy can be found in Refs. [84,134,135].

### 4.3. Shear and Bulk Viscosities

As mentioned before, the shear viscosity η has kinetic and collisional contributions, i.e., $\eta =\eta_{k}+\eta_{c}$. However, the bulk viscosity $\eta_{b}=\eta_{b,c}$ since its kinetic contribution $\eta_{b,k}$ vanishes. The collisional contributions $\eta_{c}$ and $\eta_{b,c}$ can be obtained by expanding the expression (23) for the collisional pressure tensor to first order in spatial gradients. After some algebra, one gets the expressions [84]

(65) $$\eta_{b}=\frac{\pi^{d/2}}{2d^{2}\Gamma \left(\frac{d}{2}\right)}n^{2}\sigma^{d+1}m\chi v_{\mathrm{th}}\left[\frac{\left(d+1\right)}{2\sqrt{\pi }}\frac{\Gamma \left(\frac{d}{2}\right)}{\Gamma \left(\frac{d+3}{2}\right)}\left(1+\alpha \right)I_{\eta_{b}}+\Delta^{*}\right],$$

(66) $$\eta_{c}=\frac{\pi^{d/2}}{d\Gamma \left(\frac{d}{2}\right)}n\sigma^{d}\chi \left[\frac{1+\alpha }{d+2}+\frac{d}{\sqrt{\pi }\left(d+1\right)}\frac{\Gamma \left(\frac{d}{2}\right)}{\Gamma \left(\frac{d+1}{2}\right)}I_{\eta_{c}}\Delta^{*}\right]\eta_{k}+\frac{d}{d+2}\eta_{b},$$

where we have introduced the dimensionless integrals

(67) $$I_{\eta_{b}}=\int dc_{1}\int dc_{2}\;g_{12}^{*}\;\varphi \left(c_{1}\right)\varphi \left(c_{2}\right),$$

(68) $$I_{\eta_{c}}=\int dc_{1}\int dc_{2}\;g_{12}^{*-1}g_{12,x}^{*2}g_{12,y}^{*2}\varphi_{M}\left(c_{1}\right)\varphi_{M}\left(c_{2}\right).$$

It must be remarked that upon obtaining Equations (65)–(68) we have neglected the contributions proportional to $\zeta_{U}$ (it is expected that this quantity is in general very small). Furthermore, given that the unknown $C_{\lambda \beta }$ is also involved in the determination of $\eta_{c}$, we have replaced $C_{\lambda \beta }$ by its corresponding leading Sonine approximation. According to Equation (53), $C_{\lambda \beta }\propto C_{\lambda \beta }\propto mV_{\lambda }V_{\beta }$. Thus, the leading Sonine approximation to $C_{\lambda \beta }$ is given by

(69) $$C_{\lambda \beta }\left(V\right)\to -\frac{\eta_{k}}{nT^{2}}R_{\lambda \beta }\left(V\right)f_{M}\left(V\right),$$

where

(70) $$f_{M}\left(V\right)=n{\left(\frac{m}{2\pi T}\right)}^{d/2}e^{-\frac{mV^{2}}{2T}}$$

is the Maxwellian distribution and $R_{\lambda \beta }\left(V\right)$ is the traceless tensor

(71) $$R_{\lambda \beta }\left(V\right)=m\left(V_{\lambda }V_{\beta }-\frac{1}{d}\delta_{\lambda \beta }V^{2}\right).$$

We note that the Sonine expansion of $C_{\lambda \beta }\left(V\right)$ is different from the one usually employed for the zeroth-order distribution function $f^{\left(0\right)}\left(V\right)$ because the latter is isotropic in velocity space.

It only remains to evaluate the kinetic shear viscosity $\eta_{k}$. To get it, as usual, we multiply both sides of Equation (53) by $R_{ij}\left(V\right)$ and integrate over velocity. After some algebra, one achieves the result

(72) $$\left(-\zeta^{\left(0\right)}T\partial_{T}+\nu_{\eta }\right)\eta_{k}=-\frac{\int dV\;R_{\lambda \beta }\left(V\right)C_{\lambda \beta }\left(V\right)}{\left(d-1)(d+2\right)},$$

where

(73) $$\nu_{\eta }=\frac{\int dvR_{\lambda \beta }\left(V\right)LC_{\lambda \beta }\left(V\right)}{\int dvR_{\lambda \beta }\left(V\right)C_{\lambda \beta }\left(V\right)}.$$

In the hydrodynamic regime, the kinetic coefficient $\eta_{k}$ can be written as

(74) $$\eta_{k}\left(T\right)=\eta_{0}\left(T\right)\eta_{k}^{*}\left(\alpha ,\phi ,\Delta^{*}\right),$$

where

(75) $$\eta_{0}\left(T\right)=\frac{d+2}{8}\Gamma \left(\frac{d}{2}\right)\pi^{-\frac{d-1}{2}}\sigma^{1-d}\sqrt{mT}$$

is the low density value of the shear viscosity in the elastic limit. According to Equation (74), one has the identity

(76) $$T\partial_{T}\eta_{k}=\left(T\partial_{T}\eta_{0}\right)\eta_{k}^{*}-\frac{1}{2}\eta_{k}\Delta^{*}\frac{\partial \ln\eta_{k}^{*}}{\partial \Delta^{*}}=\frac{1}{2}\eta_{k}\left(1-\Delta^{*}\frac{\partial \ln\eta_{k}^{*}}{\partial \Delta^{*}}\right).$$

Thus, Equation (72) reads

(77) $$\frac{1}{2}\zeta^{\left(0\right)}\eta_{k}\Delta^{*}\frac{\partial \ln\eta_{k}^{*}}{\partial \Delta^{*}}+\left(\nu_{\eta }-\frac{1}{2}\zeta^{\left(0\right)}\right)\eta_{k}=nT-\frac{1}{\left(d-1)(d+2\right)}\int \;dvR_{\lambda \beta }\left(V\right)K_{\lambda }\left[\frac{\partial f^{\left(0\right)}}{\partial V_{\beta }}\right],$$

where use has been made of the explicit form (58) of $C_{\lambda \beta }$. As occurs for dilute granular gases [78], in contrast to the conventional IHS model, $\eta_{k}$ is given as the solution of an intricate first-order differential equation. The integral appearing on the right-hand side of Equation (77) can be computed as [84]

(78) $$\begin{array}{ccc} \int \;dvR_{\lambda \beta }\left(V\right)K_{\lambda }\left[\frac{\partial f^{\left(0\right)}}{\partial V_{\beta }}\right] & = & 2^{d-2}\left(d-1\right)\chi \phi \left(1+\alpha \right)\left(1-3\alpha \right)nT\\ & & +2^{d}\left(d-1\right)\chi \phi \Delta^{*}nT\left[\frac{\Gamma \left(\frac{d}{2}\right)}{\sqrt{\pi }\Gamma \left(\frac{d+1}{2}\right)}I_{\eta_{k}}-\Delta^{*}\right], \end{array}$$

where

(79) $$I_{\eta_{k}}=2\int dc_{1}\int dc_{2}\varphi \left(c_{1}\right)\varphi \left(c_{2}\right)\left[g_{12}^{*-1}\left(g_{12}^{*}\cdot c_{1}\right)-\left(1+\alpha \right)g_{12}^{*}\right].$$

(Expression (79) displayed here corrects a typo found in the previous result obtained in Ref. [136]. Moreover, Table 1 provides the correct forms for the complete set of Navier–Stokes transport coefficients.)

It is quite apparent that to obtain analytical expressions for η and $\eta_{b}$ one has to (i) consider the steady state ($\zeta^{\left(0\right)}=0$) and (ii) replace φ by its Maxwellian form. Under these approximations, for a two-dimensional system, one gets the following expressions for the (dimensionless) shear $\eta^{*}=\eta /\eta_{0}$ and bulk $\eta_{b}^{*}=\eta_{b}/\eta_{0}$ viscosities:

(80) $$\eta^{*}=\left[1+\frac{1}{2}\phi \chi \left(1+\alpha +\sqrt{\frac{2}{\pi }}\Delta_{M}^{*}\right)\right]\eta_{k}^{*}+\frac{1}{2}\eta_{b}^{*},$$

(81) $$\eta_{b}^{*}=\frac{8}{\pi }\phi^{2}\chi \left(1+\alpha +\sqrt{\frac{\pi }{2}}\Delta_{M}^{*}\right),$$

where

(82) $$\eta_{k}^{*}=\nu_{\eta }^{*-1}\left\{1-\frac{1}{4}\phi \chi \left[\left(1+\alpha \right)\left(1-3\alpha \right)-4\sqrt{\frac{2}{\pi }}\left(1+2\alpha \right)\Delta_{M}^{*}-4\Delta_{M}^{*2}\right]\right\},$$

and

(83) $$\nu_{\eta }^{*}=\frac{3}{8}\chi \left[\left(\frac{7}{3}-\alpha \right)\left(1+\alpha \right)+\frac{2\sqrt{2\pi }}{3}\left(1-\alpha \right)\Delta_{M}^{*}-\frac{2}{3}\Delta_{M}^{*2}\right].$$

Here, we recall that $\Delta_{M}^{*}$ is given by Equation (43). When $\Delta_{M}^{*}=0$ in Equations (80)–(83), one recovers the previous results derived for hard disks in the IHS model for vanishing energy rate ($\zeta^{\left(0\right)}=0$) [113,137].

### 4.4. Thermal Conductivity and Diffusive Heat Conductivity Coefficient

The determination of the thermal conductivity coefficient κ and the diffusive heat conductivity coefficient μ follows analogous steps to those shown before for the shear and bulk viscosities. Given that the calculations are very long, they will be omitted here. We refer the interested reader to Refs. [84,134,135] for more technical details of these calculations. In any case, for the sake of completeness, the explicit expressions of the relevant (scaled) transport coefficients are displayed in Table 1 for a two-dimensional system as functions of the density and the coefficient of restitution. In Table 1, $\kappa^{*}\left(\alpha \right)=\kappa \left(\alpha \right)/\kappa_{0}$ where $\kappa_{0}=\left(d\left(d+2\right)/2\left(d-1\right)\right)\left(\eta_{0}/m\right)$ is the low-density value of the thermal conductivity of an elastic gas. Moreover, $\zeta_{0}^{*}=\zeta_{M}/n\sigma v_{\mathrm{th}}$ and $\mu^{*}=n\mu /T\kappa_{0}$. Note that the coefficient $\mu^{*}$ vanishes for elastic collisions; for this reason $\mu^{*}\left(\alpha \right)$ has been scaled with respect to $\kappa^{*}\left(1\right)$ (the value of $\kappa^{*}$ for elastic collisions). Furthermore, the (scaled) heat diffusive coefficient $\mu^{*}$ also vanishes in the low-density regime (ϕ=0) when one neglects the contribution of the kurtosis $a_{2}$ since $\mu^{*}\propto a_{2}$ when ϕ=0.

**Figure 3:**
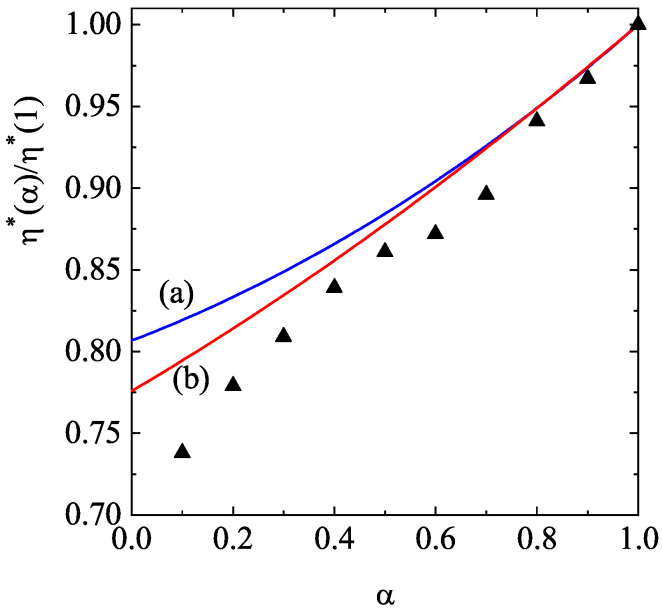
Plot of the (scaled) shear viscosity coefficient $\eta^{*}\left(\alpha \right)/\eta^{*}\left(1\right)$ versus the coefficient of restitution α for a two-dimensional granular gas (d=2) and two different values of the solid volume fraction ϕ: ϕ=0.1 (a) and ϕ=0.314 (b). The solid lines correspond to the kinetic theory results while symbols refer to MD simulations performed in Ref. [76] for ϕ=0.314.

Figure 3 illustrates the dependence of the (scaled) shear viscosity coefficient $\eta^{*}\left(\alpha \right)/\eta^{*}\left(1\right)$ for d=2 and two values of the solid volume fraction ϕ: ϕ=0.1 (dilute granular gas) and ϕ=0.314 (moderately dense granular gas). Here, $\eta^{*}\left(1\right)$ refers to the value of the (dimensionless) shear viscosity $\eta^{*}$ for elastic collisions. For the case of ϕ=0.314, the theoretical results from Equations (69)–(83) are compared with the results from MD simulations [76]. It is worthwhile remarking that the molecular dynamics method is based on Newton’s law equations and, hence, it avoids the usual assumptions involved in the kinetic theory description (molecular chaos and Equation (128) for accounting for spatial correlations at moderate densities). In this context, a comparison between kinetic theory and MD simulations is a quite stringent test for the former theory.

According to Figure 3, we observe that, for a given value of α, the shear viscosity (scaled with respect to its elastic value) decreases with increasing density. Moreover, for a given density, the shear viscosity decreases with increasing inelasticity. Regarding the comparison with MD simulations, we see that the (approximate) kinetic theory results qualitatively reproduce the simulation trends well, despite the relatively high gas density. At a more quantitative level, as inelasticity increases, the differences between theory and simulations become more significant, as expected.

**Figure 4:**
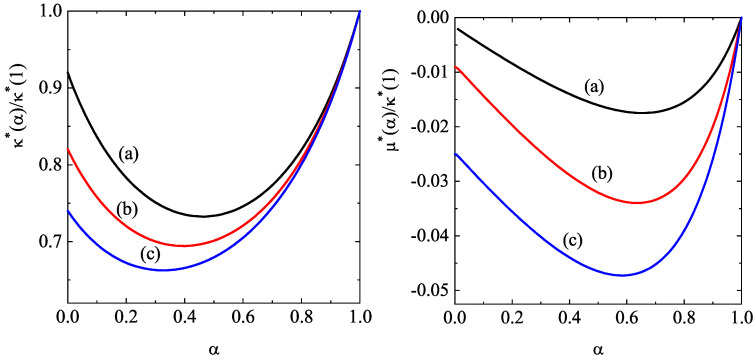
Plot of the (scaled) thermal conductivity $\kappa^{*}\left(\alpha \right)/\kappa^{*}\left(1\right)$ and diffusive heat conductivity $\mu^{*}\left(\alpha \right)/\kappa^{*}\left(1\right)$ coefficients versus the coefficient of restitution α for a two-dimensional granular gas (d=2) and three different values of the solid volume fraction ϕ: ϕ=0.1 (a), ϕ=0.2 (b), and ϕ=0.3 (c).

Figure 4 complements Figure 3 by showing the α-dependence of the (scaled) thermal conductivity coefficient $\kappa^{*}\left(\alpha \right)/\kappa^{*}\left(1\right)$ and the (scaled) diffusive heat conductivity coefficient $\mu^{*}\left(\alpha \right)/\kappa^{*}\left(1\right)$. Unlike the shear viscosity coefficient, we observe that the ratio $\kappa^{*}\left(\alpha \right)/\kappa^{*}\left(1\right)$ exhibits a non-monotonic dependence on α. Furthermore, dissipation and density have a greater impact on thermal conductivity than on shear viscosity. Regarding the coefficient $\mu^{*}$, we observe that this coefficient is negative and it is significantly affected by density. In any case, the magnitude of $\mu^{*}$ is quite small for any density and/or inelasticity; therefore, one can neglect the contribution to the heat flux coming from the density gradient. This means that, for practical purposes, one can assume that the heat flux obeys Fourier’s law in the Δ-model: $q^{\left(1\right)}=-\kappa \nabla T$. This conclusion contrasts with the results obtained in the conventional IHS model [51,71,112,138], since the coefficient μ is always positive, and its magnitude can exceed that of the thermal conductivity κ for strong collisional dissipation.

### 4.5. Stability of the HSS

Knowing the Navier–Stokes transport coefficients and the rate of energy makes it possible to analyze the stability of the HSS. This is a relevant state for confined, quasi-two-dimensional granular fluids. The HSS is a trivial solution of the Navier–Stokes hydrodynamic Equations (18)–(20) characterized by a uniform state with U=0 (without loss of generality) and a steady temperature $T_{H}$ determined from the equation $\zeta^{\left(0\right)}\left(n_{H},T_{H}\right)=0$. Here, the subscripts H denote the homogeneous steady state. This state has been widely studied in several previous papers [48,76,111] and the theoretical results compare quite well with computer simulations. Since the HSS has been proven stable for *dilute* granular gases [79], this subsection aims to investigate the stability of the HSS with respect to long enough wavelength perturbations at sufficiently high densities. To answer the above question, we will perform a linear stability analysis of the nonlinear Navier–Stokes hydrodynamic Equations (18)–(20) with respect to the HSS for *small* initial perturbations. The Navier–Stokes equations are obtained by substituting the constitutive Equations (63) and (64) into the balance Equations (18)–(21).

Near the HSS, we assume that the deviations $\delta y_{\alpha }\left(r,t\right)=y_{\alpha }\left(r,t\right)-y_{H\alpha }$ are small, where $\delta y_{\alpha }\left(r,t\right)$ denotes the deviation of {n,U,T,} from their values in the HSS. To compare with the results obtained in the IHS model [139], we consider here the same time and space variables: $\tau =\frac{1}{2}\nu_{H}t$ and $\ell =\frac{1}{2}\left(\nu_{H}/v_{0H}\right)r$, where $\nu_{H}=n_{H}T_{H}/\eta_{0H}$ and $v_{0H}=\sqrt{T_{H}/m}$. Here, $\eta_{0H}$ is given by Equation (75) with the replacement $T\to T_{H}$. The dimensionless time scale τ is a measure of the average number of collisions per particle in the time interval between 0 and *t*. The unit length $v_{0,H}/\nu_{H}$ is proportional to the time-independent mean free path of gas particles.

As usual, the linearized hydrodynamic equations for the perturbations

(84) $$\left\{\delta n(r;t),\delta U(r;t),\delta T(r;t)\right\}$$

are written in the Fourier space. A set of Fourier transformed dimensionless variables are then introduced as $\rho_{k}\left(\tau \right)=\delta n_{k}\left(\tau \right)/n_{H}$, $w_{k}\left(\tau \right)=\delta U_{k}\left(\tau \right)/v_{0H}$, $\theta_{k}\left(\tau \right)=\delta T_{k}\left(\tau \right)/T_{H}$, where $\delta y_{k\alpha }\equiv \left\{\delta \rho_{k},w_{k}\left(\tau \right),\theta_{k}\left(\tau \right)\right\}$ is defined as

(85) $$\delta y_{k\alpha }\left(\tau \right)=\int d\ell \;e^{-ik\cdot \ell }\delta y_{\alpha }\left(\ell ,\tau \right).$$

Note that in Equation (85) the wave vector k is dimensionless.

As occurs in the previous studies on molecular [140] and granular [71,139] fluids, linearization of the Navier–Stokes equations in $\rho_{k}$, $w_{k}$, and $\theta_{k}$ shows that the d−1 transverse velocity components $w_{k⊥}=w_{k}-\left(w_{k}\cdot \hat{k}\right)\hat{k}$ (orthogonal to the wave vector k) decouple from the other three modes. They obey the autonomous differential equation

(86) $$w_{k⊥}\left(\tau \right)=w_{k⊥}\left(0\right)e^{-\frac{1}{2}\eta^{*}k^{2}\tau },$$

where we have taken into account that $\eta^{*}$ does not depend on time in the HSS. Thus, since $\eta^{*}>0$ (see Equation (69)), the d−1 transversal shear modes $w_{k⊥}\left(\tau \right)$ are *linearly* stable.

The remaining (longitudinal) modes correspond to $\rho_{k}$, $\theta_{k}$, and the longitudinal velocity component of the velocity field, $w_{k||}=w_{k}\cdot \hat{k}$ (parallel to k). These modes are coupled and obey the equation

(87) $$\frac{\partial \delta y_{k\lambda }\left(\tau \right)}{\partial \tau }=M_{\lambda \beta }\delta y_{k\beta }\left(\tau \right),$$

where $\delta y_{k\alpha }\left(\tau \right)$ now denotes the set $\left\{\rho_{k},\theta_{k},w_{k||}\right\}$ and M is the square matrix [130]

(88) $$M=\left(\begin{array}{ccc} 0 & 0 & -ik\\ -\frac{d+2}{2(d-1)}\mu^{*}k^{2} & -2{\bar{\zeta }}_{0}-\frac{d+2}{2(d-1)}\kappa^{*}k^{2} & -ik\left(\frac{2}{d}p_{M}^{*}+\zeta_{U}\right)\\ -ikp_{M}^{*}C_{\rho } & -ik\left(p_{M}^{*}+\Psi_{p}\right) & -\frac{d-1}{d}\eta^{*}k^{2}-\frac{1}{2}\eta_{b}^{*}k^{2} \end{array}\right).$$

Here, $C_{\rho }\left(\phi \right)=1+\left(1+\phi \partial_{\phi }\ln\chi \right)\left(1-p_{M}^{*-1}\right)$, and it is understood that $p_{M}^{*}$, $\eta^{*}$, $\eta_{b}^{*}$, $\kappa^{*}$, $\mu^{*}$, and $\zeta_{U}$ are evaluated in the HSS. While $\kappa^{*}$ and $\mu^{*}$ were determined in Refs. [84,134,135], the first-order contribution to the rate of energy $\zeta_{U}$ was not evaluated. A good approximation to it for d=2 is [130]

(89) $$\zeta_{U}=\phi \chi \left[2\Delta_{M}^{*2}+\frac{2^{5/2}}{\sqrt{\pi }}\alpha \Delta_{M}^{*}-\frac{3}{2}\left(1-\alpha^{2}\right)\right].$$

Additionally, for a two-dimensional system, in Equation (88) we have introduced the dimensionless quantities

(90) $${\bar{\zeta }}_{0}\equiv T_{H}\left(\frac{\partial \zeta_{0}^{*}}{\partial T}\right)=\chi \Delta^{*}\left(\frac{1}{2}\sqrt{\frac{\pi }{2}}\alpha +\Delta_{M}^{*}\right),$$

(91) $$\Psi_{p}\equiv T_{H}\left(\frac{\partial p_{M}^{*}}{\partial T}\right)=-\sqrt{\frac{2}{\pi }}\phi \chi \Delta_{M}^{*}.$$

Here, we recall that $\zeta_{0}^{*}=\zeta_{M}/n_{H}\sigma \sqrt{2T_{H}/m}$ where $\zeta_{M}$ is given by Equation (41). For dilute granular gases (ϕ=0), Equations (88)–(91) are consistent with the results derived in Ref. [79] in the low-density limit.

**Figure 5:**
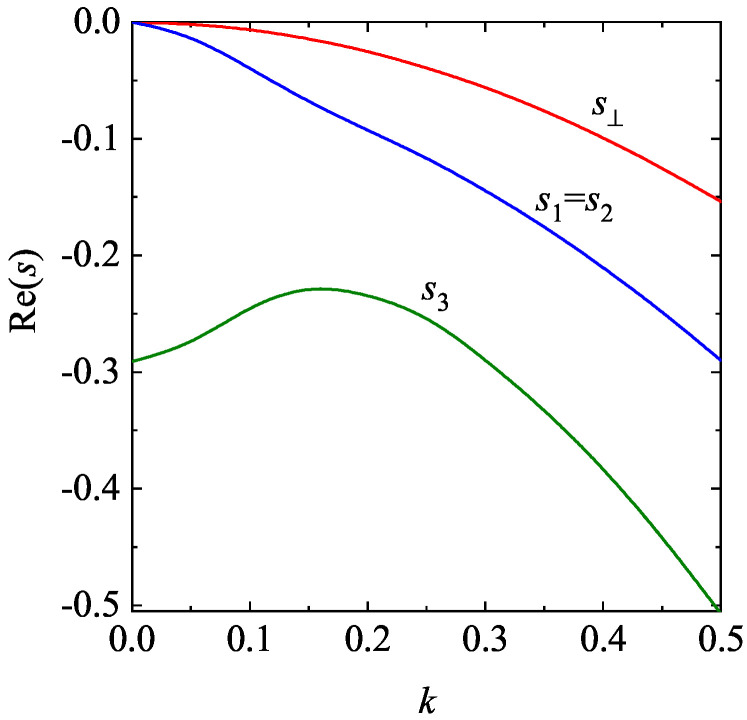
Dispersion relations for a granular two-dimensional fluid (d=2) with α=0.8 and ϕ=0.2. From top to bottom the curves correspond to the real parts of the shear (transversal) mode $s_{⊥}$ and the remaining three longitudinal modes ($s_{1}=s_{2}$ and $s_{3}$).

The longitudinal three modes have the form $\exp[s_{n}\left(k\right)\tau ]$ for n=1,2,3, where $s_{n}\left(k\right)$ are the eigenvalues of the matrix M. For given values of the coefficient of restitution α and the density ϕ, for k≠0 we find that one of the modes is real while the other two are a complex conjugate pair of propagating modes. Furthermore, an analysis of the eigenvalues of the matrix M for finite *k* and moderate densities shows that in general Re(sn)≤0 and hence the HSS is linearly *stable* in the complete range of values of the wave number *k* studied. As an illustration, the dispersion relations $s_{n}\left(k\right)$ for a two-dimensional granular fluid with α=0.8 and ϕ=0.2 are plotted in Figure 5. Only the real parts of the eigenvalues are represented. We observe that the real part of the “heat” mode $s_{3}$ exhibits a a non-monotonic dependence on the (dimensionless) wave number *k* while the other modes ($s_{1}=s_{2}$ and $s_{⊥}$) decrease with increasing *k*.

## 5. Granular Mixtures

### 5.1. Granular Mixtures: Enskog Kinetic Equation

Granular materials are usually present in nature or industry as polydisperse systems. The extension of the Enskog equation to granular mixtures within the context of the Δ-model is straightforward. We consider an *s*-multicomponent granular mixture of inelastic, smooth hard disks (d=2) or spheres (d=3) of masses $m_{i}$ and diameters $\sigma_{i}$. Collisions among all pairs are inelastic and characterized by independent coefficients of normal restitution $\alpha_{ij}=\alpha_{ji}$, where $\alpha_{ij}$ is the coefficient of restitution for collisions between particles of species *i* and *j*. For moderately dense systems, in the presence of the gravity field $m_{i}g$, the set of Enskog kinetic equations is

(92) $$\frac{\partial }{\partial t}f_{i}+v\cdot \nabla f_{i}+g\cdot \frac{\partial f_{i}}{\partial v}=\sum_{j=1}^{s}\;J_{E,ij}\left[r,v|f_{i},f_{j}\right],$$

where the Enskog collision operators $J_{E,ij}$ for collisions *i*-*j* in the Δ-model read [93]

(93) $$\begin{array}{ccc} & & J_{E,\mathrm{ij}}\left[r,v_{1}|f_{i},f_{j}\right]\equiv \sigma_{ij}^{d-1}\int dv_{2}\int d\hat{\sigma }\Theta \left(-\hat{\sigma }\cdot g_{12}-2\Delta_{ij}\right)\left(-\hat{\sigma }\cdot g_{12}-2\Delta_{ij}\right)\\ & & \times \alpha_{ij}^{-2}f_{2,ij}\left(r,r+\sigma_{ij},v_{1}^{\prime \prime },v_{2}^{\prime \prime };t\right)-\sigma_{ij}^{d-1}\int \;dv_{2}\int d\hat{\sigma }\Theta \left(\hat{\sigma }\cdot g_{12}\right)\left(\hat{\sigma }\cdot g_{12}\right)\\ & & \times f_{2,ij}\left(r,r+\sigma_{ij},v_{1},v_{2};t\right). \end{array}$$

Here, $\sigma_{ij}=\sigma_{ij}\hat{\sigma }$, $\sigma_{ij}=\left(\sigma_{i}+\sigma_{j}\right)/2$, and

(94) $$f_{2,ij}\left(r_{1},r_{2},v_{1},v_{2};t\right)\equiv \chi_{ij}\left(r_{1},r_{2}\right)f_{i}\left(r_{1},v_{1};t\right)f_{j}\left(r_{2},v_{2};t\right),$$

$\chi_{ij}\left(r_{1},r_{2}\right)$ being the pair correlation function for collisions i−j. In Equation (93), the collision rules for the restituting collisions $\left(v_{1}^{\prime \prime },v_{2}^{\prime \prime }\right)\to \left(v_{1},v_{2}\right)$ with the same collision vector $\hat{\sigma }$ are defined as

(95) $$v_{1}^{\prime \prime }=v_{1}-\mu_{ji}\left(1+\alpha_{ij}^{-1}\right)\left(\hat{\sigma }\cdot g_{12}\right)\hat{\sigma }-2\mu_{ji}\Delta_{ij}\alpha_{ij}^{-1}\hat{\sigma },$$

(96) $$v_{2}^{\prime \prime }=v_{2}+\mu_{ij}\left(1+\alpha_{ij}^{-1}\right)\left(\hat{\sigma }\cdot g_{12}\right)\hat{\sigma }+2\mu_{ij}\Delta_{ij}\alpha_{ij}^{-1}\hat{\sigma },$$

where $\mu_{ij}=m_{i}/\left(m_{i}+m_{j}\right)$. Analogously, the direct collisions $\left(v_{1},v_{2}\right)\to \left(v_{1}^{\prime },v_{2}^{\prime }\right)$ are defined as

(97) $$v_{1}^{\prime }=v_{1}-\mu_{ji}\left(1+\alpha_{ij}\right)\left(\hat{\sigma }\cdot g_{12}\right)\hat{\sigma }-2\mu_{ji}\Delta_{ij}\hat{\sigma },$$

(98) $$v_{2}^{\prime }=v_{2}+\mu_{ij}\left(1+\alpha_{ij}\right)\left(\hat{\sigma }\cdot g_{12}\right)\hat{\sigma }+2\mu_{ij}\Delta_{ij}\hat{\sigma }.$$

As in the case of monocomponent granular fluids, the property (14) for the Enskog collision operators $J_{E,ij}\left[r,v|f_{i},f_{j}\right]$ still applies except that $f_{2}\left(r,r+\sigma ,v_{1},v_{2};t\right)$ must be replaced by $f_{2,ij}\left(r,r+\sigma_{ij},v_{1},v_{2};t\right)$ and $v_{1}^{\prime }$ is given by Equation (97).

The use of property (14) for granular mixtures allows deriving the corresponding balance equations for the densities of mass, momentum, and energy. As expected, their forms are similar to those obtained in the IHS model [141] and are given by

(99) $$D_{t}n_{i}+n_{i}\nabla \cdot U+\frac{\nabla \cdot j_{i}}{m_{i}}=0,$$

(100) $$D_{t}U+\rho^{-1}\nabla \cdot P=g,$$

(101) $$D_{t}T-\frac{T}{n}\sum_{i=1}^{s}\frac{\nabla \cdot j_{i}}{m_{i}}+\frac{2}{dn}\left(\nabla \cdot q+P:\nabla U\right)=-\zeta \;T.$$

In Equations (99)–(101),

(102) $$n_{i}=\int dvf_{i}\left(v\right)$$

is the number density of species *i*,

(103) $$U=\rho^{-1}\sum_{i=1}^{s}m_{i}\int dvvf_{i}\left(v\right)$$

is the mean flow velocity, and

(104) $$T=\frac{1}{dn}\sum_{i=1}^{s}m_{i}\int dvV^{2}f_{i}\left(v\right)$$

is the (global) granular temperature. In addition, $\rho =\sum_{i}\rho_{i}=\sum_{i}m_{i}n_{i}$ is the total mass density, and we recall that V=v−U is the peculiar velocity. Apart from the granular temperature *T*, at a kinetic level it is convenient to introduce the partial temperatures $T_{i}$ for each species; they measure their mean kinetic energies. They are defined as

(105) $$n_{i}T_{i}=\frac{m_{i}}{d}\int dvV^{2}f_{i}\left(v\right),$$

and, hence, $nT=\sum_{i}n_{i}T_{i}$.

In the balance Equations (99)–(101),

(106) $$j_{i}=m_{i}\int dv_{1}\;V_{1}\;f_{i}\left(v_{1}\right)$$

is the mass flux for the species *i* relative to the local flow. The mass flux $j_{i}$ has only *kinetic* contributions. The kinetic contributions to the pressure tensor P and the heat flux q are given as usual by

(107) $$P_{k}=\sum_{i=1}^{s}\;\int dv\;m_{i}VV\;f_{i}\left(v\right),$$

(108) $$q_{k}=\sum_{i=1}^{s}\;\int dv\;\frac{1}{2}m_{i}V^{2}V\;f_{i}\left(v\right).$$

The collisional transfer contributions for the pressure tensor and the heat flux can be derived by following similar steps to those made in the Δ-model for monocomponent granular gases [84]. Their expressions are

(109) $$\begin{array}{ccc} P_{c} & = & \sum_{i=1}^{s}\sum_{j=1}^{s}\frac{1+\alpha_{ij}}{2}m_{ij}\sigma_{ij}^{d}\int dv_{1}\int dv_{2}\int d\hat{\sigma }\;\Theta \left(\hat{\sigma }\cdot g_{12}\right)\left(\hat{\sigma }\cdot g_{12}\right)\hat{\sigma }\hat{\sigma }\\ & & \times \left[\left(\hat{\sigma }\cdot g_{12}\right)+\frac{2\Delta_{ij}}{1+\alpha_{ij}}\right]\int_{0}^{1}\;d\lambda f_{2,ij}\left(r-\lambda \sigma_{ij},r+\left(1-\lambda \right)\sigma_{ij},v_{1},v_{2},t\right), \end{array}$$

(110) $$\begin{array}{ccc} q_{c} & = & \sum_{i=1}^{s}\sum_{j=1}^{s}\frac{1+\alpha_{ij}}{8}m_{ij}\sigma_{ij}^{d}\int dv_{1}\int dv_{2}\int d\hat{\sigma }\Theta \left(\hat{\sigma }\cdot g_{12}\right){\left(\hat{\sigma }\cdot g_{12}\right)}^{2}\hat{\sigma }[4\left(\hat{\sigma }\cdot G_{ij}\right)\\ & & +\left(\mu_{ji}-\mu_{ij}\right)\left(1-\alpha_{ij}\right)\left(\hat{\sigma }\cdot g_{12}\right)]\int_{0}^{1}d\lambda f_{2,ij}\left(r-\lambda \sigma_{ij},r+\left(1-\lambda \right)\sigma_{ij},v_{1},v_{2},t\right)\\ & & -\sum_{i=1}^{s}\sum_{j=1}^{s}\frac{m_{i}}{4}\sigma_{ij}^{d}\Delta_{ij}\int dv_{1}\int dv_{2}\int d\hat{\sigma }\Theta \left(\hat{\sigma }\cdot g_{12}\right)\left(\hat{\sigma }\cdot g_{12}\right)\hat{\sigma }[4\mu_{ji}^{2}\Delta_{ij}\\ & & +4\mu_{ji}^{2}\alpha_{ij}\left(\hat{\sigma }\cdot g_{12}\right)-4\mu_{ji}\left(\hat{\sigma }\cdot G_{ij}\right)]\int_{0}^{1}d\lambda f_{2,ij}\left(r-\lambda \sigma_{ij},r+\left(1-\lambda \right)\sigma_{ij},v_{1},v_{2},t\right). \end{array}$$

The energy rate ζ is

(111) $$\begin{array}{ccc} \zeta & = & -\frac{2}{dnT}\sum_{i=1}^{s}\sum_{j=1}^{s}\sigma_{ij}^{d-1}m_{ij}\int dv_{1}\int dv_{2}\int d\hat{\sigma }\;\Theta \left(\hat{\sigma }\cdot g_{12}\right)\left(\hat{\sigma }\cdot g_{12}\right)\\ & & \times \left[\Delta_{ij}^{2}+\alpha_{ij}\Delta_{ij}\left(\hat{\sigma }\cdot g_{12}\right)-\frac{1-\alpha_{ij}^{2}}{4}{\left(\hat{\sigma }\cdot g_{12}\right)}^{2}\right]f_{2,ij}\left(r,r+\sigma_{ij},v_{1},v_{2},t\right). \end{array}$$

In Equations (110) and (111), $m_{ij}=m_{i}m_{j}/\left(m_{i}+m_{j}\right)$ is the reduced mass and $G_{ij}=\mu_{ij}V_{1}+\mu_{ji}V_{2}$ is the center-of-mass velocity.

### 5.2. Homogeneous Time-Dependent State

As in the case of monocomponent gases, we consider first spatially homogeneous isotropic states for which the Enskog Equation (92) in the absence of gravity becomes

(112) $$\frac{\partial }{\partial t}f_{i}\left(v;t\right)=\sum_{j=1}^{s}\;J_{E,ij}\left[v|f_{i},f_{j}\right],$$

where $J_{E,ij}\left[f_{i},f_{j}\right]$ is defined by Equation (93) with the replacements $\chi_{ij}\left(r,r\pm \sigma_{ij}\right)\to \chi_{ij}$ and $f_{2,ij}\left(r,r+\sigma_{ij},v_{1},v_{2};t\right)\to \chi_{ij}f_{i}\left(v_{1};t\right)f_{j}\left(v_{2};t\right)$. Here, $\chi_{ij}$ is the (homogeneous) pair correlation function at contact for collisions *i*-*j*.

In the homogeneous time-dependent state, the only nontrivial balance equation is that for the temperature *T*. Since the mass and heat fluxes vanish and U=0, the equation for T(t) of a multicomponent granular mixture is still given by Equation (28) with

(113) $$\begin{array}{ccc} \zeta & = & -\frac{2}{dnT}\sum_{i=1}^{s}\sum_{j=1}^{s}\sigma_{ij}^{d-1}m_{ij}\chi_{ij}\int dv_{1}\int dv_{2}\left(B_{1}g_{12}\Delta_{ij}^{2}+B_{2}g_{12}^{2}\alpha_{ij}\Delta_{ij}-B_{3}g_{12}^{3}\frac{1-\alpha_{ij}^{2}}{4}\right)\\ & & \times f_{i}\left(v_{1},t\right)f_{j}\left(v_{2},t\right), \end{array}$$

where the coefficients $B_{k}$ are defined in Equation (30). The time evolution of the partial temperatures $T_{i}$ can be directly obtained from the Enskog Equation (112) and the definition (105):

(114) $$\frac{\partial T_{i}}{\partial t}=-\zeta_{i}T_{i},$$

where

(115) $$\zeta_{i}=\sum_{j=1}^{s}\zeta_{ij}=-\frac{1}{dn_{i}T_{i}}\sum_{j=1}^{s}\int dvm_{i}v^{2}J_{E,ij}\left[f_{i},f_{j}\right].$$

According to Equations (114) and (115),

(116) $$\zeta =\sum_{i=1}^{s}\;x_{i}\gamma_{i}\zeta_{i},$$

where $x_{i}=n_{i}/n$ is the concentration or mole fraction of species *i* and $\gamma_{i}=T_{i}/T$ is the temperature ratio of species *i*. The deviation of $\gamma_{i}$ from 1 provides a measure of the departure from energy equipartition (i.e., when $T_{i}=T$ for any component *i*). The time evolution of the temperature ratios $\gamma_{i}\left(t\right)$ can be easily obtained from Equations (28) and (114) as

(117) $$\frac{\partial }{\partial t}\ln\gamma_{i}=\zeta -\zeta_{i}.$$

As in the monocomponent case, after a transient period, one expects that the velocity distribution functions $f_{i}\left(v,t\right)$ adopt a *normal* form where their time dependence is only through the global granular temperature T(t). This means that $f_{i}\left(v,t\right)$ is given by the scaling distribution

(118) $$f_{i}\left(v,t\right)=n_{i}v_{\mathrm{th}}^{-d}\left(t\right)\varphi_{i}\left(c,\Delta_{\ell j}^{*}\right),\;\ell ,j=1,\ldots ,s,$$

where we recall that $c\equiv v/v_{\mathrm{th}}$ and $v_{\mathrm{th}}\left(t\right)=\sqrt{2T\left(t\right)/\bar{m}}$ is a thermal velocity defined in terms of the temperature of the mixture T(t). In addition, $\bar{m}=\sum_{i}m_{i}/s$ and $\Delta_{ij}^{*}\left(t\right)\equiv \Delta_{ij}/v_{\mathrm{th}}\left(t\right)$. According to Equation (118), $\partial_{t}f_{i}=-\zeta T\partial_{T}f_{i}$ and, so,

(119) $$\frac{\partial f_{i}}{\partial t}=\frac{1}{2}\zeta \frac{\partial }{\partial v}\cdot \left(vf_{i}\right)+\frac{1}{2}\zeta \sum_{j=1}^{s}\sum_{\ell =1}^{s}\;\Delta_{\ell j}^{*}\frac{\partial f_{i}}{\partial \Delta_{\ell j}^{*}}.$$

Thus, in dimensionless form, the set of *s*-coupled Enskog Equation (112) for the homogeneous time-dependent problem can be written as

(120) $$\frac{1}{2}\zeta^{*}\left(\frac{\partial }{\partial c}\cdot \left(c\varphi_{i}\right)+\sum_{j=1}^{s}\sum_{\ell =1}^{s}\;\Delta_{\ell j}^{*}\frac{\partial \varphi_{i}}{\partial \Delta_{\ell j}^{*}}\right)=\sum_{j=1}^{s}\;J_{E,ij}^{*}\left[c|\varphi_{i},\varphi_{j}\right],$$

where $\zeta^{*}=\zeta /\nu$ and $J_{E,ij}^{*}\left[c|\varphi_{i},\varphi_{j}\right]=\left(v_{\mathrm{th}}^{d}/n_{i}\nu \right)J_{E,ij}\left[v|f_{i},f_{j}\right]$. Here, $\nu =n{\bar{\sigma }}^{d-1}v_{\mathrm{th}}$ is an effective collision frequency and $\bar{\sigma }=\sum_{i}\sigma_{i}/s$.

Since $f_{i}\left(v\right)$ depends on v through its modulus, the mass and heat fluxes vanish and $P_{\lambda \beta }=p\delta_{\lambda \beta }$. The hydrostatic pressure $p=nTp^{*}$, where the coefficient $p^{*}$ for a multicomponent granular mixture is

(121) $$\begin{array}{ccc} p^{*} & = & 1+\frac{\pi^{d/2}}{d\Gamma \left(\frac{d}{2}\right)}\sum_{i=1}^{s}\sum_{j=1}^{s}\mu_{ji}n\sigma_{ij}^{d}\chi_{ij}x_{i}x_{j}[\left(1+\alpha_{ij}\right)\gamma_{i}+\frac{2}{\sqrt{\pi }}\frac{\Gamma \left(\frac{d}{2}\right)}{\Gamma \left(\frac{d+1}{2}\right)}\frac{m_{i}}{\bar{m}}\Delta_{ij}^{*}\\ & & \times \int dc_{1}\int dc_{2}g_{12}^{*}\varphi_{i}\left(c_{1}\right)\varphi_{j}\left(c_{2}\right)]. \end{array}$$

For mechanically equivalent particles ($m_{i}=m$, $\sigma_{i}=\sigma$, $\alpha_{ij}=\alpha$, and $\Delta_{ij}^{*}=\Delta^{*}$), $\gamma_{i}=\theta_{i}=1$ and Equation (121) agrees with Equation (37).

### 5.3. Homogeneous Steady States: Maxwellian Approximation

We consider here the steady state solution to Equation (120). In this case, for given values of $\Delta_{ij}^{*}$, $\partial_{t}T_{i}\left(t\right)=0$ and according to Equation (114)

(122) $$\zeta =\zeta_{1}=\zeta_{2}=\ldots =\zeta_{s}=0.$$

As expected, the determination of $\zeta_{i}$ requires the knowledge of the scaling distributions $\varphi_{i}$, whose exact form is not known to date. As in the conventional IHS model [86], the distributions $\varphi_{i}$ can be expanded in a series of Sonine polynomials, the coefficients (cumulants) of the series being the corresponding velocity moments of $\varphi_{i}$. Here, as in Section 3, to estimate the partial energy rates $\zeta_{i}$, we take the simplest Maxwellian approximation $\varphi_{i,M}\left(c\right)$ to $\varphi_{i}\left(c\right)$, namely,

(123) $$\varphi_{i}\left(c\right)\to \varphi_{i,M}\left(c\right)=\pi^{-d/2}\theta_{i}^{d/2}\;e^{-\theta_{i}c^{2}},$$

where $\theta_{i}=m_{i}/\left(\bar{m}\gamma_{i}\right)$. As in previous works on granular mixtures [86], for the sake of convenience, $\varphi_{i,M}$ is defined in terms of the partial temperature $T_{i}$ instead of the (global) granular temperature *T*.

With the Maxwellian approximation (123), the partial energy rate $\zeta_{i}\to \zeta_{i,M}$ can be computed. In dimensionless form, it can be written as $\zeta_{i,M}=\zeta_{i,M}^{*}\nu$ where [93]

(124) $$\begin{array}{ccc} \zeta_{i,M}^{*} & = & \frac{4\pi^{(d-1)/2}}{d\Gamma \left(\frac{d}{2}\right)}\sum_{j=1}^{s}x_{j}\chi_{ij}{\left(\frac{\sigma_{ij}}{\bar{\sigma }}\right)}^{d-1}\mu_{ji}\left(1+\alpha_{ij}\right)\theta_{i}^{-1/2}{\left(1+\theta_{ij}\right)}^{1/2}\\ & & \times \left[1-\frac{1}{2}\mu_{ji}\left(1+\alpha_{ij}\right)\left(1+\theta_{ij}\right)\right]-\frac{4\pi^{d/2}}{d\Gamma \left(\frac{d}{2}\right)}\sum_{j=1}^{s}x_{j}\chi_{ij}{\left(\frac{\sigma_{ij}}{\bar{\sigma }}\right)}^{d-1}\mu_{ji}\Delta_{ij}^{*}\\ & & \times \left[\frac{2\mu_{ji}\Delta_{ij}^{*}}{\sqrt{\pi }}\theta_{i}^{1/2}{\left(1+\theta_{ij}\right)}^{1/2}-1+\mu_{ji}\left(1+\alpha_{ij}\right)\left(1+\theta_{ij}\right)\right]. \end{array}$$

Here, $\theta_{ij}=\theta_{i}/\theta_{j}=m_{i}\gamma_{j}/m_{j}\gamma_{i}$ gives the ratio between the mean-square velocity of the particles of the species *j* relative to that of the particles of the species *i*. Moreover, taking the Maxwellian approximation (123), the expression (121) for $p^{*}$ reduces to

(125) $$p_{M}^{*}=1+\frac{\pi^{d/2}}{d\Gamma \left(\frac{d}{2}\right)}\sum_{i=1}^{s}\sum_{j=1}^{s}\mu_{ji}n\sigma_{ij}^{d}\chi_{ij}x_{i}x_{j}\left[\left(1+\alpha_{ij}\right)\gamma_{i}+\frac{2}{\sqrt{\pi }}\frac{m_{i}}{\bar{m}}\Delta_{ij}^{*}{\left(\frac{\theta_{i}+\theta_{j}}{\theta_{i}\theta_{j}}\right)}^{1/2}\right].$$

In the limit of mechanically equivalent particles, Equations (124) and (125) agree with Equations (41) and (42), respectively, as expected.

### 5.4. Binary Mixtures: Comparison Between Kinetic Theory and Computer Simulations

To illustrate the dependence of the partial temperatures on the parameters of the mixture, we consider a binary mixture (s=2) for the sake of simplicity. In this case, the relevant dimensionless quantities in the steady state are the scaled temperature $T^{*}$ (defined below) and the temperature ratio $T_{1}/T_{2}$. Both quantities are determined from the following constraints (122):

(126) $$\zeta_{1}^{*}=0,\;\zeta_{2}^{*}=0.$$

The solution to Equation (126) with the expression (124) for the energy rates provides $T^{*}$ and $T_{1}/T_{2}$ in terms of the parameter space of the problem. This is constituted of the ratio of masses $m_{1}/m_{2}$, the ratio of diameters $\sigma_{1}/\sigma_{2}$, the concentration $x_{1}$, the volume fraction or density ϕ, the coefficients of restitution $\alpha_{11}$, $\alpha_{22}$, and $\alpha_{12}$, and the dimensionless velocities $\Delta_{11}^{*}$, $\Delta_{22}^{*}$, and $\Delta_{12}^{*}$. In the case of a two-dimensional (d=2) system, the volume fraction ϕ is defined as

(127) $$\phi =\sum_{i=1}^{2}\;\frac{\pi }{4}n_{i}\sigma_{i}^{2},$$

while a good approximation for the pair distribution function is [85]

(128) $$\chi_{ij}=\frac{1}{1-\phi }+\frac{9}{16}\frac{\phi }{{\left(1-\phi \right)}^{2}}\frac{\sigma_{i}\sigma_{j}M_{1}}{\sigma_{ij}M_{2}},$$

where $M_{\ell }=\sum_{i}x_{i}\sigma_{i}^{\ell }$. Finally, the reduced (steady) temperature $T^{*}$ is defined as

(129) $$T^{*}=\frac{T}{\bar{m}{\bar{\Delta }}^{2}/2},$$

where $\bar{\Delta }=\sqrt{\Delta_{11}^{2}+\Delta_{22}^{2}+\Delta_{12}^{2}}.$

Since there are relatively many parameters involved in the problem, we take a common coefficient of restitution $\alpha_{11}=\alpha_{22}=\alpha_{12}\equiv \alpha$, as usual. Moreover, we consider two-dimensional granular mixtures with the concentration $x_{1}=\frac{1}{2}$. The (approximate) theoretical results for $T^{*}$ and $T_{1}/T_{2}$ will be compared with two different standard simulation methods. The first one is the direct simulation Monte Carlo (DSMC) method [114] introduced years ago by Bird for dilute molecular gases. Here, we have adapted this method to a gas of hard disks with inelastic collisions. The DSMC method numerically solves the inelastic Boltzmann equation and assumes the molecular chaos hypothesis, namely the absence of velocity correlations between particles about to collide. However, the method does not assume the existence of the normal solution (118) and it goes beyond the Maxwellian approximation to $f_{i}$ (it determines the “exact” velocity distribution functions). Thus, comparing analytical and DSMC results for very dilute systems (ϕ→0) can be used to evaluate the reliability of the scaling solution (118) and the accuracy of the expression (124) for estimating the partial energy rates (this expression is obtained by replacing the true $\varphi_{i}$ by its Maxwellian form (123)). As previously noted in Section 4, since MD simulations avoids the assumptions of the Enskog kinetic theory, comparing the theory to MD is more stringent than comparing it to DSMC results. In both simulation methods (DSMC and MD), particles move in two dimensions with collisions given by rules (97) and (98) of the Δ-model.

**Figure 6:**
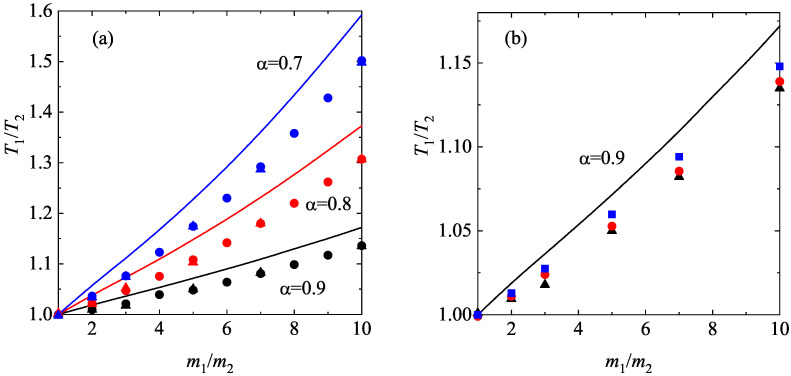
Panel (**a**): Plot of the temperature ratio $T_{1}/T_{2}$ versus the mass ratio $m_{1}/m_{2}$ for $\sigma_{1}=\sigma_{2}$, and three different values of the (common) coefficient of restitution α: α=0.9, 0.8 and 0.7. The lines refer to the Enskog theoretical results while the symbols correspond to the results obtained by numerically solving the Enskog equation by means of the DSMC method (circles) and by performing MD simulations for ϕ=0.0016 (triangles). Panel (**b**): Plot of the temperature ratio $T_{1}/T_{2}$ versus the mass ratio $m_{1}/m_{2}$ for $\sigma_{1}=\sigma_{2}$, α=0.7, and three different values of the volume fraction ϕ: ϕ=0.0016 (triangles), 0.1 (circles) and 0.2 (squares). Symbols refer to MD simulations and the line to the Enskog theoretical result. We assume in both panels that $\Delta_{11}=\Delta_{22}=\Delta_{12}$. Figure reprinted with permission from R. Brito, R. Soto, and V. Garzó, Phys. Rev. E **2020**, *102*, 052904 [93]. Copyright (2020) by the American Physical Society.

We consider first the usual case of binary mixtures where their constituents differ only by their diameters and masses but the energy injection parameters are the same for all types of collisions (i.e., $\Delta_{11}=\Delta_{22}=\Delta_{12}$). To assess the departure from energy equipartition, panels (a) and (b) of Figure 6 show the temperature ratio $T_{1}/T_{2}$ as a function of the mass ratio. In panel (a), we consider different values of the (common) coefficient of restitution α. As occurs in the conventional IHS model [38,86,120,122,142], $T_{1}/T_{2}$ increases with increasing mass ratio $m_{1}/m_{2}$ and, hence, the temperature of the heavier particles is larger than that of the lighter ones. In any case, the departure from energy equipartition is less significant in the Delta-collisional model than in the IHS model. While both simulation methods agree again with great accuracy, they deviate from the theoretical results as the mass ratio grows. As is well known, in the case of elastic collisions [110], the use of the simplest leading-order truncation to evaluate the transport coefficients is accurate to approximately 5%. However, there are exceptions, such as extreme mass ratios (e.g., electron–proton systems). For inelastic collisions, the discrepancy between kinetic theory and simulations for disparate-mass binary mixtures may also originate from the use of the Maxwellian approximation (123) to $\varphi_{i}$ (leading-order truncation) to determine the temperature ratio. Apart from this source of discrepancy, one could argue that molecular chaos is more likely broken in highly asymmetric mixtures.

To gauge the impact of density on $T_{1}/T_{2}$, we plot it versus $m_{1}/m_{2}$ for three different values of the solid volume fraction ϕ in panel (b) of Figure 6. Since $\sigma_{1}=\sigma_{2}$ and $x_{1}=\frac{1}{2}$, Equation (128) yields $\chi_{11}=\chi_{22}=\chi_{12}$ and, hence, they factor in Equation (126). Consequently, the Enskog kinetic theory does not predict any dependence of $T^{*}$ and $T_{1}/T_{2}$ on the density ϕ. However, beyond the Enskog equation, density corrections to $T_{1}/T_{2}$ can exist if there are position correlations not accounted for in the approximation (128) for $\chi_{ij}$. The comparison with MD simulations carried in panel (b) tests this prediction. We observe from panel (b) of Figure 6 that the dependence of $T_{1}/T_{2}$ on ϕ is very weak (mostly appears at high mass ratio), validating the results derived from the Enskog equation.

**Figure 7:**
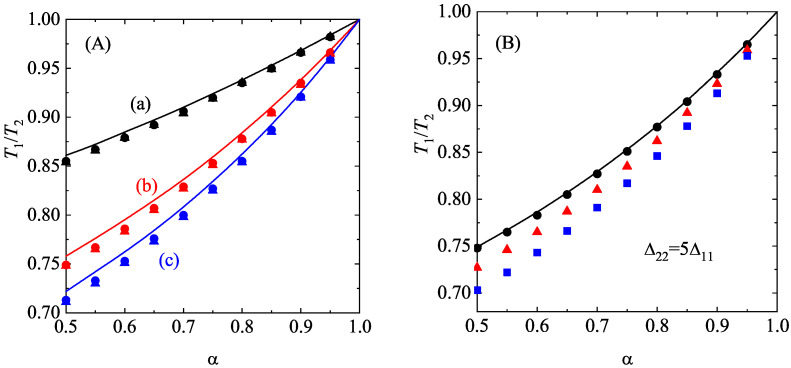
Panel (**A**): Plot of the temperature ratio $T_{1}/T_{2}$ versus the (common) coefficient of restitution α for $\sigma_{1}=\sigma_{2}$ and $m_{1}=m_{2}$. We assume here that $\Delta_{22}=\lambda \Delta_{11}$ and $\Delta_{12}=\left(\Delta_{11}+\Delta_{22}\right)/2$. Three different values of λ have been considered: λ=2 (a), λ=5 (b), and λ=10 (c). Symbols refer to DSMC results (circles) and MD simulations (triangles) for ϕ=0.01 while the lines correspond to the Enskog theoretical results. Panel (**B**): Plot of the temperature ratio $T_{1}/T_{2}$ versus the (common) coefficient of restitution α for for $\sigma_{1}=\sigma_{2}$ and $m_{1}=m_{2}$. We assume here that λ=5 and, so, $\Delta_{22}=5\Delta_{11}$ and $\Delta_{12}=3\Delta_{11}$. Three different values of the solid volume fraction are considered: ϕ=0.01 (solid line and circles), ϕ=0.1 (triangles), and ϕ=0.2 (squares). Symbols refer to MD simulations and the line to the Enskog theoretical result. Figure reprinted with permission from R. Brito, R. Soto, and V. Garzó, Phys. Rev. E **2020**, *102*, 052904 [93]. Copyright (2020) by the American Physical Society.

Now, we consider the case in which the only difference between the two species is the energy injection at collisions. Namely, the two species are mechanically equivalent ($\sigma_{1}=\sigma_{2}$ and $m_{1}=m_{2}$), but $\Delta_{11}\neq \Delta_{22}\neq \Delta_{12}$. Specifically, we assume that $\Delta_{11}<\Delta_{22}$ and $\Delta_{12}=\left(\Delta_{11}+\Delta_{22}\right)/2$. Since $\Delta_{11}<\Delta_{22}$, $\Delta_{12}>\Delta_{11}$. This means that the particles of species 1 (2) have a higher (lower) temperature than if they were alone because the energy injected in the 1-2 collisions is higher (lower) than in the 1-1 (2-2) collisions. Figure 7 illustrates the α-dependence of the temperature ratio for different systems and densities. In general, we observe that the Enskog theoretical predictions agree with the DSMC and MD simulations at low density. Panel (A) of Figure 7 highlights a significant departure from energy equipartition, as $T_{1}/T_{2}$ differs greatly from 1. Clearly, the energy injection for species 1 is smaller than for species 2, as evidenced by the fact that the temperature ratio $T_{1}/T_{2}<1$. The effect of density on $T_{1}/T_{2}$ is illustrated in panel (B). We observe that the influence of ϕ on $T_{1}/T_{2}$ is more pronounced in this case than in the scenario depicted in Panel (b) of Figure 6.

In summary, the comparison carried out in this section between kinetic theory and computer simulations shows that the failure of the Enskog kinetic theory at high densities can be expected based on previous results obtained for ordinary (elastic) mixtures [110]. At high densities, effects such as multiparticle collisions are not accounted for in the Enskog collision operator. These effects are expected to be more pronounced in granular fluids than in the conventional fluids since the colliding pairs tend to be more focused. Consequently, the range of densities for which the Enskog theory is expected to provide accurate results diminishes with increasing collisional dissipation.

## 6. Navier–Stokes Transport Coefficients for Binary Granular Mixtures: Low-Density Regime

### 6.1. Kinetic and Balance Equations

As in the monocomponent gas case, once the homogeneous time-dependent state for multicomponent systems is characterized, the next step is to use this state as the reference state in the Chapman–Enskog perturbation solution of the Enskog kinetic Equation (92). However, studying the transport properties of multicomponent granular mixtures is much more complicated than studying those of a single granular gas. This is because the number of transport coefficients in a mixture is larger than in a monocomponent gas, and these coefficients depend on parameters such as diameters, masses, concentration, and coefficients of restitution. Due to these difficulties, our analysis in this section is restricted to the *low-density* regime of a granular binary mixture (s=2). In this regime, $\chi_{ij}=1$ and the Enskog collision operators $J_{E,ij}\left[f_{i},f_{j}\right]$ reduce to the Boltzmann operators

(130) $$\begin{array}{c} J_{ij}\left[v_{1}|f_{i},f_{j}\right]\equiv \sigma_{ij}^{d-1}\int dv_{2}\int d\hat{\sigma }\Theta \left(-\hat{\sigma }\cdot g_{12}-2\Delta_{ij}\right)\left(-\hat{\sigma }\cdot g_{12}-2\Delta_{ij}\right)\\ \times \alpha_{ij}^{-2}f_{i}\left(r,v_{1}^{\prime \prime },t\right)f_{j}\left(r,v_{2}^{\prime \prime },t\right)-\sigma_{ij}^{d-1}\int \;dv_{2}\int d\hat{\sigma }\Theta \left(\hat{\sigma }\cdot g_{12}\right)\left(\hat{\sigma }\cdot g_{12}\right)f_{i}\left(r,v_{1},t\right)f_{j}\left(r,v_{2},t\right). \end{array}$$

Moreover, in the low-density regime, the collisional contributions to the transport coefficients are much smaller than their kinetic forms and, hence, they can be neglected.

The determination of the Chapman–Enskog solution to first order in spatial gradients in the Δ-model follows similar mathematical steps to those made in the conventional IHS model [51,141,143,144]. One subtle point in implementing the Chapman–Enskog method in the Δ-model for mixtures is that there are nonzero first-order contributions to the partial temperatures and the energy rate. Most of the technical details involved in this derivation can be found in Ref. [145].

As discussed in Section 4, we assume that after a transient regime the granular mixture achieves a hydrodynamic state characterized by the fact that the distributions $f_{i}\left(r,v,t\right)$ depend on space and time through a *functional* dependence on the hydrodynamic fields (*normal* solution). Here, as in the case of the IHS model [143], we take the concentration $x_{1}$, the pressure p=nT, the temperature *T*, and the mean flow velocity U as the hydrodynamic fields of the binary mixture. For small spatial gradients, $f_{i}$ can be written as a series expansion in powers of the nonuniformity parameter ϵ,

(131) $$f_{i}=f_{i}^{\left(0\right)}+\epsilon \;f_{i}^{\left(1\right)}+\epsilon^{2}\;f_{i}^{\left(2\right)}+\ldots \;,$$

where the formal parameter ϵ is taken to be equal to 1 at the end of the calculations. As expected, the zeroth-order distribution $f_{i}^{\left(0\right)}$ is nothing more than the local version of the homogeneous time-dependent distribution (118) studied in Section 5.2.

The balance hydrodynamic equations to first order are

(132) $$D_{t}^{\left(1\right)}x_{1}=0,\;D_{t}^{\left(1\right)}U_{\lambda }=-\rho^{-1}\nabla_{\lambda }p+g_{\lambda },$$

(133) $$D_{t}^{\left(1\right)}p=-\frac{d+2}{d}p\nabla \cdot U-p\zeta^{\left(1\right)},\;D_{t}^{\left(1\right)}T=-\frac{2}{d}T\nabla \cdot U-T\zeta^{\left(1\right)},$$

where $D_{t}^{\left(1\right)}=\partial_{t}^{\left(1\right)}+U\cdot \nabla$ and $\zeta^{\left(1\right)}=\zeta_{U}\nabla \cdot U$ is the first-order contribution to the energy rate. The kinetic equation verifying the first-order distribution $f_{i}^{\left(1\right)}\left(r,v,t\right)$ can be obtained by employing the balance Equations (132) and (133). The solution to this kinetic equation is given by [145]

(134) $$\begin{array}{ccc} f_{i}^{\left(1\right)}\left(V\right) & = & A_{i}\left(V\right)\cdot \nabla x_{1}+B_{i}\left(V\right)\cdot \nabla p+C_{i}\left(V\right)\cdot \nabla T\\ & & +D_{i,\lambda \beta }\left(V\right)\frac{1}{2}\left(\nabla_{\lambda }U_{\beta }+\nabla_{\beta }U_{\lambda }-\frac{2}{d}\delta_{\lambda \beta }\nabla \cdot U\right)+E_{i}\left(V\right)\nabla \cdot U\;. \end{array}$$

In Equation (134), the quantities $A_{i}\left(V\right)$, $B_{i}\left(V\right)$, $C_{i}\left(V\right)$, $D_{i,\beta \lambda }\left(V\right)$, and $E_{i}\left(V\right)$ obey the following linear set of coupled integral equations:

(135) $$\left[-\zeta^{\left(0\right)}\left(T\partial_{T}+p\partial_{p}\right)+L_{i}\right]A_{i}+M_{i}A_{j}=A_{i}+{\left(\frac{\partial \zeta^{\left(0\right)}}{\partial x_{1}}\right)}_{p,T}\left(pB_{i}+TC_{i}\right),$$

(136) $$\left[-\zeta^{\left(0\right)}\left(T\partial_{T}+p\partial_{p}\right)+L_{i}-2\zeta^{\left(0\right)}\right]B_{i}+M_{i}B_{j}=B_{i}+\frac{T\zeta^{\left(0\right)}}{p}C_{i},$$

(137) $$\begin{array}{ccc} & & \left[-\zeta^{\left(0\right)}\left(T\partial_{T}+p\partial_{p}\right)+L_{i}-\frac{1}{2}\zeta^{\left(0\right)}\left(1-\Delta^{*}\frac{\partial \ln\zeta_{0}^{*}}{\partial \Delta^{*}}\right)\right]C_{i}+M_{i}C_{j}=C_{i}\\ & & -\frac{p\zeta^{\left(0\right)}}{2T}\left(1+\Delta^{*}\frac{\partial \ln\zeta_{0}^{*}}{\partial \Delta^{*}}\right)B_{i}, \end{array}$$

(138) $$\left[-\zeta^{\left(0\right)}\left(T\partial_{T}+p\partial_{p}\right)+L_{i}\right]D_{i,\beta \lambda }+M_{i}D_{j,\beta \lambda }=D_{i,\beta \lambda },$$

(139) $$\left[-\zeta^{\left(0\right)}\left(T\partial_{T}+p\partial_{p}\right)+L_{i}\right]E_{i}+M_{i}E_{j}=E_{i},$$

where $\zeta_{0}^{*}=\zeta^{\left(0\right)}/\nu$ and we have introduced the linearized Boltzmann operators

(140) $$L_{i}f_{i}^{\left(1\right)}=-\left(J_{ii}\left[f_{i}^{\left(0\right)},f_{i}^{\left(1\right)}\right]+J_{ii}\left[f_{i}^{\left(1\right)},f_{i}^{\left(0\right)}\right]+J_{ij}\left[f_{i}^{\left(1\right)},f_{j}^{\left(0\right)}\right]\right),$$

(141) $$M_{i}f_{j}^{\left(1\right)}=-J_{ij}\left[f_{i}^{\left(0\right)},f_{j}^{\left(1\right)}\right].$$

In Equations (135)–(141), the index *j* refers to other species in the binary mixture, that is, j≠i. Note that in Equation (137) we have introduced the shorthand notation

(142) $$\Delta^{*}\frac{\partial }{\partial \Delta^{*}}\equiv \left(\Delta_{11}^{*}\frac{\partial }{\partial \Delta_{11}^{*}}+\Delta_{22}^{*}\frac{\partial }{\partial \Delta_{22}^{*}}+\Delta_{12}^{*}\frac{\partial }{\partial \Delta_{12}^{*}}\right).$$

In the particular case $\Delta_{11}^{*}=\Delta_{22}^{*}=\Delta_{12}^{*}=\Delta^{*}$, only one of the three terms of the identity (142) must be considered.

The coefficients of the field gradients on the right side of Equations (135)–(139) are functions of the peculiar velocity and the hydrodynamic fields. They are given by

(143) $$A_{i}\left(V\right)=-{\left(\frac{\partial }{\partial x_{1}}f_{i}^{\left(0\right)}\right)}_{p,T}V,\;B_{i}\left(V\right)=-\frac{\partial f_{i}^{\left(0\right)}}{\partial p}V-\rho^{-1}\frac{\partial f_{i}^{\left(0\right)}}{\partial V},$$

(144) $$C_{i}\left(V\right)=-\frac{\partial f_{i}^{\left(0\right)}}{\partial T}V,\;D_{i,\lambda \beta }\left(V\right)=V_{\lambda }\frac{\partial f_{i}^{\left(0\right)}}{\partial V_{\beta }},$$

(145) $$E_{i}\left(V\right)=-\frac{1}{d}\Delta^{*}\frac{\partial f_{i}^{\left(0\right)}}{\partial \Delta^{*}}-\frac{1}{2}\zeta_{U}\left[\frac{\partial }{\partial V}\cdot \left(Vf_{i}^{\left(0\right)}\right)+\Delta^{*}\frac{\partial f_{i}^{\left(0\right)}}{\partial \Delta^{*}}\right].$$

As in the case of monocomponent gases, the Navier–Stokes transport coefficients of the granular mixture can be expressed in terms of the solutions to the set of coupled linear integral Equations (135)–(139). However, as usual, to obtain explicit forms for these transport coefficients one has to resort to the leading terms in a Sonine polynomial expansion of the unknowns $A_{i}$, $B_{i}$, $C_{i}$, $D_{i,\lambda \beta }$, and $E_{i}$. Given that this task is relatively long and tedious, for the sake of illustration, we offer here the determination of the mass flux transport coefficients with some detail.

### 6.2. Diffusion Transport Coefficients

To first order, the mass flux $j_{1}^{\left(1\right)}$ is

(146) $$j_{1}^{\left(1\right)}=-\frac{m_{1}m_{2}n}{\rho }D\nabla x_{1}-\frac{\rho }{p}D_{p}\nabla p-\frac{\rho }{T}D_{T}\nabla T,$$

where *D* is the diffusion coefficient, $D_{p}$ is the pressure diffusion coefficient, and $D_{T}$ is the thermal diffusion coefficient. According to the definition (106) of the mass flux, the diffusion transport coefficients are identified as

(147) $$D=-\frac{1}{d}\frac{\rho }{m_{2}n}\int dv\;V\;\cdot \;A_{1},\;\;D_{p}=-\frac{1}{d}\frac{m_{1}p}{\rho }\int dv\;V\;\cdot \;B_{1},\;\;D_{T}=-\frac{1}{d}\frac{m_{1}T}{\rho }\int dv\;V\;\cdot C_{1}.$$

From the comparison of Equations (134)–(137), it is expected that the vectorial quantities $A_{i}$, $B_{i}$, and $C_{i}$ are proportional to $A_{i}$, $B_{i}$, and $C_{i}$, respectively. Thus, they are directed along V [see Equations (143) and (144)]. As a consequence, to get the diffusion transport coefficients we consider the following lowest order Sonine polynomial approximations for $A_{i}$, $B_{i}$, and $C_{i}$:

(148) $$\left(\begin{array}{c} A_{i}\\ B_{i}\\ C_{i} \end{array}\right)⟶f_{i,M}V\left(\begin{array}{c} a_{i}\\ b_{i}\\ c_{i} \end{array}\right),$$

where

(149) $$f_{i,M}\left(V\right)=n_{i}{\left(\frac{m_{i}}{2\pi T_{i}^{\left(0\right)}}\right)}^{d/2}\exp\left(-\frac{m_{i}V^{2}}{2T_{i}^{\left(0\right)}}\right)$$

is the Maxwellian distribution characterized by the zeroth-order partial temperature $T_{i}^{\left(0\right)}$. The coefficients $a_{i}$, $b_{i}$, and $c_{i}$ are related in this approximation to the transport coefficients *D*, $D_{p}$, and $D_{T}$ through Equations (147) as

(150) $$a_{1}=-\frac{n_{2}T_{2}^{\left(0\right)}}{n_{1}T_{1}^{\left(0\right)}}a_{2}=-\frac{m_{1}m_{2}n}{\rho n_{1}T_{1}^{\left(0\right)}}D,$$

(151) $$b_{1}=-\frac{n_{2}T_{2}^{\left(0\right)}}{n_{1}T_{1}^{\left(0\right)}}b_{2}=-\frac{\rho }{pn_{1}T_{1}^{\left(0\right)}}D_{p},$$

(152) $$c_{1}=-\frac{n_{2}T_{2}^{\left(0\right)}}{n_{1}T_{1}^{\left(0\right)}}c_{2}=-\frac{\rho }{Tn_{1}T_{1}^{\left(0\right)}}D_{T}.$$

In Equations (150)–(152), we have taken into account the constraint $n_{1}T_{1}^{\left(0\right)}+n_{2}T_{2}^{\left(0\right)}=nT=p$.

The coefficients *D*, $D_{p}$, and $D_{T}$ can be determined by substitution of Equation (148) into the integral Equations (135)–(137). Next, one multiplies both sides of these equations by $m_{i}V$ and integrates over v. After some algebra, one gets

(153) $$\begin{array}{ccc} \left[-\frac{1}{2}\zeta^{\left(0\right)}\left(1-\Delta^{*}\frac{\partial \ln D^{*}}{\partial \Delta^{*}}\right)+\nu_{D}\right]D & = & \frac{\rho }{m_{1}m_{2}n}[{\left(\frac{\partial }{\partial x_{1}}n_{1}T_{1}^{\left(0\right)}\right)}_{p,T}\\ & & +\rho {\left(\frac{\partial \zeta^{\left(0\right)}}{\partial x_{1}}\right)}_{p,T}\left(D_{p}+D_{T}\right)], \end{array}$$

(154) $$\left[\frac{1}{2}\zeta^{\left(0\right)}\left(1+\Delta^{*}\frac{\partial \ln D_{p}^{*}}{\partial \Delta^{*}}\right)-2\zeta^{\left(0\right)}+\nu_{D}\right]D_{p}=\frac{n_{1}T_{1}^{\left(0\right)}}{\rho }\left(1-\frac{m_{1}nT}{\rho T_{1}^{\left(0\right)}}\right)+\zeta^{\left(0\right)}D_{T},$$

(155) $$\;\;\;\;\left[\frac{1}{2}\zeta^{\left(0\right)}\Delta^{*}\left(\frac{\partial \ln D_{T}^{*}}{\partial \Delta^{*}}+\frac{\partial \ln\zeta_{0}^{*}}{\partial \Delta^{*}}\right)+\nu_{D}\right]D_{T}=-\frac{n_{1}T}{2\rho }\Delta^{*}\frac{\partial \gamma_{1}}{\partial \Delta^{*}}-\frac{\zeta^{\left(0\right)}}{2}\left(1+\Delta^{*}\frac{\partial \ln\zeta_{0}^{*}}{\partial \Delta^{*}}\right)D_{p}.$$

In Equations (153)–(155), $\zeta_{0}^{*}=\zeta^{\left(0\right)}/\nu$, the collision frequency $\nu_{D}$ is defined as

(156) $$\nu_{D}=-\frac{1}{dn_{1}T_{1}^{\left(0\right)}}\int dv_{1}m_{1}V_{1}\cdot \left(J_{12}\left[f_{1,M}V_{1},f_{2}^{\left(0\right)}\right]-\frac{n_{1}T_{1}^{\left(0\right)}}{n_{2}T_{2}^{\left(0\right)}}J_{12}\left[f_{1}^{\left(0\right)},f_{2,M}V_{2}\right]\right),$$

and the derivatives with respect to $x_{1}$ at constant pressure and temperature are given by

(157) $${\left(\frac{\partial }{\partial x_{1}}n_{1}T_{1}^{\left(0\right)}\right)}_{p,T}=p\left(\gamma_{1}+x_{1}\frac{\partial \gamma_{1}}{\partial x_{1}}\right),$$

(158) $${\left(\frac{\partial \zeta^{\left(0\right)}}{\partial x_{1}}\right)}_{p,T}=\nu \left[{\left(\frac{\partial \zeta_{0}^{*}}{\partial x_{1}}\right)}_{\gamma_{1}}+\frac{\partial \zeta_{0}^{*}}{\partial \gamma_{1}}\frac{\partial \gamma_{1}}{\partial x_{1}}\right].$$

Note that $\gamma_{2}=\left(1-x_{1}\gamma_{1}\right)/\left(1-x_{1}\right)$ and, hence, $\partial_{x_{1}}\gamma_{2}$ can be easily expressed in terms of $\partial_{x_{1}}\gamma_{1}$. In addition, upon obtaining Equations (153)–(155), we have introduced the dimensionless transport coefficients

(159) $$D^{*}=\frac{m_{1}m_{2}\nu }{\rho T}D,\;D_{p}^{*}=\frac{\rho \nu }{nT}D_{p},\;D_{T}^{*}=\frac{\rho \nu }{nT}D_{T},$$

and have used the relations

(160) $$\left(T\frac{\partial }{\partial T}+p\frac{\partial }{\partial p}\right)D=\frac{D}{2}\left(1-\Delta^{*}\frac{\partial \ln D^{*}}{\partial \Delta^{*}}\right),$$

(161) $$\left(T\frac{\partial }{\partial T}+p\frac{\partial }{\partial p}\right)\frac{\rho }{p}D_{p}=-\frac{\rho }{2p}D_{p}\left(1+\Delta^{*}\frac{\partial \ln D_{p}^{*}}{\partial \Delta^{*}}\right),$$

(162) $$\left(T\frac{\partial }{\partial T}+p\frac{\partial }{\partial p}\right)\frac{\rho }{T}D_{T}=-\frac{\rho }{2T}D_{T}\left(1+\Delta^{*}\frac{\partial \ln D_{T}^{*}}{\partial \Delta^{*}}\right).$$

### 6.3. Steady State Conditions

As in the case of the monocomponent granular gas, to achieve analytical expressions of the transport coefficients in the Delta-collisional model one has to consider the steady state conditions. They are defined by the constraints $\zeta_{1}^{\left(0\right)}=\zeta_{2}^{\left(0\right)}=\zeta^{\left(0\right)}=0$. Thus, in the steady state, Equations (153)–(155) become simply linear algebraic equations whose solutions for the dimensionless transport coefficients are

(163) $$D_{p}^{*}=\frac{x_{1}}{\nu_{D}^{*}}\left(\gamma_{1}-\frac{\mu }{x_{2}+\mu x_{1}}\right),$$

(164) $$D_{T}^{*}=-\frac{x_{1}\Delta^{*}\left(\frac{\partial \gamma_{1}}{\partial \Delta^{*}}\right)+\Delta^{*}\left(\frac{\partial \zeta_{0}^{*}}{\partial \Delta^{*}}\right)D_{p}^{*}}{2\nu_{D}^{*}+\Delta^{*}\left(\frac{\partial \zeta_{0}^{*}}{\partial \Delta^{*}}\right)},$$

(165) $$D^{*}=\frac{\gamma_{1}+x_{1}\left(\frac{\partial \gamma_{1}}{\partial x_{1}}\right)+\left(D_{p}^{*}+D_{T}^{*}\right)\left(\frac{\partial \zeta_{0}^{*}}{\partial x_{1}}\right)}{\nu_{D}^{*}},$$

where $\nu_{D}^{*}=\nu_{D}/\nu$ and $\mu =m_{1}/m_{2}$ is the mass ratio. The derivatives $\partial_{x_{1}}\gamma_{1}$, $\partial_{x_{1}}\zeta_{0}^{*}$, $\partial_{\Delta^{*}}\gamma_{1}$, and $\partial_{\Delta^{*}}\zeta_{0}^{*}$ are determined in Appendix A in the particular case $\Delta_{11}=\Delta_{22}=\Delta_{12}$. Additionally, the expression of $\nu_{D}^{*}$ when $f_{i}^{\left(0\right)}$ is replaced by its Maxwellian form $f_{i,M}$ is [145]

(166) $$\nu_{D}^{*}=\frac{2\pi^{(d-1)/2}}{d\Gamma \left(\frac{d}{2}\right)}\left(x_{1}\mu_{12}+x_{2}\mu_{21}\right)\left[{\left(\frac{\theta_{1}+\theta_{2}}{\theta_{1}\theta_{2}}\right)}^{1/2}\left(1+\alpha_{12}\right)+\sqrt{\pi }\Delta_{12}^{*}\right].$$

The constraints $j_{1}^{\left(1\right)}=-j_{2}^{\left(1\right)}$ and $\nabla x_{1}=-\nabla x_{2}$ necessarily imply that *D* must be symmetric while $D_{p}$ and $D_{T}$ must be antisymmetric with respect to the change 1↔2. This can be easily verified from Equations (163)–(165) by noting that $x_{1}\gamma_{1}+x_{2}\gamma_{2}=1$ and $x_{1}\partial \gamma_{1}/\partial \Delta^{*}=-x_{2}\partial \gamma_{2}/\partial \Delta^{*}$. This shows the self-consistency of the expressions found for the diffusion transport coefficients.

### 6.4. Pressure Tensor and Heat Flux

The first-order contribution to the pressure tensor is

(167) $$P_{\lambda \beta }^{\left(1\right)}=-\eta \left(\nabla_{\lambda }U_{\beta }+\nabla_{\beta }U_{\lambda }-\frac{2}{d}\delta_{\lambda \beta }\nabla \cdot U\right),$$

while the heat flux $q^{\left(1\right)}$ is

(168) $$q^{\left(1\right)}=-T^{2}D^{\prime \prime }\nabla x_{1}-L\nabla p-\kappa \nabla T.$$

In Equations (167) and (168), η is the shear viscosity coefficient, $D^{\prime \prime }$ is the Dufour coefficient, *L* is the pressure energy coefficient, and κ is thermal conductivity coefficient.

The shear viscosity η is defined as

(169) $$\eta =-\frac{1}{\left(d-1)(d+2\right)}\sum_{i=1}^{2}\;\int dv\;V_{\lambda }V_{\beta }D_{i,\lambda \beta }\left(V\right).$$

The evaluation of η follows similar mathematical steps to those made for the diffusion transport coefficients, although the calculations are a bit more complex. The shear viscosity of the mixture is $\eta =\eta_{1}+\eta_{2}$, where the partial contributions $\eta_{i}$ are defined as

(170) $$\eta_{i}=-\frac{1}{\left(d-1)(d+2\right)}\int dv\;V_{\lambda }V_{\beta }D_{i,\lambda \beta }\left(V\right).$$

The leading Sonine approximation for the unknown $D_{i,\lambda \beta }$ is

(171) $$D_{i,\lambda \beta }\left(V\right)\to -\frac{m_{i}\eta_{i}}{n_{i}T_{i}^{(0)2}}\left(V_{\lambda }V_{\beta }-\frac{1}{d}\delta_{\lambda \beta }V^{2}\right)f_{i,M}\left(V\right).$$

The next step is to substitute Equation (171) into the integral Equation (138) and, then, multiply it by $m_{i}\left(V_{\lambda }V_{\beta }-\frac{1}{d}\delta_{\lambda \beta }V^{2}\right)$, sum over the repeated indices λ and β, and integrate over velocity. After some algebra, in the steady state, the dimensionless shear viscosity coefficient $\eta^{*}=\left(\nu /p\right)\eta$ is [145]

(172) $$\eta^{*}=\frac{\left(\tau_{22}^{*}-\tau_{21}^{*}\right)x_{1}\gamma_{1}+\left(\tau_{11}^{*}-\tau_{12}^{*}\right)x_{2}\gamma_{2}}{\tau_{11}^{*}\tau_{22}^{*}-\tau_{12}^{*}\tau_{21}^{*}},$$

where the expressions of $\tau_{ij}^{*}$ are given by Equations (C6)–(C10) of Ref. [145].

The evaluation of the heat flux transport coefficients $D^{\prime \prime }$, *L*, and κ is more involved since it requires going up to the second Sonine approximation. However, it is still possible to obtain simple expressions for these coefficients when the first Sonine approximations (148) are considered for $A_{i}$, $B_{i}$, and $C_{i}$, respectively. In this approximation, the heat flux transport coefficients are proportional to the diffusion transport coefficients and their forms are

(173) $$\left\{D^{\prime \prime },L,\kappa \right\}=\frac{d+2}{2}\left(\frac{\gamma_{1}}{m_{1}}-\frac{\gamma_{2}}{m_{2}}\right)\left\{\frac{nm_{1}m_{2}}{\rho T}D,\frac{\rho }{n}D_{p},\rho D_{T}\right\}.$$

According to Equation (173), for mechanically equivalent components, energy equipartition holds ($\gamma_{1}=\gamma_{2}$) [93] and so the first Sonine approximation to the heat transport coefficients vanishes ($D^{\prime \prime }=L=\kappa =0$). Hence, the forms (173) are not able to reproduce the expression of the heat flux for a single granular gas. Nevertheless, these expressions are consistent in the order of approximation used to obtain the mass flux transport coefficients and, hence, they can be employed in several applications for granular confined mixtures.

### 6.5. Some Illustrative Systems

To illustrate the dependence of the transport coefficients on inelasticity, it is more convenient to plot the transport coefficients in their dimensionless forms. The expressions of the diffusion transport coefficients are given by Equations (163)–(165) while the shear viscosity is given by Equation (172). (As noted in Ref. [136], there is a misprint in the last term of Equation (A8) of Ref. [145] since the term $\partial \zeta_{1}^{*}/\partial \gamma_{1}$ must be replaced by the term $\partial \zeta_{1}^{*}/\partial \Delta^{*}$. This modification causes changes in some of the Figures plotted in Ref. [145]. The plots presented here correct Figures 1, 3, and 7 of Ref. [145]). Moreover, for the sake of simplicity, we will assume the case $\Delta_{11}^{*}=\Delta_{22}^{*}=\Delta_{12}^{*}\equiv \Delta^{*}$ and will take a common coefficient of restitution $\alpha_{11}=\alpha_{22}=\alpha_{12}\equiv \alpha$ in a two-dimensional mixture (d=2). Since, in the steady state, $\Delta^{*}$ is a function of α, $x_{1}$, and the mechanical parameters of the mixture, the parameter space is reduced to three quantities: $\left\{\mu \equiv m_{1}/m_{2},\omega \equiv \sigma_{1}/\sigma_{2},x_{1}\right\}$.

For elastic collisions, $\Delta^{*}\left(1\right)=0$, $T_{1}/T_{2}=1$, $D_{T}^{*}=0$, and

(174) $$D_{p}^{*}\left(1\right)=\frac{x_{1}x_{2}}{\nu_{D}^{*}\left(1\right)}\frac{1-\mu }{1+\left(\mu -1\right)x_{1}},\;D^{*}\left(1\right)=\frac{1}{\nu_{D}^{*}\left(1\right)},\;\nu_{D}^{*}\left(1\right)=\sqrt{2\pi }\frac{x_{1}\mu_{12}+x_{2}\mu_{21}}{\sqrt{\mu_{12}\mu_{21}}}.$$

Since we want to assess the effect of inelasticity on transport properties, as usual we normalize here the transport coefficients with respect to their values for elastic collisions. Since the thermal diffusion coefficient $D_{T}^{*}$ vanishes for elastic collisions in the first Sonine approximations, we have normalized it with respect to $D^{*}\left(1\right)$.

**Figure 8:**
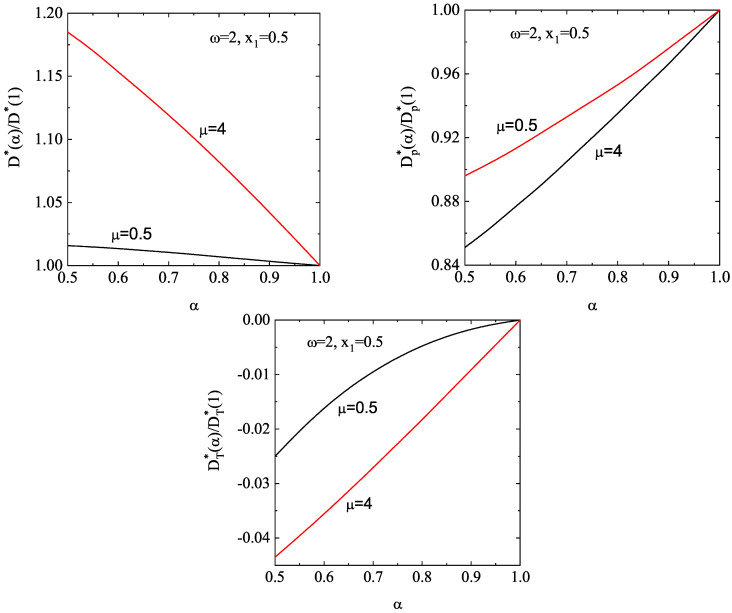
Plots of the (scaled) diffusion transport coefficients $D^{*}\left(\alpha \right)/D^{*}\left(1\right)$, $D_{p}^{*}\left(\alpha \right)/D_{p}^{*}\left(1\right)$, and $D_{T}^{*}\left(\alpha \right)/D^{*}\left(1\right)$ versus the (common) coefficient of restitution α for d=2, ω=2, $x_{1}=\frac{1}{2}$, and two different values of the mass ratio μ: μ=0.5 and μ=4.

Figure 8 shows the α-dependence of the scaled coefficients $D^{*}\left(\alpha \right)/D^{*}\left(1\right)$, $D_{p}^{*}\left(\alpha \right)/D_{p}^{*}\left(1\right)$, and $D_{T}^{*}\left(\alpha \right)/D^{*}\left(1\right)$ for several systems. In general, the effect of inelasticity on mass transport is significant, as the reduced coefficients clearly deviate from their forms for elastic collisions. However, as in the monocomponent limiting case, these deviations are generally less significant than those in the conventional IHS model (see for instance, Figures 1–3 of Ref. [146]). Regarding the dependence on the mass ratio, we observe that, at a given value of α, while the value of $D^{*}\left(\alpha \right)/D^{*}\left(1\right)$ increases with increasing μ, the opposite occurs for the ratio $D_{p}^{*}\left(\alpha \right)/D_{p}^{*}\left(1\right)$. Figure 8 also shows that the thermal diffusion coefficient $D_{T}^{*}$ is always negative (at least in the case $\Delta_{11}^{*}=\Delta_{22}^{*}=\Delta_{12}^{*}$, illustrated here), although its magnitude is quite small. The fact that $D_{T}^{*}$ can be negative is consistent with the results derived in the conventional IHS model. The sign of coefficient $D_{T}^{*}$ is important in problems such as granular segregation by thermal diffusion [125,147,148,149,150,151,152,153,154].

**Figure 9:**
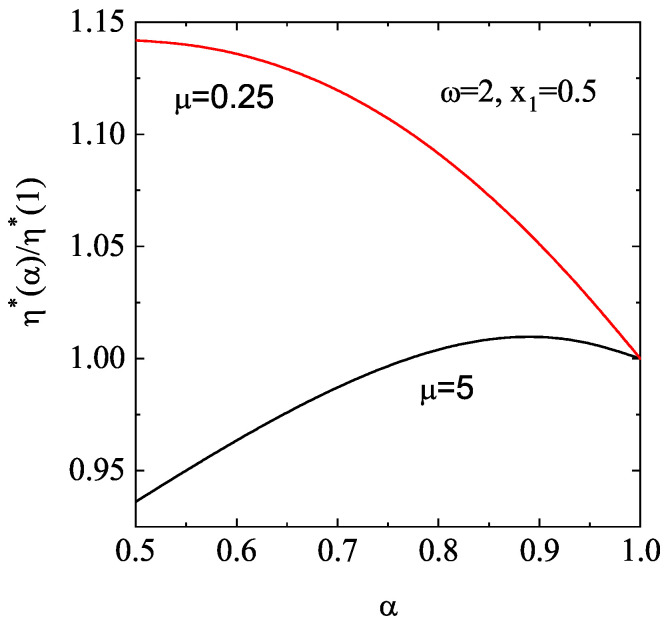
Plot of the (scaled) shear viscosity coefficient $\eta^{*}\left(\alpha \right)/\eta^{*}\left(1\right)$ as a function of the (common) coefficient of restitution α for d=2, ω=2, $x_{1}=0.5$, and two different values of the mass ratio μ: μ=0.25 and μ=5.

The ratio $\eta^{*}\left(\alpha \right)/\eta^{*}\left(1\right)$ is plotted in Figure 9 as a function of the coefficient of restitution α for two different mixtures. As before, $\eta^{*}\left(1\right)$ refers to the shear viscosity coefficient for elastic collisions. As with the diffusion coefficients, we observe that the effect of inelasticity on the shear viscosity is smaller than in the conventional IHS model (see for instance, Figure 5 of Ref. [144]). Additionally, depending on the mass ratio, the normalized shear viscosity decreases (increases) when decreasing α when the mass ratio is larger (smaller) than 1.

In summary, in the context of the Delta-collisional model, the mass and momentum transport coefficients of a (confined) granular mixture differ from those of a molecular mixture, although these deviations are generally less significant than in the IHS model. In most cases, differences with the molecular results increase with increasing dissipation and, depending on the transport coefficient, mass ratio has a significant influence.

## 7. Some Applications of the Kinetic Theory for Granular Mixtures in the Δ-Model

As in the monocomponent case, different problems can be analyzed by using the explicit forms of the Navier–Stokes transport coefficients. In this section, we will study two interesting problems among them. First, we will quantify the violation of Onsager’s well-known reciprocity relations [155] for confined granular mixtures. Since time reversal invariance does not hold for granular gases, we expect that Onsager’s relations will not be verified for finite inelasticity. However, it remains challenging to quantify the deviations from these relations as dissipation increases. Second, we will analyze the stability of the HSS in a granular mixture. As with monocomponent granular gases, our results show that the HSS is linearly stable with respect to perturbations with wavelengths long enough. As expected, however, the forms of the d−1 transversal shear modes and the four longitudinal modes (i.e., those associated with concentration, hydrostatic pressure, temperature, and the longitudinal component of flow velocity) differ from those obtained in the HCS for a granular mixture [146].

### 7.1. Violation of Onsager’s Reciprocity Relations

In the usual language of the linear irreversible thermodynamics for ordinary fluids, to first order in spatial gradients, the constitutive equations for the mass and heat fluxes of a binary mixture are written as [155]

(175) $$j_{i}=-\sum_{j}L_{ij}{\left(\frac{\nabla \mu_{j}}{T}\right)}_{T}-L_{iq}\frac{\nabla T}{T^{2}}-C_{p}\nabla p,$$

(176) $$J_{q}=q-\frac{d+2}{2}T\frac{m_{2}-m_{1}}{m_{1}m_{2}}j_{1}=-L_{qq}\nabla T-\sum_{i}L_{qi}{\left(\frac{\nabla \mu_{i}}{T}\right)}_{T}-C_{p}^{\prime }\nabla p,$$

where $\mu_{i}$ is the chemical potential of the species *i* per unit mass. In the low-density regime,

(177) $${\left(\frac{\nabla \mu_{i}}{T}\right)}_{T}=\frac{1}{m_{i}}\nabla \ln\left(x_{i}p\right).$$

The reason for introducing the heat flow $J_{q}$ is because for elastic collisions this flow is conjugate to the temperature gradient in the form of entropy production [155]. The difference between q and $J_{q}$ is a term associated with diffusion.

The coefficients $L_{ij}$, $L_{iq}$, $C_{p}$, $L_{qq}$, $L_{qi}$, and $C_{p}^{\prime }$ are the so-called Onsager phenomenological coefficients. For ordinary or molecular fluids ($\alpha_{ij}=1$), Onsager showed that time reversal invariance of the underlying microscopic equations of motion leads to the following relations:

(178) $$L_{ij}=L_{ji},\;L_{iq}=L_{qi},\;C_{p}=C_{p}^{\prime }=0.$$

The first two symmetries are called reciprocal relations as they relate transport coefficients for different processes. The coefficients $L_{qi}$ link the mass flux to the thermal gradient while the coefficients $L_{iq}$ link the heat flux to the gradient of the chemical potentials. The last two identities ($C_{p}=0$ and $C_{p}^{\prime }=0$) are statements that the pressure gradient does not appear in any of the fluxes even though it is admitted by symmetry. In particular, the condition $C_{p}^{\prime }=0$ is important for monocomponent elastic gases since it yields Fourier’s law for heat flux ($q^{\left(1\right)}\propto \nabla T$) and, hence, there is no contribution to the heat flux proportional to the density gradient ∇n. On the contrary, for the IHS model, $C_{p}^{\prime }\neq 0$ and there is an additional contribution to the heat flux proportional to ∇n, as discussed in Section 4.2 and Section 4.4 [51,71,138,156].

To quantify the possible violation of Onsager’s relations, we have to express first the Onsager coefficients ($L_{ij}$, $L_{1q}$, $C_{p}$, $L_{qq}$, $L_{q1}$, and $C_{p}^{\prime }$) in terms of both the diffusion (*D*, $D_{p}$, $D_{T}$) and heat flux ($D^{\prime \prime }$, *L*, κ) transport coefficients. To do it, since $\nabla x_{1}=-\nabla x_{2}$, Equation (177) yields the relation

(179) $$\frac{{\left(\nabla \mu_{1}\right)}_{T}-{\left(\nabla \mu_{2}\right)}_{T}}{T}=\frac{n\rho }{\rho_{1}\rho_{2}}\left[\nabla x_{1}+\frac{n_{1}n_{2}}{n\rho }\left(m_{2}-m_{1}\right)\nabla \ln p\right].$$

The relationships between the Onsager coefficients $L_{ij}$ and the diffusion and heat flux transport coefficients are

(180) $$L_{11}=-L_{12}=-L_{21}=\frac{m_{1}m_{2}\rho_{1}\rho_{2}}{\rho^{2}}D,\;L_{1q}=\rho TD_{T},$$

(181) $$L_{q1}=-L_{q2}=\frac{T^{2}\rho_{1}\rho_{2}}{n\rho }D^{\prime \prime }-\frac{d+2}{2}\frac{T\rho_{1}\rho_{2}}{\rho^{2}}\left(m_{2}-m_{1}\right)D,$$

(182) $$L_{qq}=\kappa -\frac{d+2}{2}\rho \frac{m_{2}-m_{1}}{m_{1}m_{2}}D_{T},\;C_{p}\equiv \frac{\rho }{p}D_{p}-\frac{\rho_{1}\rho_{2}}{p\rho^{2}}\left(m_{2}-m_{1}\right)D,$$

(183) $$C_{p}^{\prime }\equiv L-\frac{d+2}{2}T\frac{m_{2}-m_{1}}{m_{1}m_{2}}C_{p}-\frac{n_{1}n_{2}}{np\rho }T^{2}\left(m_{2}-m_{1}\right)D^{\prime \prime }.$$

As said before, since *D* is symmetric under the change 1↔2, then Onsager’s relation $L_{12}=L_{21}$ trivially holds. To analyze the other relations, we define the dimensionless functions

(184) $$P\left(\alpha_{ij}\right)\equiv \left(\frac{\gamma_{1}}{\mu_{12}}-\frac{\gamma_{2}}{\mu_{21}}-\frac{m_{2}^{2}-m_{1}^{2}}{m_{1}m_{2}}\right)D^{*}-\frac{2}{d+2}\frac{(m_{1}+m_{2})n\rho }{\rho_{1}\rho_{2}}D_{T}^{*},$$

and

(185) $$Q\left(\alpha_{ij}\right)\equiv D_{p}^{*}-\frac{\rho_{1}\rho_{2}}{n\rho }\frac{m_{2}-m_{1}}{m_{1}m_{2}}D^{*}.$$

Note that to obtain Equations (184) and (185) use of Equation (173) has been made. The function *P* vanishes when $L_{1q}=L_{q1}$ while the function *Q* vanishes when $C_{p}=0$. Finally, when $C_{p}=0$ and $C_{p}^{\prime }=0$, the function

(186) $$R\left(\alpha_{ij}\right)\equiv \left[\mu_{21}\left(1-\gamma_{1}\right)-\mu_{12}\left(1-\gamma_{2}\right)\right]Q\left(\alpha_{ij}\right)$$

equals zero.

For elastic collisions, $D_{T}^{*}=0$ and $D_{p}^{*}$ and $D^{*}$ are given by Equation (174). Using these expressions yields the expected results: P(1)=Q(1)=R(1)=0. Also, for mechanically equivalent particles with arbitrary α, $D_{p}^{*}=D_{T}^{*}=0$ and, therefore, *P*, *Q*, and *R* vanish as well. However, beyond these two limiting cases, Onsager’s relations do not apply as expected. The origin of this failure is essentially due to (i) the absence of energy equipartition in granular mixtures ($T_{1}^{\left(0\right)}\neq T_{2}^{\left(0\right)}$) and (ii) the homogeneous time-dependent reference state which gives contributions to diffusion coefficients coming from the derivatives ${\left(\partial_{x_{1}}\gamma_{1}\right)}_{s}$, ${\left(\partial_{\Delta^{*}}\gamma_{1}\right)}_{s}$, ${\left(\partial_{x_{1}}\zeta_{i}^{*}\right)}_{s}$, and ${\left(\partial_{\Delta^{*}}\zeta_{i}^{*}\right)}_{s}$. Because energy non-equipartition is involved in determining the above derivatives, it is difficult to disentangle the impact of each effect on violating Onsager’s relations.

**Figure 10:**
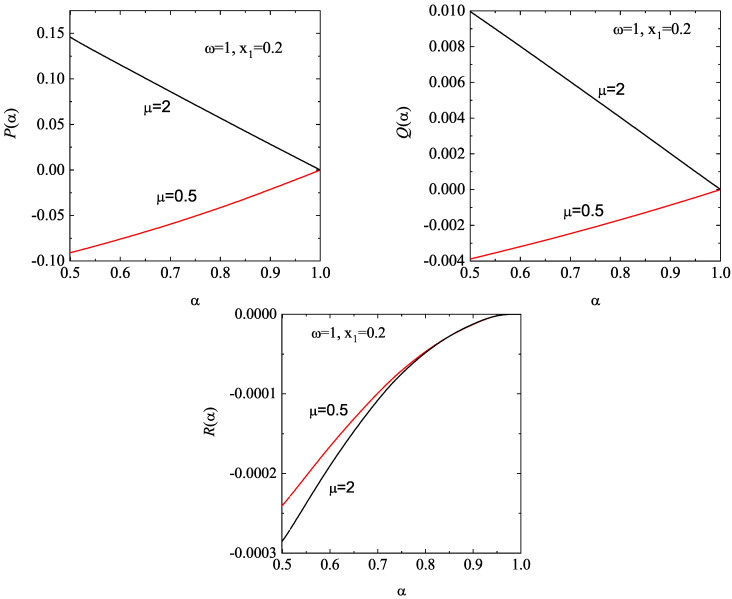
Plot of the dimensionless coefficients P(α), Q(α), and R(α) versus the (common) coefficient of restitution $\alpha_{ij}\equiv \alpha$ for d=2, $x_{1}=0.2$, ω=1, and two different values of the mass ratio μ: μ=0.5 and μ=0.2.

To illustrate the deviations from Onsager’s relations, Figure 10 shows the dependence of the quantities *P*, *Q*, and *R* on the (common) coefficient of restitution $\alpha_{ij}\equiv \alpha$ for d=2, $x_{1}=0.2$, ω=1, and two different values of the mass ratio. Violation of Onsager’s relations is especially evident in the case of the function *P*. The departure from zero is very small for *Q* and *R*, even in cases of strong dissipation. This implies that $C_{p}$ and $C_{p}^{\prime }$ are small. The main conclusion of this subsection is that deviations from Onsager’s relations in the Delta-collisional model are much smaller than in the IHS model for the same systems (see Figures 7–9 of Ref. [146]).

### 7.2. Stability Analysis of the HSS in Granular Mixtures

As a second application, we perform a linear stability analysis of the Navier–Stokes hydrodynamic equations in the case of a binary granular mixture. Thus, the question is whether and to what extent the conclusions about the stability of the HSS in the monocomponent case can be changed for mixtures.

As in Section 4.2, we assume that the deviations $\delta y_{\beta }\left(r,t\right)=y_{\beta }\left(r,t\right)-y_{H\beta }$ are small, where $\delta y_{\beta }\left(r,t\right)$ denotes the deviation of $\left\{x_{1},U,p,T,\right\}$ from their values in the HSS. For the sake of convenience and to compare with the stability analysis performed from the IHS model [146], we introduce the same time and space dimensionless variables as in Ref. [146]: $\tau =\nu_{H}t$ and $\ell =n_{H}\sigma_{12}^{d-1}r$. Here, $\nu_{H}=n_{H}\sigma_{12}^{d-1}v_{\mathrm{th},H}\left(T_{H}\right)$, where $v_{\mathrm{th},H}=\sqrt{2T_{H}/\bar{m}}$ is the thermal velocity of the binary mixture. Note that, for mechanically equivalent particles, these dimensionless variables differ from those used in the stability analysis of Section 4.5.

As usual, the linearized hydrodynamic equations for the perturbations

$$\left\{\delta x_{1}\left(r;t\right),\delta U\left(r;t\right),\delta p\left(r;t\right),\delta T\left(r;t\right)\right\}$$

are written in the Fourier space. A set of Fourier transformed dimensionless variables are introduced as

(187) $$\rho_{k}\left(\tau \right)=\frac{\delta x_{1k}\left(\tau \right)}{x_{1H}},\;w_{k}\left(\tau \right)=\frac{\delta U_{k}\left(\tau \right)}{v_{\mathrm{th},H}},\;\Pi_{k}\left(\tau \right)=\frac{\delta p_{k}\left(\tau \right)}{p_{H}},\;\theta_{k}\left(\tau \right)=\frac{\delta T_{k}\left(\tau \right)}{T_{H}},$$

where $p_{H}=n_{H}T_{H}$. Here, $\delta y_{k\beta }\equiv \left\{\delta x_{1k}\left(\tau \right),w_{k}\left(\tau \right),\Pi_{k}\left(\tau \right),\theta_{k}\left(\tau \right)\right\}$ is defined as

(188) $$\delta y_{k\beta }\left(\tau \right)=\int dr^{\prime }\;e^{-ık\cdot r^{\prime }}\delta y_{\beta }\left(r^{\prime },\tau \right).$$

As in the stability analysis carried out in Section 4.5 for monocomponent gases, the subscript H denotes the HSS.

After writing the corresponding linearized version of the Navier–Stokes hydrodynamic equations in the Fourier space, it is quite apparent that the d−1 transverse velocity components $w_{k⊥}=w_{k}-\left(w_{k}\cdot \hat{k}\right)\hat{k}$ (orthogonal to the wave vector k) decouple from the other four modes and they verify d−1 differential equations given by

(189) $$\frac{\partial w_{k⊥}}{\partial \tau }+\frac{1+\mu }{4(x_{1}\mu +x_{2})}\eta^{*}k^{2}w_{k⊥}=0.$$

Since $\eta^{*}$ does not depend on time in the HSS, the solution to Equation (189) is

(190) $$w_{k⊥}\left(\tau \right)=w_{k⊥}\left(0\right)\exp\left(-\frac{1+\mu }{4(x_{1}\mu +x_{2})}\eta^{*}k^{2}\tau \right).$$

Thus, the d−1 transversal shear modes $w_{k⊥}\left(\tau \right)$ are linearly stable because the shear viscosity $\eta^{*}$ is always positive [see Equation (172)].

The set of differential equations for the four longitudinal modes $\rho_{k}$, $\theta_{k}$, $\Pi_{k}$, and $w_{k||}$ (parallel to k) is more intricate. In matrix form, this set can be written as [99]

(191) $$\frac{\partial \delta z_{k\alpha }\left(\tau \right)}{\partial \tau }=\left(M_{\alpha \beta }^{\left(0\right)}+ıkM_{\alpha \beta }^{\left(1\right)}+k^{2}M_{\alpha \beta }^{\left(2\right)}\right)\delta z_{k\beta }\left(\tau \right),$$

where now $\delta z_{k\alpha }\left(\tau \right)$ denotes the four variables $\left(\rho_{k},\theta_{k},\Pi_{k},w_{k||}\right)$. The matrices in Equation (191) are

(192) $$M^{\left(0\right)}=\left(\begin{array}{cccc} 0 & 0 & 0 & 0\\ -A & B & 0 & 0\\ -A & B & 0 & 0\\ 0 & 0 & 0 & 0 \end{array}\right),$$

(193) $$M^{\left(1\right)}=\left(\begin{array}{cccc} 0 & 0 & 0 & 0\\ 0 & 0 & 0 & -\left(\frac{2}{d}+\zeta_{U}\right)\\ 0 & 0 & 0 & -\left(\frac{d+2}{d}+\zeta_{U}\right)\\ 0 & 0 & -\frac{1}{4}\frac{1+\mu }{x_{1}\mu +x_{2}} & 0 \end{array}\right),$$

(194) $$M^{\left(2\right)}=\left(\begin{array}{cccc} -\frac{1}{4}\frac{\mu x_{1}+x_{2}}{\mu_{12}}D^{*} & -\frac{1}{4x_{1}}\frac{\mu x_{1}+x_{2}}{\mu_{12}}D_{T}^{*} & -\frac{1}{4x_{1}}\frac{\mu x_{1}+x_{2}}{\mu_{12}}D_{p}^{*} & 0\\ x_{1}\left(\frac{1-\mu }{4\mu_{12}}D^{*}-\frac{1}{d}{D^{\prime \prime }}^{*}\right)\; & \frac{1-\mu }{4\mu_{12}}D_{T}^{*}-\frac{1}{d}\kappa^{*} & \frac{1-\mu }{4\mu_{12}}D_{p}^{*}-\frac{1}{d}L^{*} & 0\\ -\frac{1}{d}x_{1}{D^{\prime \prime }}^{*} & -\frac{1}{d}\kappa^{*}\; & -\frac{1}{d}L^{*} & 0\\ 0 & 0 & 0 & -\frac{d-1}{2d}\frac{1+\mu }{\mu x_{1}+x_{2}}\eta^{*} \end{array}\right).$$

In Equation (192), we have introduced the (dimensionless) quantities

(195) $$A=x_{1}\left\{\left(\frac{\partial \zeta_{2}^{*}}{\partial x_{1}}\right)+x_{1}\gamma_{1}\left[\left(\frac{\partial \zeta_{1}^{*}}{\partial x_{1}}\right)-\left(\frac{\partial \zeta_{2}^{*}}{\partial x_{1}}\right)\right]\right\},$$

(196) $$B=\frac{1}{2}\Delta_{s}^{*}\left\{\left(\frac{\partial \zeta_{2}^{*}}{\partial \Delta^{*}}\right)+x_{1}\gamma_{1}\left[\left(\frac{\partial \zeta_{1}^{*}}{\partial \Delta^{*}}\right)-\left(\frac{\partial \zeta_{2}^{*}}{\partial \Delta^{*}}\right)\right]\right\}.$$

Additionally, the dimensionless heat flux transport coefficients are defined as

(197) $${D^{\prime \prime }}^{*}=\frac{\bar{m}\nu }{n}D^{\prime \prime },\;L^{*}=\frac{\bar{m}\nu }{T}L,\;\kappa^{*}=\frac{\bar{m}\nu }{p}\kappa .$$

As in the case of the transverse modes, the subscript H has been suppressed in Equations (192)–(197) for the sake of brevity. For mechanically equivalent particles, $D_{p}^{*}=D_{T}^{*}=0$, which implies $L^{*}=\kappa^{*}=0$ in the first Sonine approximation. Moreover, in this limiting case, A=0, $B=\left(\Delta^{*}/2\right)\left(\partial \zeta_{0}^{*}/\partial \Delta^{*}\right)$, and the results are consistent with those obtained in Section 4.5 for monocomponent granular gases [130].

The time evolution of the four longitudinal modes has the form $e^{s_{n}\left(k\right)\tau }$ (n=1, 2, 3, and 4), where the quantities $s_{n}\left(k\right)$ are the eigenvalues of the matrix $M_{\alpha \beta }\equiv M_{\alpha \beta }^{\left(0\right)}+ıkM_{\alpha \beta }^{\left(1\right)}+k^{2}M_{\alpha \beta }^{\left(2\right)}$. In other words, the eigenvalues $s_{n}\left(k\right)$ are the solutions of the quartic equation

(198) $$\det\left(M-s1\right)=0,$$

where 1 is the matrix identity. The determination of the dependence of the eigenvalues $s_{n}\left(k\right)$ on the (dimensionless) wave vector *k* and the parameters of the mixture is a quite intricate problem. Therefore, to gain some insight into the general problem, it is convenient to study first the solution to quartic Equation (198) in the extreme long wavelength limit, k=0.

When k=0, the matrix M reduces to $M^{\left(0\right)}$ whose eigenvalues are $s_{\left|\right|}=\left\{0,0,0,B\right\}$. According to Equation (196), the dependence of *B* on the parameter space of the system is in general complex. A simple situation corresponds to the case of mechanically equivalent particles where $\zeta_{1}^{*}=\zeta_{2}^{*}=\zeta_{0}^{*}$ and, so, in the steady state

(199) $$\frac{\partial \zeta_{0}^{*}}{\partial \Delta^{*}}=-\frac{1}{2}\left(\sqrt{2\pi }\alpha +4\Delta^{*}\right).$$

**Figure 11:**
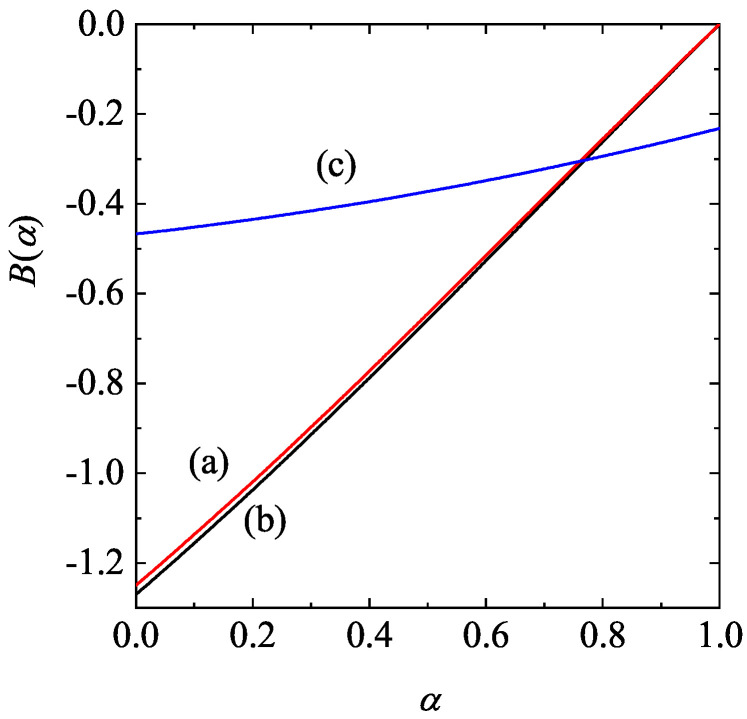
Dependence of the eigenvalue *B* on coefficient of restitution α for a two-dimensional system and three different granular binary mixtures: $x_{1}=0.5$, ω=0.5, μ=0.75, and $\alpha_{ij}\equiv \alpha$ (a); $x_{1}=0.5$, ω=2, μ=2, and $\alpha_{ij}\equiv \alpha$ (b); and $x_{1}=0.2$, ω=1, μ=0.2, $\alpha_{22}$ = 0.8, $\alpha_{11}\equiv \alpha$, and $\alpha_{12}=\left(\alpha_{22}+\alpha \right)/2$ (c).

Thus, in this limiting case

(200) $$B=-\frac{1}{4}\Delta^{*}\left(\sqrt{2\pi }\alpha +4\Delta^{*}\right)<0,$$

and the longitudinal modes are linearly stable in agreement with previous results [130]. In the case of mechanically different particles, a detailed study of the dependence of the quantity *B* on the parameters of the mixture for the choice $\Delta_{ij}=\Delta$ shows that *B* is always negative. As a consequence, all the longitudinal modes in a granular mixture are stable when k=0 in the Δ-model. As an illustration, in Figure 11 we plot the dependence of *B* on the coefficient of restitution α for three different mixtures. We clearly observe that the eigenvalue *B* is always negative; its magnitude increases with decreasing α.

**Figure 12:**
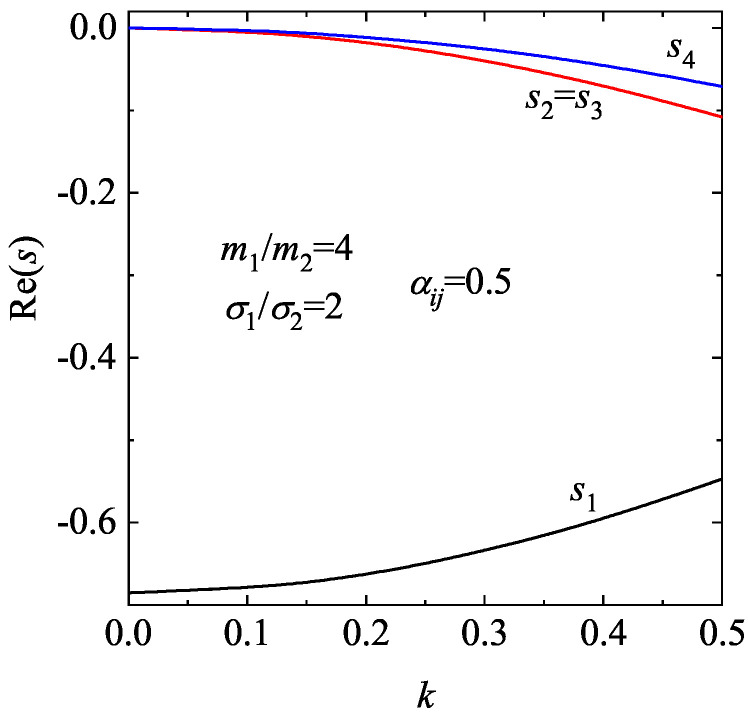
Real parts of the longitudinal eigenvalues $s_{i}$ as functions of the wave number *k* for a two-dimensional granular binary mixture with $x_{1}=0.5$, ω=2, μ=4 and the (common) coefficient of restitution $\alpha_{ij}\equiv 0.5$.

Beyond the limit k→0, the eigenvalues of M must be numerically determined. In the case $\Delta_{ij}\equiv \Delta$, a careful study of the dependence of the eigenvalues of the matrix M on the parameters of the mixture shows that the real part of *all* the eigenvalues is *negative* and, hence, the HSS is linearly stable in the complete range of values of the wave number *k* studied [99]. This result contrasts with that obtained in the conventional IHS model [146]. As an illustration, Figure 12 shows the real parts of the eigenvalues $s_{i}$ (i=1,2,3,4) as functions of the (dimensionless) wave number *k* for a two-dimensional granular binary mixture with a concentration $x_{1}=0.5$, a diameter ratio $\sigma_{1}/\sigma_{2}=2$, a mass ratio $m_{1}/m_{2}=4$, and a (common) coefficient of restitution $\alpha_{ij}=0.5$. As in the case of monocomponent gases, we observe that two of the modes (denoted as $s_{2}$ and $s_{3}$) are a complex conjugate pair of propagating modes [Re(s2)=Re(s3)] while the other two modes ($s_{1}$ and $s_{4}$) are real for all values of the wave number. We see that the magnitude of $s_{4}$ is very small while the mode $s_{1}$ ($s_{2}$) increases (decreases) with increasing the wave number. Although not shown in the figure, we also observe that the influence of the disparity in masses and/or diameters does not play a relevant role on the dependence of the eigenvalues $s_{i}$ on the wave number *k* since the results for monocomponent granular gases are quite close to the ones found for bidisperse systems.

## 8. Concluding Remarks

In this review, we have presented a comprehensive account of the kinetic theory of the Δ-collisional model for driven granular gases. The model was originally introduced as a minimal way to incorporate collisional energy injection into the dynamics of granular particles, particularly in situations that mimic vertically vibrated and confined systems. The main feature of the Δ-model is that energy injection is implemented directly at the level of binary collisions, in a way that exactly conserves momentum, in contrast with other thermostats, like Gaussian or random thermostats. This mechanism leads to a nonequilibrium steady state that is found to be homogeneous, where collisional dissipation (determined by the normal restitution coefficient, α) and energy injection (modeled by the parameter Δ) balance each other. This homogeneous steady state provides a reference state for hydrodynamic expansions and stability analyses. This is in contrast with freely cooling granular gases, whose intrinsic time dependence, due to dissipation of energy, complicates the derivation of transport properties. The structure of the injection term via binary collisions retains the usual form of the collision operator for hard spheres in the kinetic equations, supplemented with terms dependent on the Δ parameter. This formal similarity has enabled the application of standard tools of kinetic theory, including Sonine polynomial expansions and Chapman–Enskog methods, to derive Navier–Stokes transport coefficients and characterize the hydrodynamic fields. As usual in granular gases, there is a new contribution to the heat current. It is proportional to the gradient of density and the transport coefficient is the *diffusive heat conductivity* coefficient, μ. This term is in addition to the usual term, proportional to the gradient of temperature with thermal conductivity coefficient κ. Analytical expressions for all transport coefficients are obtained by considering the leading terms of the Sonine polynomial expansions. Of course, the resulting transport coefficients depend in a nontrivial way on both the coefficient of restitution and the driving parameter. There may be alternative routes to calculate transport coefficients, using Green–Kubo formulas adapted to dissipative dynamics [157,158] or Helfand formulas [159], and it would be interesting to compare those results with the ones derived here.

Linear stability analysis of the hydrodynamic modes carried out in Section 4.5 and Section 7.2 demonstrates that the collisional driving modifies the long-wavelength behavior of the system, leading to the conclusion that the homogeneous state is stable. The spectrum of hydrodynamic modes reflects the stabilizing role of collisional injection. This is in contrast with freely cooling systems, where clustering and shear instabilities emerge in the system. The Δ parameter stabilizes the system and prevents clusters or other instabilities to appear. Physically, the origin of the stability at large wavelengths is that in the Δ-model the stationary temperature turns out to be density independent. This results in the pressure, which is the product of the temperature and a function of density, being a monotonically increasing function of density, hence displaying a positive compressibility. The linear stability of the HSS means that the Δ-model in its present form cannot reproduce clustering instabilities observed experimentally in driven granular monolayers. Understanding whether clustering can emerge through finite-size effects, boundary-induced inhomogeneities, or regimes beyond the Navier–Stokes approximation is an intriguing open question. Extensions incorporating a density-dependent or velocity-dependent Δ parameter could potentially make the stationary temperature decrease with increasing density, generating a van der Waals loop, thereby admitting clustering while retaining much of the analytical structure. Some steps in this direction are already given in Ref. [75].

The model allows a generalization to include different types of particles to study the behavior of mixtures, with a stronger phenomenology. When two or more species are present, distinguished by any of their dynamical properties (mass, diameter, restitution coefficient or energy injection one), the system exhibits a breakdown of energy equipartition: each species reaches a different granular temperature in the steady state, a typical signature (or consequence) of the nonequilibrium nature of the granular fluids. The Enskog equation is generalized to mixtures and allows computing partial temperatures, which, in the present review, are compared with computer simulations of both the Event Driven type (MD simulations) and DSMC method for a two-component system.

In the case of mixtures, there is a new balance equation for the density of each species in addition to momentum and energy (or temperature) balance equations. The new equations (or their transforms into concentration and pressure equations) introduce three diffusivities: a diffusion coefficient, a pressure diffusion coefficient and a thermal diffusion coefficient, whose explicit definitions are given in this review. The determination of these transport coefficients opens the way to extending the analysis to segregation phenomena under gravity [100]. In particular, there we study the Brazil nut effect of motion of large particles in the direction of the external gravity field or against it. A specific problem addressed in this review is the violation of Onsager’s reciprocity relations. In the case of elastic collisions (molecular mixtures), such reciprocity relations are derived under the assumption of the time reversibility of microscopic dynamics. However, this is not the case in granular systems. However, and probably due to the fact that there is a homogeneous steady state in the Δ-model, the violation of Onsager’s reciprocal relations is much weaker than the case of a pure IHS dynamics, where such a stationary state does not exist.

An important aspect emphasized in some parts of this review is the interplay between analytical theory and numerical simulations. Event Driven MD and DSMC simulations have been used to validate the predictions of kinetic theory. In particular, simulations allow us to identify the limits of common approximations (such as the effects of truncation on low-order Sonine expansions or spatial correlations), and to explore regimes beyond strict hydrodynamic conditions or larger densities, where Enskog equation may fail. Generally speaking, numerical simulations agree well with the analytical results derived from the Enskog equation.

Although the results reported here for transport properties in granular mixtures have been restricted to the dilute regime, progress has recently been made regarding moderate densities. Thus, the tracer diffusion coefficients have been explicitly determined [100] by considering the lowest Sonine polynomial approximation. The extension of these results to arbitrary concentrations has been recently worked out [160], and the forms of the diffusion coefficients, as well as the shear and bulk viscosities, have been obtained. An interesting future project is to determine the heat flux transport coefficients. Knowing the complete set of Navier–Stokes transport coefficients will allow us to analyze the stability of the homogeneous steady state and/or to assess the violation of Onsager’s reciprocity relations for dense granular mixtures, among other applications.

For the sake of simplicity, the results provided here for transport in binary mixtures have been restricted to the case $\Delta_{11}=\Delta_{22}=\Delta_{12}\equiv \Delta$. The extension to the case $\Delta_{11}\neq \Delta_{22}\neq \Delta_{12}$ (namely, when the energy injection depends on the species) is simple but requires additional calculations. We plan to perform this calculation in the near future and assess how the choice of this case affects for instance the stability of the HSS of a granular binary mixture.

Comparison with realistic quasi-two-dimensional experiments and full three-dimensional simulations of the confined geometry also requires more systematic attention. In particular, the precise relationship between effective collisional driving, characterized by the Δ parameter, with realistic boundary forcing in vertically vibrated systems, like amplitude or frequency, deserves further clarification. It is worth noting that, compared to the full quasi-two-dimensional system, the Δ-model makes some idealized approximations that might need to be reconsidered if the predictions of the model are to be compared with experiments. First, the motion of the grains is strictly restricted to the horizontal plane and the vertical motion is eliminated in favor of the added velocity at collisions. This implies that particles collide when their horizontal distance equals the diameter and there is no partial overlap due to three-dimensionality (see Figure 1). As a result, the maximum planar density is slightly smaller than for the quasi-two-dimensional system. Another effect of eliminating the vertical direction is that the particles cannot lock with the wall, which has been observed experimentally and can lead to absorbing and hyperuniform states [104,105,161,162,163]. Similarly, as there is no gradual increase of the energy in the vertical degrees of freedom and the value of Δ is fixed, some microscopic correlations are lost; for example, in the Q2D geometry, after a grain–grain collision they will have smaller vertical velocities, modifying for a short time their collision frequencies and the available energy to be exchanged in subsequent collisions.

Another promising direction concerns strongly inhomogeneous states, such as shear flows, temperature gradients, or confined geometries where boundary layers cannot be neglected. Although the homogeneous steady state provides a convenient reference, many experimentally relevant situations involve spatial gradients that challenge standard hydrodynamic expansions. Exploring non-Newtonian transport, rheological properties, and nonlinear instabilities within the Δ-framework constitutes a natural continuation of the work reviewed here.

In summary, the Δ-model has developed into a coherent and versatile theoretical framework for driven granular fluids. It is an example of how relatively simple modifications of microscopic rules can generate qualitatively new nonequilibrium behavior while remaining amenable to rigorous kinetic analysis. It provides a consistent kinetic and hydrodynamic description, accommodates mixtures and segregation phenomena, and connects naturally with experimental realizations of confined vibrated systems. The continued interest in the model, including recent theoretical and numerical studies, and extensions to the study of other phenomena, underscores its relevance as a reference system for exploring nonequilibrium statistical mechanics in dissipative matter.

## Figures and Tables

**Table 1:** Explicit expressions of the scaled transport coefficients for a two-dimensional monocomponent granular gas (d=2) at the stationary temperature.

$\eta^{*}=\left[1+\frac{1}{2}\phi \chi \left(1+\alpha +\sqrt{\frac{2}{\pi }}\Delta_{M}^{*}\right)\right]\eta_{k}^{*}+\frac{1}{2}\eta_{b}^{*}$,
$\eta_{k}^{*}=\nu_{\eta }^{*-1}\left\{1-\frac{1}{4}\phi \chi \left[\left(1+\alpha \right)\left(1-3\alpha \right)-4\sqrt{\frac{2}{\pi }}\left(1+2\alpha \right)\Delta_{M}^{*}-4\Delta_{M}^{*2}\right]\right\}$,
$\eta_{b}^{*}=\frac{8}{\pi }\phi^{2}\chi \left(1+\alpha +\sqrt{\frac{\pi }{2}}\Delta_{M}^{*}\right)$,
$\kappa^{*}=\left[1+\frac{3}{4}\phi \chi \left(1+\alpha +\sqrt{\frac{2}{\pi }}\Delta_{M}^{*}\right)\right]\kappa_{k}^{*}+\frac{2}{\pi }\phi^{2}\chi \left(1+\alpha +\sqrt{\frac{\pi }{2}}\Delta_{M}^{*}\right)$,
$\begin{array}{c} \kappa_{k}^{*}=\frac{1}{2\nu_{\kappa }^{*}+\Delta_{M}^{*}\left(\frac{\partial \zeta_{0}^{*}}{\partial \Delta^{*}}\right)}\{1+\frac{3}{8}\phi \chi {\left(1+\alpha \right)}^{2}\left(2\alpha -1\right)-\frac{\Delta_{M}^{*}}{\sqrt{2\pi }}\phi \chi \\ \times \left[\frac{3}{4}+3\left(1+\alpha \right)\left(1-\frac{1}{2}\sqrt{2\pi }\Delta_{M}^{*}\right)-\frac{9}{2}{\left(1+\alpha \right)}^{2}-\Delta_{M}^{*2}\right]\}, \end{array}$
$\mu^{*}=\left[1+\frac{3}{4}\phi \chi \left(1+\alpha +\sqrt{\frac{2}{\pi }}\Delta_{M}^{*}\right)\right]\mu_{k}^{*}$,
$\mu_{k}^{*}=-\frac{1}{\nu_{\kappa }^{*}}\phi \chi \left(1+\frac{1}{2}\phi \partial_{\phi }\ln\chi \right)\left\{\frac{3}{8}\alpha \left(1-\alpha^{2}\right)-\frac{\Delta_{M}^{*}}{\sqrt{2\pi }}\phi \chi \left[2\Delta_{M}^{*2}-3\left(\frac{1}{2}-\alpha^{2}\right)+\frac{3}{2}\sqrt{2\pi }\alpha \Delta_{M}^{*}\right]\right\}$,
$\nu_{\eta }^{*}=\frac{3}{8}\chi \left[\left(\frac{7}{3}-\alpha \right)\left(1+\alpha \right)+\frac{2\sqrt{2\pi }}{3}\left(1-\alpha \right)\Delta_{M}^{*}-\frac{2}{3}\Delta_{M}^{*2}\right]$,
$\nu_{\kappa }^{*}=\nu_{\mu }^{*}=\frac{1+\alpha }{2}\chi \left[\frac{1}{2}+\frac{15}{8}\left(1-\alpha \right)\right]-\frac{\Delta_{M}^{*}}{16}\chi \left[\sqrt{2\pi }\left(5\alpha -1\right)+10\Delta_{M}^{*}\right]$,
$\Delta_{M}^{*}\left(\alpha \right)=\frac{1}{2}\sqrt{\frac{\pi }{2}}\alpha \left[\sqrt{1+\frac{4(1-\alpha^{2})}{\pi \alpha^{2}}}-1\right]$,
$p^{*}=1+\phi \chi \left(1+\alpha \right)+2\sqrt{\frac{2}{\pi }}\phi \chi \Delta_{M}^{*}$,
$\chi =\frac{1-\frac{7}{16}\phi }{{\left(1-\phi \right)}^{2}}$.

## Data Availability

The data that support the findings of this study are available from the corresponding author upon reasonable request.

## Appendix A. Some Expressions for Transport in Confined Granular Mixtures

In this Appendix we give some expressions needed to evaluate the diffusion transport coefficients as well as to perform the linear stability analysis of the HSS in a granular binary mixture. In particular, the first-order contribution $\zeta_{U}$ to the energy rate can be written as

(A1) $$\zeta_{U}=\sum_{i=1}^{2}\xi_{i}^{*}\varpi_{i}^{*},$$

where

(A2) $$\begin{array}{ccc} \xi_{i}^{*} & = & \frac{3\pi^{(d-1)/2}}{d\Gamma \left(\frac{d}{2}\right)}\frac{x_{i}m_{i}}{\bar{m}\gamma_{i}}\sum_{j=1}^{2}x_{j}{\left(\frac{\sigma_{ij}}{\sigma_{12}}\right)}^{d-1}\mu_{ji}\left(1-\alpha_{ij}^{2}\right){\left(\theta_{i}+\theta_{j}\right)}^{1/2}\theta_{i}^{-3/2}\theta_{j}^{-1/2}\\ & & -\frac{4\pi^{(d-1)/2}}{d\Gamma \left(\frac{d}{2}\right)}x_{i}\Delta^{*}\sum_{j=1}^{2}x_{j}{\left(\frac{\sigma_{ij}}{\sigma_{12}}\right)}^{d-1}\mu_{ji}\{\sqrt{\pi }\alpha_{ij}+{\left(\theta_{i}+\theta_{j}\right)}^{-1/2}\theta_{i}^{3/2}\theta_{j}^{-1/2}\Delta^{*}\\ & & \times \left[d-d\left(\theta_{i}+\theta_{j}\right)\theta_{i}^{-1}+\left(d+1\right)\theta_{i}\theta_{j}^{-1}\right]\}, \end{array}$$

(A3) $$\varpi_{1}^{*}=\frac{1}{d}\frac{\Delta^{*}\left(\frac{\partial \gamma_{1}}{\partial \Delta^{*}}\right)}{\Lambda_{1}^{*}},\;\varpi_{2}^{*}=-\frac{x_{1}}{x_{2}}\varpi_{1}^{*},$$

(A4) $$\Lambda_{1}^{*}=\omega_{11}^{*}-\frac{x_{1}}{x_{2}}\omega_{12}^{*}-\left[\frac{1}{2}\Delta^{*}\left(\frac{\partial \gamma_{1}}{\partial \Delta^{*}}\right)-\gamma_{1}\right]\left(\xi_{1}^{*}-\frac{x_{1}}{x_{2}}\xi_{2}^{*}\right).$$

In Equation (A9), the expressions of $\omega_{11}^{*}=\omega_{11}/\nu$ and $\omega_{12}^{*}=\omega_{12}/\nu$ can be easily obtained from Equations (C12) and (C13), respectively, of Appendix C of Ref. [145].

According to Equations (163)–(165) and Equations (A3) and (A4), it is quite apparent that the diffusion transport coefficients and the first-order contribution to the rate of energy are given in terms of the derivatives $\left(\partial_{x_{1}}\gamma_{1}\right)$, $\left(\partial_{\Delta^{*}}\gamma_{1}\right)$, $\left(\partial_{x_{1}}\zeta_{i}^{*}\right)$, and $\left(\partial_{\Delta^{*}}\zeta_{i}^{*}\right)$. We recall that these derivatives are evaluated in the steady state. The derivative $\left(\partial \gamma_{1}/\partial \Delta^{*}\right)$ is [136,145]

(A5) $$\left(\frac{\partial \gamma_{1}}{\partial \Delta^{*}}\right)=\frac{\sqrt{Y^{2}-4N\Delta^{*}Z}-Y}{2N\Delta^{*}},$$

where

(A6) $$Y=M\Delta^{*}-2N\gamma_{1}+\gamma_{1}\left(\frac{\partial \zeta_{1}^{*}}{\partial \gamma_{1}}\right),\;Z=\gamma_{1}\left(\frac{\partial \zeta_{1}^{*}}{\partial \Delta^{*}}\right)-2M\gamma_{1}.$$

In Equations (A5) and (A6), we have introduced the quantities

(A7) $$M=\frac{1}{2}\left[x_{1}\gamma_{1}{\left(\frac{\partial \zeta_{1}^{*}}{\partial \Delta^{*}}\right)}_{\gamma_{1}}+x_{2}\gamma_{2}{\left(\frac{\partial \zeta_{2}^{*}}{\partial \Delta^{*}}\right)}_{\gamma_{1}}\right],\;N=\frac{1}{2}\left(x_{1}\gamma_{1}\frac{\partial \zeta_{1}^{*}}{\partial \gamma_{1}}+x_{2}\gamma_{2}\frac{\partial \zeta_{2}^{*}}{\partial \gamma_{1}}\right).$$

In addition, the derivatives $\left(\partial_{\Delta^{*}}\zeta_{i}^{*}\right)$ and $\left(\partial_{x_{1}}\zeta_{i}^{*}\right)$ are given by

(A8) $$\left(\frac{\partial \zeta_{i}^{*}}{\partial \Delta^{*}}\right)={\left(\frac{\partial \zeta_{i}^{*}}{\partial \Delta^{*}}\right)}_{\gamma }+\left(\frac{\partial \zeta_{i}^{*}}{\partial \gamma_{1}}\right)\left(\frac{\partial \gamma_{1}}{\partial \Delta^{*}}\right),\;\left(\frac{\partial \zeta_{i}^{*}}{\partial x_{1}}\right)={\left(\frac{\partial \zeta_{i}^{*}}{\partial x_{1}}\right)}_{\gamma }+\left(\frac{\partial \zeta_{i}^{*}}{\partial \gamma_{1}}\right)\left(\frac{\partial \gamma_{1}}{\partial x_{1}}\right).$$

Finally, the derivative $\partial \gamma_{1}/\partial x_{1}$ can be written as

(A9) $$\left(\frac{\partial \gamma_{1}}{\partial x_{1}}\right)=-\frac{\gamma_{1}\frac{\partial \zeta_{1}^{*}}{\partial x_{1}}+Q\left[\Delta^{*}\left(\frac{\partial \gamma_{1}}{\partial \Delta^{*}}\right)-2\gamma_{1}\right]}{\gamma_{1}\frac{\partial \zeta_{1}^{*}}{\partial \gamma_{1}}+N\left[\Delta^{*}\left(\frac{\partial \gamma_{1}}{\partial \Delta^{*}}\right)-2\gamma_{1}\right]},$$

where

(A10) $$Q=\frac{1}{2}\left(x_{1}\gamma_{1}\frac{\partial \zeta_{1}^{*}}{\partial x_{1}}+x_{2}\gamma_{2}\frac{\partial \zeta_{2}^{*}}{\partial x_{1}}\right).$$

In Equations (A9) and (A10), the derivative $\partial_{x_{1}}\zeta_{i}^{*}$ is taken at $\gamma_{i}\equiv \mathrm{const}$.

## References

[1] Faraday M. (1831). XVII. On a peculiar class of acoustical figures; and on certain forms assumed by groups of particles upon vibrating elastic surfaces. Philos. Trans. R. Soc. Lond..

[2] Campbell C.S. (1990). Rapid granular flows. Annu. Rev. Fluid Mech..

[3] Jaeger H.M., Nagel S.R., Behringer R.P. (1996). Granular solids, liquids, and gases. Rev. Mod. Phys..

[4] Duran J. (1999). Sands, Powders, and Grains: An Introduction to the Physics of Granular Materials.

[5] Goldhirsch I. (2003). Rapid granular flows. Annu. Rev. Fluid Mech..

[6] Aranson I.S., Tsimring L.V. (2006). Patterns and collective behaviour in granular media: Theoretical concepts. Rev. Mod. Phys..

[7] Haff P.K. (1983). Grain flow as a fluid-mechanical phenomenon. J. Fluid Mech..

[8] Brito R., Ernst M.H. (1998). Extension of Haff’s cooling law in granular flows. Europhys. Lett..

[9] Herrmann H.J., Hovi J.P., Luding S. (1998). Physics of Dry Granular Media.

[10] Andreotti B., Forterre Y., Pouliquen O. (2013). Granular Media. Between Fluid and Solid.

[11] Falcon E., Wunenburger R., Èvesque P., Fauve S., Chabot C., Garrabos Y., Beysens D. (1999). Cluster formation in a granular medium fluidized by vibrations in low gravity. Phys. Rev. Lett..

[12] McNamara S., Young W.R. (1994). Inelastic collapse in two dimensions. Phys. Rev. E.

[13] Goldhirsch I., Zanetti G. (1993). Clustering instability in dissipative gases. Phys. Rev. Lett..

[14] van Noije T.P.C., Ernst M.H., Brito R., Orza J.A.G. (1997). Mesoscopic theory of granular fluids. Phys. Rev. Lett..

[15] Aranson I., Tsimring L. (2009). Granular Patterns.

[16] Pouliquen O. (1999). Scaling laws in granular flows down rough inclined planes. Phys. Fluids.

[17] Börzsönyi T., Halsey T.C., Ecke R.E. (2005). Two scenarios for avalanche dynamics in inclined granular layers. Phys. Rev. Lett..

[18] Savage S.B., Lun C.K.K. (1988). Particle size segregation in inclined chute flow of dry cohesionless granular solids. J. Fluid Mech..

[19] Jaeger H.M., Liu C.h., Nagel S.R. (1989). Relaxation at the angle of repose. Phys. Rev. Lett..

[20] Ristow G.H., Straßburger G., Rehberg I. (1997). Phase diagram and scaling of granular materials under horizontal vibrations. Phys. Rev. Lett..

[21] Melo F., Umbanhowar P.B., Swinney H.L. (1995). Hexagons, kinks, and disorder in oscillated granular layers. Phys. Rev. Lett..

[22] Valverde J.M., Castellanos A., Mills P., Quintanilla M.A.S. (2003). Effect of particle size and interparticle force on the fluidization behavior of gas-fluidized beds. Phys. Rev. E.

[23] Rietz F., Radin C., Swinney H.L., Schröter M. (2018). Nucleation in sheared granular matter. Phys. Rev. Lett..

[24] Mullin T. (2000). Coarsening of self-organized clusters in binary mixtures of particles. Phys. Rev. Lett..

[25] Pica Ciamarra M., Coniglio A., Nicodemi M. (2007). Phenomenology and theory of horizontally oscillated granular mixtures. Eur. Phys. J. E.

[26] Hill K.M., Caprihan A., Kakalios J. (1997). Bulk segregation in rotated granular material measured by magnetic resonance imaging. Phys. Rev. Lett..

[27] Kudrolli A. (2004). Size separation in vibrated granular matter. Rep. Prog. Phys..

[28] Olafsen J.S., Urbach J.S. (1998). Clustering, order, and collapse in a driven granular monolayer. Phys. Rev. Lett..

[29] Ciamarra M.P., Coniglio A., Nicodemi M. (2005). Shear instabilities in granular mixtures. Phys. Rev. Lett..

[30] Aumaître S., Schnautz T., Kruelle C.A., Rehberg I. (2003). Granular phase transition as a precondition for segregation. Phys. Rev. Lett..

[31] Schnautz T., Brito R., Kruelle C.A., Rehberg I. (2005). A horizontal Brazil-nut effect and its reverse. Phys. Rev. Lett..

[32] Mujica N., Soto R., Klapp J., Sigalotti L., Medina A., López A., Ruiz-Chavarría G. (2016). Dynamics of noncohesive confined granular media. Recent Advances in Fluid Dynamics with Environmental Applications.

[33] Grossman E.L., Zhou T., Ben-Naim E. (1997). Towards granular hydrodynamics in two dimensions. Phys. Rev. E.

[34] Visco P., Puglisi A., Barrat A., Trizac E., van Wijland F. (2006). Fluctuations of power injection in randomly driven granular gases. J. Stat. Phys..

[35] Visco P., Puglisi A., Barrat A., van Wijland F., Trizac E. (2006). Energy fluctuations in vibrated and driven granular gases. Eur. Phys. J. B.

[36] McNamara S., Barrat J.L. (1997). Energy flux into a fluidized granular medium at a vibrating wall. Phys. Rev. E.

[37] Kumaran V. (1998). Temperature of a granular material “fluidized” by external vibrations. Phys. Rev. E.

[38] Barrat A., Trizac E. (2002). Molecular dynamics simulations of vibrated granular gases. Phys. Rev. E.

[39] Olafsen J.S., Urbach J.S. (2005). Two-dimensional melting far from equilibrium in a granular monolayer. Phys. Rev. Lett..

[40] Prevost A., Melby P., Egolf D.A., Urbach J.S. (2004). Nonequilibrium two-phase coexistence in a confined granular layer. Phys. Rev. E.

[41] Clerc M.G., Cordero P., Dunstan J., Huff K., Mujica N., Risso D., Varas G. (2008). Liquid-solid-like transition in quasi-one-dimensional driven granular media. Nat. Phys..

[42] Castillo G., Mujica N., Soto R. (2012). Fluctuations and criticality of a granular solid-liquid-Like phase transition. Phys. Rev. Lett..

[43] Melby P., Vega Reyes F., Prevost A., Robertson R., Kumar P., Egolf D.A., Urbach J.S. (2005). The dynamics of thin vibrated granular layers. J. Phys. C Condens. Matter.

[44] Roeller K., Clewett J.P.D., Bowley R.M., Herminghaus S., Swift M.R. (2011). Liquid-gas phase separation in confined vibrated dry granular matter. Phys. Rev. Lett..

[45] Clewett J.P.D., Roeller K., Bowley R.M., Herminghaus S., Swift M.R. (2012). Emergent surface tension in vibrated, noncohesive granular media. Phys. Rev. Lett..

[46] Khain E., Aranson I.S. (2011). Hydrodynamics of a vibrated granular monolayer. Phys. Rev. E.

[47] Mayo M., Petit J.C., García Soria M.I., Maynar P. (2023). Confined granular gases under the influence of vibrating walls. J. Stat. Mech..

[48] Brito R., Risso D., Soto R. (2013). Hydrodynamic modes in a confined granular fluid. Phys. Rev. E.

[49] Kadanoff L.P. (1999). Built upon sand: Theoretical ideas inspired by granular flows. Rev. Mod. Phys..

[50] Brilliantov N., Pöschel T. (2004). Kinetic Theory of Granular Gases.

[51] Garzó V. (2019). Granular Gaseous Flows.

[52] Dorfman J., van Beijeren H., Kirkpatrick T. (2021). Contemporary Kinetic Theory of Matter.

[53] Chamorro M.G., González G., Garzó V. (2022). Kinetic theory of polydisperse granular mixtures: Influence of the partial temperatures on transport properties. A review. Entropy.

[54] Maynar P., García de Soria M.I., Brey J.J. (2019). Understanding an instability in vibrated granular monolayers. Phys. Rev. E.

[55] van Noije T.P.C., Ernst M.H. (1998). Velocity distributions in homogeneous granular fluids: The free and heated case. Granul. Matter.

[56] Montanero J., Santos A. (2000). Computer simulation of uniformly heated granular fluids. Granul. Matter.

[57] Williams D.R.M., MacKintosh F.C. (1996). Driven granular media in one dimension: Correlations and equation of state. Phys. Rev. E.

[58] Puglisi A., Loreto V., Marconi U.M.B., Petri A., Vulpiani A. (1998). Clustering and non-Gaussian behavior in granular matter. Phys. Rev. Lett..

[59] Puglisi A., Loreto V., Marconi U.M.B., Vulpiani A. (1999). Kinetic approach to granular gases. Phys. Rev. E.

[60] Peng G., Ohta T. (1998). Steady state properties of a driven granular medium. Phys. Rev. E.

[61] Garzó V., Montanero J.M. (2002). Transport coefficients of a heated granular gas. Physica A.

[62] García de Soria M.I., Maynar P., Trizac E. (2013). Linear hydrodynamics for driven granular gases. Phys. Rev. E.

[63] Garzó V., Chamorro M.G., Vega Reyes F. (2013). Transport properties for driven granular fluids in situations close to homogeneous steady states. Phys. Rev. E.

[64] Khalil N., Garzó V. (2013). Transport coefficients for driven granular mixtures at low-density. Phys. Rev. E.

[65] Khalil N., Garzó V. (2018). Heat flux of driven granular mixtures at low density: Stability analysis of the homogeneous steady state. Phys. Rev. E.

[66] Barrat A., Trizac E., Fuchs J.N. (2001). Heated granular fluids: The random restitution coefficient approach. Eur. Phys. J. E.

[67] Barrat A., Trizac E. (2003). Random inelasticity and velocity fluctuations in a driven granular gas. Eur. Phys. J. E.

[68] Lei Q.L., Ni R. (2019). Hydrodynamics of random-organizing hyperuniform fluids. Proc. Natl. Acad. Sci. USA.

[69] Maire R., Galliano L., Plati A., Berthier L. (2025). Hyperuniform Interfaces in Nonequilibrium Phase Coexistence. Phys. Rev. Lett..

[70] van Noije T.P.C., Ernst M.H., Brito R. (1998). Spatial correlations in compressible granular flows. Phys. Rev. E.

[71] Brey J.J., Dufty J.W., Kim C.S., Santos A. (1998). Hydrodynamics for granular flows at low density. Phys. Rev. E.

[72] Brey J.J., Ruiz-Montero M.J. (2013). Shearing instability of a dilute granular mixture. Phys. Rev. E.

[73] Mitrano P.P., Garzó V., Hrenya C.M. (2014). Instabilities in granular binary mixtures at moderate densities. Phys. Rev. E.

[74] Chapman S., Cowling T.G. (1970). The Mathematical Theory of Nonuniform Gases.

[75] Risso D., Soto R., Guzmán R. (2018). Effective two-dimensional model for granular matter with phase separation. Phys. Rev. E.

[76] Soto R., Risso D., Brito R. (2014). Shear viscosity of a model for confined granular media. Phys. Rev. E.

[77] Brey J.J., Maynar P., García de Soria M.I., Buzón V. (2014). Homogeneous hydrodynamics of a collisional model of confined granular gases. Phys. Rev. E.

[78] Brey J.J., Buzón V., Maynar P., García de Soria M. (2015). Hydrodynamics for a model of a confined quasi-two-dimensional granular gas. Phys. Rev. E.

[79] Brey J.J., Buzón V., García de Soria M.I., Maynar P. (2016). Stability analysis of the homogeneous hydrodynamics of a model for a confined granular gas. Phys. Rev. E.

[80] Maynar P., García de Soria I., Brey J.J. (2019). Homogeneous dynamics in a vibrated granular monolayer. J. Stat. Mech..

[81] Brey J.J., de Soria M.I.G., Maynar P., Buzón V. (2014). Memory effects in the relaxation of a confined granular gas. Phys. Rev. E.

[82] Brey J.J., Buzón V., Maynar P., García de Soria M.I. (2017). Kinetic theory of a confined quasi-two-dimensional gas of hard spheres. Entropy.

[83] Lasanta A., Vega Reyes F., Prados A., Santos A. (2017). When the Hotter Cools More Quickly: Mpemba Effect in Granular Fluids. Phys. Rev. Lett..

[84] Garzó V., Brito R., Soto R. (2018). Enskog kinetic theory for a model of a confined quasi-two-dimensional granular fluid. Phys. Rev. E.

[85] Jenkins J.T., Mancini F. (1987). Balance laws and constitutive relations for plane flows of a dense, binary mixture of smooth, nearly elastic, circular disks. J. Appl. Mech..

[86] Garzó V., Dufty J.W. (1999). Homogeneous cooling state for a granular mixture. Phys. Rev. E.

[87] Feitosa K., Menon N. (2002). Breakdown of energy equipartition in a 2D binary vibrated granular gas. Phys. Rev. Lett..

[88] Clelland R., Hrenya C.M. (2002). Simulations of a binary-sized mixture of inelastic grains in rapid shear flow. Phys. Rev. E.

[89] Wildman R.D., Parker D.J. (2002). Coexistence of two granular temperatures in binary vibrofluidized beds. Phys. Rev. Lett..

[90] Huthmann M., Zippelius A. (1997). Dynamics of inelastically colliding rough spheres: Relaxation of translational and rotational energy. Phys. Rev. E.

[91] McNamara S., Luding S. (1998). Energy nonequipartition in systems of inelastic, rough spheres. Phys. Rev. E.

[92] Cafiero R., Luding S., Herrmann H.J. (2002). Rotationally driven gas of inelastic rough spheres. Europhys. Lett..

[93] Brito R., Soto R., Garzó V. (2020). Energy nonequipartition in a collisional model of a confined quasi-two-dimensional granular mixture. Phys. Rev. E.

[94] Rosato A., Strandburg K.J., Prinz F., Swendsen R.H. (1987). Why the Brazil nuts are on top: Size segregation of particulate matter by shaking. Phys. Rev. Lett..

[95] Huerta D.A., Ruiz-Suárez J.C. (2004). Vibration-induced granular segregation: A phenomenon driven by three mechanisms. Phys. Rev. Lett..

[96] Breu A.P.J., Ensner H.M., Kruelle C.A., Rehberg I. (2003). Reversing the Brazil-nut effect: Competition between percolation and condensation. Phys. Rev. Lett..

[97] Shinbrot T. (2004). The Brazil nut effect—In reverse. Nature.

[98] Garzó V. (2008). A note on the violation of the Einstein relation in a driven moderately dense granular gas. J. Stat. Mech..

[99] Garzó V., Brito R., Soto R. (2024). Applications of the kinetic theory for a model of a confined quasi-two dimensional granular binary mixture: Stability analysis and thermal diffusion segregation. Phys. Fluids.

[100] Gómez González R., Garzó V., Brito R., Soto R. (2024). Diffusion of impurities in a moderately dense confined granular gas. Phys. Fluids.

[101] Plati A., Maire R., Fayen E., Boulogne F., Restagno F., Smallenburg F., Foffi G. (2024). Quasi-crystalline order in vibrating granular matter. Nat. Phys..

[102] Joyce M., Morand J., Viot P. (2016). Attractor nonequilibrium stationary states in perturbed long-range interacting systems. Phys. Rev. E.

[103] Maire R., Plati A. (2024). Enhancing (quasi-)long-range order in a two-dimensional driven crystal. J. Chem. Phys..

[104] Maire R., Plati A., Smallenburg F., Foffi G. (2025). Dynamical and structural properties of an absorbing phase transition: A case study from granular systems. J. Stat. Mech..

[105] Maire R., Plati A., Stockinger M., Trizac E., Smallenburg F., Foffi G. (2024). Interplay between an absorbing phase transition and synchronization in a driven granular system. Phys. Rev. Lett..

[106] Maire R., Plati A., Smallenburg F., Foffi G. (2025). Non-equilibrium coexistence between a fluid and a hotter or colder crystal of granular hard disks. J. Chem. Phys..

[107] Liu R., Yang M., Chen K. (2025). Hyperuniform mixing of binary active spinners. Soft Matter.

[108] Maire R., Petrini A., Marconi U.M.B., Caprini L. (2026). Kinetic Theory of chiral active disks: Odd transport and torque density. arXiv.

[109] Lutsko J.F. (2004). Kinetic theory and hydrodynamics of dense, reacting fluids far from equilibrium. J. Chem. Phys..

[110] Ferziger J.H., Kaper G.H. (1972). Mathematical Theory of Transport Processes in Gases.

[111] Brey J.J., García de Soria M.I., Maynar P., Buzón V. (2013). Homogeneous steady state of a confined granular gas. Phys. Rev. E.

[112] Garzó V., Dufty J.W. (1999). Dense fluid transport for inelastic hard spheres. Phys. Rev. E.

[113] Lutsko J.F. (2005). Transport properties of dense dissipative hard-sphere fluids for arbitrary energy loss models. Phys. Rev. E.

[114] Bird G.A. (1994). Molecular Gas Dynamics and the Direct Simulation Monte Carlo of Gas Flows.

[115] Garzó V., Santos A. (2003). Kinetic Theory of Gases in Shear Flows. Nonlinear Transport.

[116] Soto R. (2016). Kinetic Theory and Transport Phenomena.

[117] Brey J.J., Ruiz-Montero M.J., Cubero D. (1999). On the validity of linear hydrodynamics for low-density granular flows described by the Boltzmann equation. Europhys. Lett..

[118] Brey J.J., Ruiz-Montero M.J., Cubero D., García-Rojo R. (2000). Self-diffusion in freely evolving granular gases. Phys. Fluids.

[119] Brey J.J., Ruiz-Montero M.J., Moreno F. (2001). Hydrodynamics of an open vibrated granular system. Phys. Rev. E.

[120] Dahl S.R., Hrenya C.M., Garzó V., Dufty J.W. (2002). Kinetic temperatures for a granular mixture. Phys. Rev. E.

[121] Lutsko J.F., Brey J.J., Dufty J.W. (2002). Diffusion in a granular fluid. II. Simulation. Phys. Rev. E.

[122] Montanero J.M., Garzó V. (2002). Monte Carlo simulation of the homogeneous cooling state for a granular mixture. Granul. Matter.

[123] Montanero J.M., Garzó V. (2003). Shear viscosity for a heated granular binary mixture at low density. Phys. Rev. E.

[124] Garzó V., Montanero J.M. (2003). Shear viscosity for a moderately dense granular binary mixture. Phys. Rev. E.

[125] Brey J.J., Ruiz-Montero M.J., Moreno F. (2005). Energy partition and segregation for an intruder in a vibrated granular system under gravity. Phys. Rev. Lett..

[126] Lois G., Lemaître A., Carlson J.M. (2007). Spatial force correlations in granular shear flow. II. Theoretical implications. Phys. Rev. E.

[127] Mitrano P.P., Dhal S.R., Cromer D.J., Pacella M.S., Hrenya C.M. (2011). Instabilities in the homogeneous cooling of a granular gas: A quantitative assessment of kinetic-theory predictions. Phys. Fluids.

[128] Chialvo S., Sundaresan S. (2013). A modified kinetic theory for frictional granular flows in dense and dilute regimes. Phys. Fluids.

[129] Chamorro M.G., Garzó V. (2023). Assessment of kinetic theories for moderately dense granular binary mixtures: Shear viscosity coefficient. Phys. Fluids.

[130] Garzó V., Brito R., Soto R. (2021). Stability of the homogeneous steady state for a model of a confined quasi-two-dimensional granular fluid. EPJ Web Conf..

[131] de Groot S.R., van Leeuwen W.A., van Weert C.G. (1980). Relativistic Kinetic Theory: Principles and Applications.

[132] Cercignani C., Kremer G. (2002). The Relativistic Boltzmann Equation: Theory and Applications.

[133] Pérez-Fuentes C., Garzó V. (2014). Influence of a drag force on linear transport in low-density gases. Stability analysis. Physica A.

[134] Garzó V., Brito R., Soto R. (2020). Erratum: Enskog kinetic theory for a model of a confined quasi-two-dimensional granular fluid. Phys. Rev. E.

[135] Garzó V., Brito R., Soto R. (2026). Enskog kinetic theory for a model of a confined quasi-two-dimensional granular fluid. arXiv.

[136] Garzó V., Brito R., Soto R. (2024). Erratum: Applications of the kinetic theory for a model of a confined quasi-two dimensional granular binary mixture: Stability analysis and thermal diffusion segregation. Phys. Fluids.

[137] Almazán L., Salueña C., Garzó V., Pöschel T. (2013). A numerical study of the Navier–Stokes transport coefficients for two-dimensional granular hydrodynamics. New J. Phys..

[138] Soto R., Mareschal M., Risso D. (1999). Departure from Fourier’s law for fluidized granular media. Phys. Rev. Lett..

[139] Garzó V. (2005). Instabilities in a free granular fluid described by the Enskog equation. Phys. Rev. E.

[140] Résibois P., de Leener M. (1977). Classical Kinetic Theory of Fluids.

[141] Garzó V., Dufty J.W., Hrenya C.M. (2007). Enskog theory for polydisperse granular mixtures. I. Navier–Stokes order transport. Phys. Rev. E.

[142] Barrat A., Trizac E. (2002). Lack of energy equipartition in homogeneous heated binary granular mixtures. Granul. Matter.

[143] Garzó V., Dufty J.W. (2002). Hydrodynamics for a granular binary mixture at low density. Phys. Fluids.

[144] Garzó V., Montanero J.M. (2007). Navier–Stokes transport coefficients of *d*-dimensional granular binary mixtures at low-density. J. Stat. Phys..

[145] Garzó V., Brito R., Soto R. (2021). Navier–Stokes transport coefficients for a model of a confined quasi-two dimensional granular binary mixture. Phys. Fluids.

[146] Garzó V., Montanero J.M., Dufty J.W. (2006). Mass and heat fluxes for a binary granular mixture at low density. Phys. Fluids.

[147] Jenkins J.T., Yoon D.K. (2002). Segregation in binary mixtures under gravity. Phys. Rev. Lett..

[148] Brey J.J., Ruiz-Montero M.J., Moreno F. (2006). Hydrodynamic profiles for an impurity in an open vibrated granular gas. Phys. Rev. E.

[149] Serero D., Goldhirsch I., Noskowicz S.H., Tan M.L. (2006). Hydrodynamics of granular gases and granular gas mixtures. J. Fluid Mech..

[150] Garzó V. (2006). Segregation in granular binary mixtures: Thermal diffusion. Europhys. Lett..

[151] Garzó V. (2008). Brazil-nut effect versus reverse Brazil-nut effect in a moderately granular dense gas. Phys. Rev. E.

[152] Brito R., Enríquez H., Godoy S., Soto R. (2008). Segregation induced by inelasticity in a vibrofluidized granular mixture. Phys. Rev. E.

[153] Garzó V. (2009). Segregation by thermal diffusion in moderately dense granular mixtures. Eur. Phys. J. E.

[154] Garzó V. (2011). Thermal diffusion segregation in granular binary mixtures described by the Enskog equation. New J. Phys..

[155] de Groot S.R., Mazur P. (1984). Nonequilibrium Thermodynamics.

[156] Candela D., Walsworth R.L. (2007). Understanding the breakdown of Fourier’s law in granular fluids. Am. J. Phys..

[157] Ernst M.H., Brito R. (2005). Generalized Green-Kubo formulas for fluids with impulsive, dissipative, stochastic, and conservative interactions. Phys. Rev. E.

[158] Ernst M.H., Brito R. (2005). New Green-Kubo formulas for transport coefficients in hard-sphere, Langevin fluids and the likes. Europhys. Lett..

[159] Dufty J.W., Baskaran A., Brey J.J. (2008). Linear response and hydrodynamics for granular fluids. Phys. Rev. E.

[160] González Méndez D., Garzó V. (2026). Transport properties in a model of confined granular mixtures at moderate densities. Phys. Fluids.

[161] Rivas N., Ponce S., Gallet B., Risso D., Soto R., Cordero P., Mujica N. (2011). Sudden chain energy transfer events in vibrated granular media. Phys. Rev. Lett..

[162] Néel B., Rondini I., Turzillo A., Mujica N., Soto R. (2014). Dynamics of a first-order transition to an absorbing state. Phys. Rev. E.

[163] Le Blay M., Saldi J.H., Morin A. (2025). Control of collective activity to crystallize an oscillator gas. Nat. Phys..

